# Rediscovery by Whole Genome Sequencing: Classical Mutations and Genome Polymorphisms in *Neurospora crassa*

**DOI:** 10.1534/g3.111.000307

**Published:** 2011-09-01

**Authors:** Kevin McCluskey, Aric E. Wiest, Igor V. Grigoriev, Anna Lipzen, Joel Martin, Wendy Schackwitz, Scott E. Baker

**Affiliations:** * Fungal Genetics Stock Center, School of Biological Sciences, University of Missouri-Kansas City, Kansas City, Missouri 64110; † U.S. Department of Energy Joint Genome Institute, Walnut Creek, California 94598, and; ‡ Chemical and Biological Process Development Group, Pacific Northwest National Laboratory, Richland, Washington 99354

**Keywords:** single nucleotide polymorphism, SNP, indel, comparative genomics, classical mutant

## Abstract

Classical forward genetics has been foundational to modern biology, and has been the paradigm for characterizing the role of genes in shaping phenotypes for decades. In recent years, reverse genetics has been used to identify the functions of genes, via the intentional introduction of variation and subsequent evaluation in physiological, molecular, and even population contexts. These approaches are complementary and whole genome analysis serves as a bridge between the two. We report in this article the whole genome sequencing of eighteen classical mutant strains of *Neurospora crassa* and the putative identification of the mutations associated with corresponding mutant phenotypes. Although some strains carry multiple unique nonsynonymous, nonsense, or frameshift mutations, the combined power of limiting the scope of the search based on genetic markers and of using a comparative analysis among the eighteen genomes provides strong support for the association between mutation and phenotype. For ten of the mutants, the mutant phenotype is recapitulated in classical or gene deletion mutants in *Neurospora* or other filamentous fungi. From thirteen to 137 nonsense mutations are present in each strain and indel sizes are shown to be highly skewed in gene coding sequence. Significant additional genetic variation was found in the eighteen mutant strains, and this variability defines multiple alleles of many genes. These alleles may be useful in further genetic and molecular analysis of known and yet-to-be-discovered functions and they invite new interpretations of molecular and genetic interactions in classical mutant strains.

*Neurospora crassa* has been used as a genetic model system since the early 1930s (Lindegren *et al.* 1939) because it presented significant advantages for genetics research. It grows on chemically defined culture medium, has a haploid vegetative stage, can complete a full sexual generation in under a month, and produces ordered ascospores (the haploid products of meiosis), facilitating genetic analysis (Davis and Perkins 2002). *Neurospora* was quickly adopted by a number of laboratories and is famous for the demonstration of the “one-gene, one-enzyme hypothesis” (Beadle and Tatum 1941), which showed that classical mutations that could be followed in a genetic cross were associated with biochemical traits that could be characterized chemically. By the time the Fungal Genetics Stock Center (FGSC) was established in 1960 it was estimated that hundreds of genetic loci were being investigated. The classical genetic map of *N. crassa* now comprises over 1,000 phenotypic markers and an additional several hundred genetic markers such as telomeres, centromeres, the nucleolus organizer, and a variety of translocations, inversions and duplications (Perkins *et al.* 2001). Because of its many advantages and the relative ease with which one could produce biochemical mutants, *N. crassa* was used for research into every aspect of genetics and biochemistry. *N. crassa* grows as a haploid filament that makes copious asexual spores in vegetative culture, and when compatible strains are paired, it can make either vegetative hetero-dikaryons or go into the sexual cycle. To facilitate allelism tests, strains that were vegetatively compatible and displayed high fertility were generated at Yale and at Oak Ridge National Laboratory, where *Neurospora* was utilized in extensive radiation biology studies (De Serres and Webber 1997). By 1963, much of the research community had adopted this so-called “Oak Ridge” lineage. Therefore, many mutants in the FGSC collection have a shared lineage (e.g., Perkins *et al*. 1962). This lineage gave rise to the current wild type strains used in both the *Neurospora* genome sequencing project (Galagan *et al.* 2003) and in the *Neurospora* functional genomics program (Colot *et al.* 2006; Dunlap *et al.* 2007). Additionally, a compendium of *Neurospora* genes, first published in 1982 (Perkins *et al.* 1982) and updated in 2001 (Perkins *et al.* 2001), is currently maintained as an electronic compendium online (http://bmbpcu36.leeds.ac.uk/~gen6ar/newgenelist/genes/gene_list.htm).

Despite detailed genetic, physiological and biochemical characterization, many classical mutants remain anonymous at the level of the genome sequence. However, because of the extensive genetic mapping done with *N. crassa*, most classical markers are flanked by genes that have been identified to the level of DNA sequence. This makes it possible to exclude most of the genome from consideration when looking to associate a genetic marker with the open reading frame (ORF) responsible for the mutant phenotype in any given strain. Whether the approach is chromosome walking with cosmids that are mapped onto the genome sequence, or by gene sequencing, the high resolution *N. crassa* genetic map allows one to use flanking markers to delimit the search. This approach has been used at the FGSC to identify four temperature sensitive lethal mutations by gene complementation (Dieterle *et al.* 2010; McCluskey *et al.* 2007). Advances in genome sequencing technology (Hobert 2010) have made it possible to sequence the entire genome to identify individual mutations (Le Crom *et al.* 2009; Pomraning *et al.* 2011; Sarin *et al.* 2010; Smith *et al.* 2008). For *Neurospora*, this is complicated by the fact that not all classical genetic mutations have been crossed into the same genetic background as that of the reference genome strain. Eight of the strains sequenced in the current report were explicitly backcrossed into the reference genome prior to deposit into the FGSC collection. Ten were reported to have ‘mixed’ backgrounds.

*Neurospora* is a cosmopolitan fungus and it has been isolated from locations all over the world (Turner *et al.* 2001). While most genetic research is carried out with *N. crassa*, other species are commonly isolated from the environment and have unique characteristics. *Neurospora intermedia* is interfertile with both *N. tetrasperma* and *N. crassa*, and progeny can be recovered from crosses carried out in the laboratory. One such cross was carried out to allow a meiotic drive element, *Sporekiller-2* (*Sk-2*), rarely identified in wild isolates of *N. intermedia*, to be studied in *N. crassa* (Turner and Perkins 1979).

The FGSC collection holds and distributes a large number of morphological and developmental mutants. A subset of these for which the underlying nature of the gene defect remains unknown were chosen for this study (Table 1). In this article, we report the whole genome sequencing of seventeen classical mutant strains of *N. crassa*, and the putative identification of classical mutations in sixteen of them. An eighteenth strain that carries the *Sk-2* meiotic drive element, originally identified in the related species *N. intermedia* and subsequently introgressed into *N. crassa*, was also sequenced. Overall, these mutant strains exhibit a wide range of sequence variability that is directly proportional to their being related to the reference genome strain. Much of the variation is shared among strains while other variation is strain-specific. Some regions of the genome show enrichment for unique variation suggesting that they are hot-spots for mutation. Insertions and deletions manifest a strong size bias associated with their presence in coding *vs.* non-coding DNA. The ability to compare among multiple strains and exclude shared variants aids in the association between neutral polymorphism and phenotypically relevant mutations.

**Table 1:** Strains of *Neurospora crassa* with their relevant genetic characteristics

FGSC No.	Gene	Contig	Flanking Markers	Range on Contig	No. of Genes in Range	Mutagen	Genetic Background*^a^*	Reference	Genbank SRA #
106	*Com*	3	*ace-2*, *ad-4*	1565000 to 1827000	44	UV	SL3	(Perkins and Ishitani 1959)	SRP004312
305	*amyc*	1	*ad-5*, *cen-I*	2988000 to 3730000	166	?	SL3	(Atwood and Mukai 1954)	SRP004308
309	*Ti*	1	*arg-3*, *T(39311*)	3000000 to 3730000	163	X-rays	SL3	(Perkins 1959)	SRP004304
322	*ty-1*	3	*tyr-1*, *cen-3*	3000000 to 4500000	396	Spontaneous	M	(Horowitz *et al.* 1961)	SRP003564
821	*ts*	5	*inl*, *cen-V*	3000000 to 5000000	514	Spontaneous	M	(Nakamura and Egashira 1961)	SRP002767
1211	*dot*	1	*ad-9*, *thi-1*	3970000 to 5658063	415	Spontaneous	SL3	(Perkins *et al.* 1962)	SRP002761
1303	*Fi*	4	*pyr-1*, *ace-4*	432932 to 1547255	234	Spontaneous	M	(Perkins *et al.* 1962)	SRP002757
1363	*smco-1*	1	*mat*, *rg-1*	2500000 to 4500000	409	Mustard	L	(Garnjobst and Tatum 1967)	SRP004305
2261	*do*	7	*nic-3*, *cen-7*	>3475561	193	UV	SL2	(Perkins *et al.* 1962)	SRP004307
3114	*Sk-2*	3	*cum*, *his-7*	70000 to 2040000	407	Introgression	SL	(Turner and Perkins 1979)	SRP002747
3246	*fs-n*	1	*mat*, *T(4637) al-1*	1860000 to 1940000	18	Spontaneous	M	(Mylyk and Threlkeld 1974)	SRP002750
3562	*Mb-1*	7	*nic-3*	< 4255303	1048	UV	M	(Weijer and Vigfusson 1972)	SRP002749
3564	*Mb-2*	1	*al-1*, *nit-1*	7377187 to 8029920	180	UV	M	(Weijer and Vigfusson 1972)	SRP002748
3566	*Mb-3*	1	*al-1*, *cen-1*	>3970000	1433	UV	M	(Weijer and Vigfusson 1972)	SRP004280
3831	*ff-1*	2	*un-20*, *aro-1*	2897659 to 3571946	172	Spontaneous	M	(Tan and Ho 1970)	SRP004330
3921	*tng*	2	*arg-5*, *pyr-4*	513651 to 1551100	201	Spontaneous	SL2	(Springer and Yanofsky 1989)	SRP002768
7022	*fld*	4	*∼arg-14*, *his-5*	< 2792033	827	Spontaneous	M	(Perkins *et al.* 1962)	SRP003536
7035	*Per-1*	5	*ilv-1*, *asn-1*	1620310 to 5558912	1033	UV	SL3	(Howe and Benson 1974)	SRP002701

a “SL” indicates the reference genome background (St. Lawrence), and the number indicates the number of backcrosses to a reference strain. “L” indicates the Lindegren background, and “M” indicates that there is a mixed background.

## Materials and Methods

*Neurospora* strains are described in Table 1 and are available from the Fungal Genetics Stock Center. Genomic DNA was purified from mycelia grown in Vogels liquid medium, using a simple phenol/chloroform extraction technique (Lee *et al.* 1988). Mycelia from mutants with limited vegetative growth was produced by macerating tissue in a glass tissue pulverizer under sterile conditions. DNA was randomly sheared into small fragments of between 200 and 300 bp in size using Covaris E210 according to the manufacturer's recommendation. The overhangs created by fragmentation were converted into blunt ends using T4 DNA polymerase and DNA polymerase I Klenow fragment. Using dATP, base 'A' is added to the 3′ end of the blunt phosphorylated DNA fragment to prepare the DNA for ligation to the adaptors. Adaptors were then ligated to the DNA fragment using DNA ligase so that they would hybridize on a flowcell. Finally, using DNA Phusion polymerase, PCR was performed to selectively enrich those DNA fragments that have adapter molecules on both ends, and to amplify the amount of DNA in the library. DNA was sequenced on Illumina genome analyzer II. Reads were aligned to the reference genome sequence (Galagan *et al.* 2003) and single nucleotide polymorphisms (SNP), insertions, and deletions were characterized using maq-0.7.1 (Li *et al.* 2008) and with BreakDancer (Chen *et al.* 2009). Default maq parameters were used for SNP calling and for filtering as described in the maq paper, (maq.pl SNPfilter -f cns.indelse -F cns.indelpe -d 3 -q 40 -Q 60 -w 5 -N 2), with a subsequent minimum map quality filter of 30 applied after the indel proximity filters. The minimum consensus quality (phred scaled likelihood of the consensus base being incorrectly called) and depth filters minimize the likelihood that random sequencing errors lead to false positive SNP calls. A direct estimate of false SNP identification was not determined, but is expected to increase as the divergence from the reference increases. Though this group of strains is not a freely reproducing population and the variants have not been confirmed to exist at an allele frequency to fit the definition of a polymorphism, all of the single nucleotide variants are referred to as Single Nucleotide Polymorphisms (SNPs) for simplicity. To allow comparisons between strains, each SNP or indel was assigned a unique identifier that included the contig and position. For example, a SNP would have an identifier such as “3_591470_C” indicating that it is on contig 3 at position 591,470 and that the base at that position is C. It was necessary to include the base in the SNP identifier, as multiple polymorphisms may occur at the same position. An indel would have an identifier such as “6_91954” indicating the contig and location; size information was not encoded for indels but direct comparisons among strains were directly possible because of the small number of unique indels in any region of the genome. For validation of the deletion in strain FGSC 3921, PCR was carried out with 100 ng genomic DNA using 0.5 uM primers and taq polymerase following manufacturer’s instructions. Primers for amplification of a 1.2 kb coding fragment of wild type NCU03436 were as follows: Forward- 5′-CGATACTCGCTTCGTCTTCC-3′, Reverse- 5′-ATCATCAAGTCCGCCACTTC-3′. Photographic microscopy of culture edge morphology was carried out using a glass microscope slide culture as previously described (Dieterle *et al.* 2010).

## Results

### Association of specific phenotypes with genes

#### tangerine:

**Figure 1:**
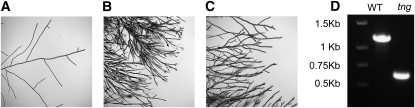
Morphological characterization of FGSC 3921 and validation of the deletion detected by whole genome sequencing. (A) FGSC 2489 (wt). (B) FGSC 3921 (*tng*) showing hyperbranching, swollen hyphae and cytoplasmic bleeding at the tips. (C) FGSC 16003 (NCU03436 [*stk-15*] KO) showing hyperbranching and swollen hyphae. (D) Agarose gel electrophoretic analysis of PCR products from wild type or a *tng* genomic DNA.

The morphological mutant tangerine (*tng*) causes the production of enlarged conidia that are delimited by a membrane, but no cell wall (Springer and Yanofsky 1989). Although the sequence of strain FGSC 3921 revealed neither unique nonsynonymous or nonsense mutations, nor frame breaking indels in the region flanked by markers in the 1 Mb region delimited by flanking markers *arg-5* and *pyr-4* (supporting information, Figure S1), analysis of the genome sequence of FGSC 3921 using BreakDancer (Chen *et al.* 2009) revealed a unique 572 base deletion in this region that occurs in NCU03436 (Table 2). PCR amplification of this gene from FGSC 3921 confirmed that the deletion was correctly identified in this strain (Figure 1). NCU03436 is the *Neurospora* ortholog of *cpp-1* (**c**ell-shape-**c**ontrol **p**rotein **p**hosphatase) in *Fusarium verticillioides* where studies indicate an involvement in control of cell shape and fumonisin production. The phenotype for the NCU3436 knockout strain is identical to that of strains containing the classically derived mutation in *tng* (Figure 1).

**Table 2:** Mutations identified by whole genome sequencing

FGSC no.	Gene	Putative ORF	Mutation	Annotation
106	*com*	NCU06508	G->A: P->L	*gpip-3*
305	*amyc*	NCU02689	−1:G Frameshift	*lrg-1*
309	*ti*	NCU10497	8 bp Deletion	Oligosaccharyl transferase
322	*ty-1*	NCU00455	2 bp Deletion	*ste50*
821	*ts*	NCU01459	−1:T Frameshift	*asl-2*
1211	*dot*	NCU00896	−1:A Frameshift	*Sac1* (yeast)
1303	*fi*	NCU04990	−1:A Frameshift	*stk-17*
1363	*smco-1*	NCU02762	8 bp insertion	*Cch1*/*trm-11*
2261	*do*	NCU06871	G->A:H->R	β-1,3 glucan synthase
3114	*Sk-2*	—	—	
3246	*fs-n*	NCU02794	+1:A Frameshift	*so*, *ham-2*
3562	*mb-1*	?		
3564	*mb-2*	NCU00652	W:TGG ->TAG Stop	Hypothetical
3566	*mb-3*	NCU00658	Premature Stop	Hypothetical
3831	*ff-1*	NCU01543	+1:C Frameshift	PTAB
3921	*tng*	NCU03436	572 base deletion	*stk-15*
7022	*fld*	NCU09739	−1:G Frameshift	*ada-7*
7035	*per-1*	NCU03584	−1:A Frameshift	*PKS-7*

#### fluffyoid:

Strains with a mutation in *fluffyoid* (*fld*) send up aerial hyphae that only very rarely conidiate under normal growth conditions. However, when *fld* mutants are grown under carbon limitation conditions, conidiation occurs at 25°C but not 34°C (Springer and Yanofsky 1989). Strain FGSC 7022 has over 78,000 SNPs and 8907 indels (Figure S2). Among 918 indels that are unique to strain FGSC 7022, one unique deletion was identified in the region near *arg-14* and to the left of *his-5* that causes a frameshift mutation in the annotated ORF NCU09739. This deletion is found in all reads covering this region in this strain and is the only frameshift inducing indel in NCU09739 among 18 strains (there is one instance of a 3 base deletion in this ORF in strain FGSC 821). There are no unique nonsynonymous or nonsense SNPs in strain 7022 left of the flanking marker *his-5* further supporting the conclusion that the variant in NCU09739 is responsible for the *fluffyoid* phenotype. NCU09739 encodes a protein predicted to be a Zn(II)2Cys6 type fungal transcription factor and is annotated as *all development altered-7* (*ada-7*) in The *Neurospora crassa* e-Compendium. Deletion of NCU09739 leads to defects in conidiation, hyphae and female (but not male) fertility(Dunlap *et al.* 2007).

#### compact:

Strains with mutant *com* make small colonies with restricted radial growth (Perkins and Ishitani 1959). Genetic mapping places the mutation associated with *com* between *ace-2* and *ad-4* on LGIII, a span of 741 kb containing only 44 ORFs on supercontig 3. Strain FGSC 106 has over 23,000 SNPs including 1,033 unique SNPs (Figure S3). There is one unique variant in FGSC 106 that occurs in the region delimited by the flanking markers and it occurs in NCU06508. This variant encodes a C629T (ccg to ctg) mutation at the DNA level and a Phenylalanine to Leucine change at amino acid residue 210 out of 1123 and this variant occurred in all 76 reads covering this position. NCU06508 is annotated as a glycosylphophatidylinositol anchor phosphoethanolamine transferase-3 (*gpip-3*) that is involved in production of glycosylphosphatidylinositol (GPI) anchors between proteins and the plasma membrane. RIP mutants in this gene were found to have defects in morphology at both the colony and microscopic levels of *gpip-3* (Bowman *et al.* 2006). These defects are very similar to those described for strains containing a mutant in *compact*.

#### amycelial:

Multiple phenotypes associated with the mutation *amyc* are small colony size including, increased cell wall thickness, and non-hyphal, multipolar budding growth under restrictive conditions. Conidiation is considered “conditional” and is dependent in part on carbon source. A single unique deletion was found in strain FGSC 305 among the 166 ORFs in the 742 kb region delimited by the flanking markers (Figure S4). The G at position 3,212,445 in gene NCU02689 is deleted resulting in a frameshift mutation and this deletion is detected in all reads covering this region in this strain. There are no other indels in this ORF among all the strains sequenced and a knockout of this gene has altered sexual morphology (FGSC #11464 Mating Type: a NCU02689.2 Heterokaryon (*lrg-1*)) (Colot *et al.* 2006). Additionally, a conditionally morphological mutant in this gene was identified and was named *lrg-1* (Seiler and Plamann 2003). It was subsequently shown that LRG1 is essential for hyphal tip extension and that it plays a role in regulating (through RHO1) β1,3-glucan synthase activity. A mutation in *lrg-1* was shown to increase activity of β1,3-glucan synthase as evidenced by decreased sensitivity to caspofungin; it is possible that the increased cell wall thickness of characteristic of *amyc* grown under restrictive conditions (Coniordos and Turian 1973) is due to increased β1,3-glucan synthase activity.

#### tiny:

Another morphological mutant, tiny (*ti*), shows temperature sensitivity and decreased colony size with increasing temperature. There are no nonsynonymous SNPs in strain 309 in the 730 kb region carrying the mutation tiny (*ti*) (Figure S5) and there is only one unique indel among the 163 ORFs in this region. This 8 base deletion causes a frameshift mutation in the coding sequence of NCU10497 beginning at amino acid 405 out of 750 total amino acids. This frameshift mutation introduces fourteen nonsense codons, beginning at amino acid 445. NCU10497 encodes the oligosaccharyl transferase STT3 subunit. Stt3 is an essential gene in both *Saccharomyces cerevisiae* and *Schizosaccharomyces pombe*. Similar to alleles in *S. cereviseae*, the tiny allele is temperature sensitive. Because stt3 is an essential component of the N-linked glycosylation pathway, it is logical to hypothesize that this causes the decrease in cell wall protein in the *ti* mutant. A gene deletion mutant of NCU10497 was nonviable as a homokaryon (Dunlap *et al.* 2007).

#### fissure:

Strain FGSC 1303 contains only one unique indel and no unique SNPs in the 1 Mb region between *pyr-1* and *ace-4* (Figure S6). The deletion is the only indel in the gene NCU04990 and it removes a single nucleotide in the coding sequence causing multiple nonsense codons. This deletion is found in all reads covering this region in this strain. NCU04990 is predicted to encode the ortholog of the *vhs/ran1* serine threonine kinase in *S. cereviseae*. The deletion of this gene in yeast leads to decreased resistance to hyperosmotic stress and accumulation of glycogen. An *N. crassa* knock-out mutant for this ORF has reduced asexual spore formation and abnormal sexual morphology (Dunlap *et al.* 2007).

#### semicolonial-1:

FGSC 1363 has one unique insertion of 8 bases among the 409 ORFs in the 2 Mb region flanked by mating type and *rg-1* (Figure S7). This indel occurs in NCU02762, an ORF that contains domains associated with calcium channels, and which is an ortholog of *CCH1* from *Saccharomyces cerevisiae*. In *S. cerevisiae*, *CCH1* mutants have impaired ability to take up Ca^2+^ (Peiter *et al.* 2005) in response to α mating factor as well as increased sensitivity to some stress conditions. A deletion of the *cch1* ortholog from *Fusarium graminearum* leads to a growth phenotype reminiscent of *smco-1* (Hallen and Trail 2008). A *Neurospora* gene deletion mutant of NCU02762 was only recovered as a heterokaryon suggesting that a homokaryotic deletion would be lethal. All of the SNPs in this region are either shared, or occur in ORFs that have polymorphisms in multiple strains.

#### dot:

The mutation known as *dot* is in a 1.7 Mb region on LG IR that contains 415 ORFs. The only unique variant in this region in strain FGSC 1211 is the deletion of one T from NCU00896 (Figure S8), which is detected in all reads covering this sequence in FGSC 1211. As with other morphological mutants, a gene deletion mutant of this ORF was only recovered as a heterokaryon, suggesting that it may be essential for growth or sexual reproduction. This ORF encodes a phosphatidylinositol phosphate (PtdInsP) phosphatase and is the ortholog of *SAC1* from *S. cerevisiae*. Mutations in this gene have been implicated in growth regulation in yeast, as well as in hereditary disease in humans (Manford *et al.* 2010). In yeast, sac1p is localized to the ER and plays an important role in actin cytoskeleton organization, cell wall synthesis, Golgi function, lipid metabolism and vacuole morphology through regulation of phosphatidylinositol 4-phosphate levels (Rivas *et al.* 1999).

#### doily:

The mutation *doily* (*do*) was deposited to the FGSC collection in 1972 and is characterized by colonial morphology that is sensitive to the carbon source in the culture medium. FGSC 2261 carries the only allele known of the mutation *doily* (*do*). While this strain has a relatively high number of polymorphisms (Table 3, Table 4, and Figure S9), most of the SNPs are not unique or occur in ORFs that have other polymorphisms in multiple strains. However, one unique nonsynonymous SNP (A->G at position 3,652,079) was found in the ORF designated NCU06871. This variant had the maximum possible quality score and changes a histidine to an arginine at position 1,246 of the predicted protein. NCU06871 encodes a glycoside transferase 48 family protein involved β-1,3 glucan synthesis. The ortholog of this gene in *Aspergillus fumigatus* has been shown to be essential. Similarly, strains of *Fusarium solani* in which RNAi has been used to decrease expression of its ortholog show defects in morphology (Beauvais *et al.* 2001; Ha *et al.* 2006).

**Table 3:** Types of single nucleotide polymorphisms among 18 strains of *Neurospora crassa*

Strain	Total	NC	Synonymous	Non Synonymous	3′	5′	Int	Nonsense	% NC	% Syn	% Non Synonymous	% Nonsense
106	23579	14110	4536	2269	851	494	1143	18	59.84	32.15	9.62	0.08
305	90195	49883	18740	8417	4875	2560	4709	67	55.31	37.57	9.33	0.07
309	13274	9949	1725	667	321	144	414	11	74.95	17.34	5.02	0.08
322	142489	88879	24733	11525	6692	3112	6413	95	62.38	27.83	8.09	0.07
821	188346	112490	35810	17193	8458	4177	8825	122	59.73	31.83	9.13	0.06
1211	20493	16100	2263	1146	339	187	380	19	78.56	14.06	5.59	0.09
1303	59356	41212	8773	4330	1828	816	2119	35	69.43	21.29	7.29	0.06
1363	146641	71259	35955	15999	8535	4463	8685	137	48.59	50.46	10.91	0.09
2261	44839	25265	9894	4507	1772	911	2111	37	56.35	39.16	10.05	0.08
3114	41085	24550	9176	3186	1461	751	1626	31	59.75	37.38	7.75	0.08
3246	21533	14301	3405	1556	839	404	843	14	66.41	23.81	7.23	0.07
3562	106533	57767	22686	10480	5800	2830	5862	78	54.22	39.27	9.84	0.07
3564	47981	31886	7821	3702	1661	777	1737	36	66.46	24.53	7.72	0.08
3566	37516	29134	3962	1875	806	354	1198	27	77.66	13.6	5	0.07
3831	22961	17290	2563	1369	523	253	792	15	75.3	14.82	5.96	0.07
3921	80311	47446	16023	7557	3158	1605	3901	84	59.08	33.77	9.41	0.1
7022	78991	51944	12585	5843	3110	1602	3270	45	65.76	24.23	7.4	0.06
7035	18487	13174	2656	1160	472	241	686	13	71.26	20.16	6.27	0.07

**Table 4:** Indel distribution among 18 strains of *Neurospora crassa*

Strain	Total Indels	Unique Indel	CDS Indels	NC Indels	Intron	Splice Site	Other
106	3607	136	294	2636	289	7	381
305	13335	816	1014	9492	1052	18	1759
309	1959	123	137	1466	151	3	202
322	15105	2001	701	11182	1236	22	1964
821	21224	6941	919	16066	1637	27	2575
1211	1494	100	103	1141	106	3	141
1303	6110	501	321	4746	413	7	623
1363	24952	5213	2091	17573	1997	31	3260
2261	7059	546	600	5143	584	6	726
3114	4211	3468	246	2971	369	6	619
3246	2310	89	124	1781	146	4	255
3562	13753	1920	741	10131	1119	19	1743
3564	4960	116	274	3747	387	8	544
3566	3714	471	199	2933	261	6	315
3831	2450	140	138	1922	168	4	218
3921	9743	979	581	7391	748	10	1013
7022	8907	918	471	6765	625	8	1038
7035	1869	93	134	1386	148	2	199

#### female fertile-1:

*female fertile-1* (*ff-1*) was originally isolated as a mutant displaying a defect in female fertility. It was later shown to be allelic with a spontaneous mutant (glycerol phosphate-1; *glp-1*) that was characterized by its ability to efficiently conidiate and grow on glycerol as a carbon source. This mutation lies in a 675 kb region of chromosome 2 that includes 172 ORFs and that has very few polymorphisms in strain FGSC 3831 (Figure S10). In this strain, NCU01543 has a unique insertion of 1 base that introduces a frameshift mutation causing multiple stop codons, the first of which is located at position 113 out of 762. The insertion was identified in all reads covering this region in strain FGSC 3831. An orthologous gene in *Magnaporthe grisea* has been characterized (Li *et al.* 2010); the gene, *LDB1*, is so named because it encodes a predicted LIM-binding-domain. LIM domains are involved in protein–protein interactions (Kadrmas and Beckerle 2004). The phenotypes associated with deletion of this gene in *M. grisea* are a lack of asexual and sexual spore production, easily-wettable colonies and an inability to develop appresoria.

#### female sterile-n:

Female sterile-n (*fs-n*) is on LG 1 far from *mat* (35%; 45%) and near T(4637) *al-1*, a region of 80 kb containing only 18 ORFs (Figure S11). This mutation was characterized in 1974 (Mylyk and Threlkeld 1974) and the original description of this mutant suggested that there could be two closely linked lesions responsible for the lack of female fertility in strains carrying this trait. The genome sequence of strain FGSC 3246 revealed a unique insertion of one A into NCU02794 at position 9,112,074 and this insertion was detected in all reads covering this region in this strain. While there are no other indels in strain FGSC 3246 in the 80 kb region delimited by the flanking markers there are a number of SNPs in this region, although most are shared among multiple strains. Although NCU02794, known variously as *ham-2* or *so* (Table 2), has multiple nonsynonymous SNPs in other strains, further examination of the vegetative of morphology of FGSC 3246 indicates that is has a “soft” like phenotype. The protein encoded by NCU02794 contains a WW domain and is involved in hyphal fusion and was localized to septal plugs (Fleissner *et al.* 2005). A knockout of this ORF is female sterile and has abnormal vegetative morphology (Dunlap *et al.* 2007).

#### tyrosinaseless-1:

The *tyrosinaseless-1* (*ty-1*) mutant phenotype was initially described as female infertile, “velvet” (short aerial hyphae) and tyrosinaseless (Horowitz *et al.* 1961). Further analysis showed that tyrosinase was inducible under some conditions. Strain FGSC 322 carries over 1,400 indels in the 1.5 Mb region genetically shown to carry the mutation *tyrosinaseless-1* (Figure S12). Of these, 69 are in coding sequence but only three are unique to this strain. Of these three, NCU00240 exhibits frameshift inducing indels in other strains, suggesting that it is not responsible for the tyrosinaseless phenotype. NCU00403 and NCU00455 each exhibit unique frameshift inducing indels and both deletions are strongly supported in the sequence data. While gene deletion mutants are available for both, the phenotype for the gene deletion of NCU00403 was characterized by the *Neurospora* Program Project and exhibited “normal” growth and sexual development, including pigmentation of perithecia and ascospores (Dunlap *et al.* 2007). Thus, the likely gene associated with the *ty-1* phenotype is NCU00455, which encodes the ortholog of *S. cerevisiae* Ste50, a scaffold protein that connects the mitogen-activated protein kinase (MAPK) cascade with cell cycle machinery. The MAP kinase signaling cascade regulates a significant number of processes in fungi including the regulation of tyrosinase (Park *et al.* 2008). There are three MAP kinase cascades in *Neurospora crassa* that overlap in function and are involved in many biological processes, including cell morphology, conidiation, mating, and osmotic stress response (Borkovich *et al.* 2004).

#### male barren-1:

The mutation known as *mb-1* is on LG VII, and the strain carrying this mutation, FGSC 3562, has 741 indels in coding sequences of which 71 are on super contig 7 (Figure S13). Four of the 71 indels are unique to strain FGSC 3562 and three introduce frameshift mutations. Two deletions occur in NCU02251 and both are deletions of one base (at 1,076,703 and 1,076,825). However, a homokaryotic gene deletion mutant of this strain is available suggesting that this ORF is not responsible for the phenotype in FGSC 3562. The second ORF carrying a unique frameshift mutation in strain FGSC 3562 is NCU06930, a hypothetical protein with only limited orthology among filamentous ascomycetes. This same ORF has multiple nonsynonymous SNPs in strain FGSC 821. Four additional annotated ORFs have non-unique indels with NCU11995 having thirteen distinct indels in strains 3562 and 821. Although strain 3562 has no unique nonsense codons on contig 7 it has 800 nonsynonymous SNPs on contig 7 of which 13 are unique to this strain. Of these, NCU11995 has multiple nonsynonymous SNPs. This ORF encodes a C6 zinc finger domain-containing protein. Additionally, NCU06102 has two unique nonsynonymous SNPs although a homokaryotic gene deletion mutant for this ORF is available. Other ORFs containing unique nonsynonymous SNPs are in ORFs that contain other nonsynonymous SNPs in other strains. Because of the ambiguity presented by these multiple polymorphisms, it is not possible to uniquely identify one as being responsible for the male barren phenotype in strain FGSC 3562.

#### male barren-2:

Although there are no indels or nonsynonymous SNPs in the region carrying the *male barren-2* trait in strain FGSC 3564 (Table 1 and Figure S14), there is one unique nonsense mutation in this region in strain FGSC 3564. The G to A substitution occurred in all reads covering this position and has a quality score of 111. This variant encodes a unique nonsense codon in NCU00652 that is the only unique variant in this ORF among all 18 strains. The nonsense SNP in strain FGSC 3564 occurs at amino acid 659 out of 1188. Only four other strains display any variants in this ORF and none of these SNPs are unique.

#### male barren-3:

The last of the male-barren strains in the current investigation is *mb-3*, which is on linkage group IR near *al-1* and *mb-2* (Table 1). There is one unique nonsense mutation in NCU00658 in strain FGSC 3566 (Figure S15) and it occurs with high confidence. The nonsense mutation occurs at amino acid 219 out of 4008. A gene deletion mutant for this ORF has normal morphology, but is male barren.

#### tan spore:

*tan spore* (*ts*) is found in strain FGSC 821, which was deposited into the FGSC collection in 1961, and is described as having been a spontaneous mutation in strain 4a, the so called Emerson lineage (Nakamura and Egashira 1961). The phenotype for *ts* is light colored and/or immature ascoscores that do not germinate. The *ts* mutation was used in studies that showed that the majority of *N. crassa* perithecia originate from fertilization by a single male spore each. Strain FGSC 821 has the greatest nucleotide divergence relative to the reference genome (Table 3, Table 4, and Figure S16) of all the strains characterized in the present study. Nevertheless, no unique SNPs, and only one unique indel, occur in coding sequence in the 2 Mb region flanked by the markers *inl* and *cen-V* (Table 1). The unique mutation is a deletion of one base leading to frameshift in NCU01459 (Table 2) and this deletion is detected in all reads covering this region in strain FGSC 821. NCU01459 is a BZIP transcription factor whose deletion leads to ascospore inviability. The deletion of NCU01459 leads to immature tan ascospores and was noted in (Colot *et al.* 2006) who named the gene *ascospore lethal-2* (*asl-2*).

#### perithecial-1:

Analysis of FGSC 7035 genome sequence led to the identification of a unique 1bp deletion in the gene coding region of NCU03584 as well as two ORFs carrying unique nonsynonymous SNPs among the 1,033 ORFs in the region flanked by the markers *ilv-1* and *asn-1* (Figure S17). While the deletion in NCU03584 is the only variant in this ORF, both of the nonsynonymous SNPs occur in ORFs that have multiple nonsynonymous SNPs or frameshift causing indels in other strains. To validate the identification of NCU03584, and the identification of unique variants by whole genome sequencing, NCU03584 from FGSC 7035 was sequenced manually and the deletion was identified by this approach as well (data not shown). The deletion in NCU03584 occurs at nucleotide 114 resulting in a stop codon at nucleotide 162 in a 7271 nucleotide ORF. NCU03584 is the likely ortholog of the polyketide synthetase responsible for perithecial pigmentation in the related fungus, *Sordaria macrospora* (Engh *et al.* 2007).

### Summary of results for combined strain set

Eighteen genomes were sequenced to an average depth ranging from 28 to nearly 108- fold coverage (Table 5). Sixteen strains were sequenced entirely with paired-end reads producing between 27 and 90 million paired end reads resulting in an average sequence depth ranging from 29X to 108X. Two strains, FGSC 3566 and FGSC 3831 had over 20 million single-end reads in addition to over sixty-nine million paired-end reads. While strain FGSC 7035 generated over 90 million reads, many of the reads were of low quality and only 52% were mapped to the genome. Thirteen strains were sequenced with an average read length of 35 bases while five strains were sequenced with an average read length of approximately 75 bases. There were no significant differences in the ability to map these reads to the genome for either approach and each genome was compared to the over 41 million bases of the reference genome. The portion of the genome that was aligned and compared to the reference genome ranged from 99% of the reference genome size in the most conserved strain to 81% for the most diverged strain. Since multi-alleleic calls are not expected in a haploid genome, 240 coding multi-alleleic sites and 1100 non-coding multi-alleleic sites were examined to determine what might have caused these calls. Out of 240 coding sites examined, 53% were caused by indels that were too large for maq to properly align the reads, 36% were in reads that had low mapping quality scores and/or were orphan pairs, suggesting that the read could have been misaligned, 9% appeared truly homozygous, and 2% could not be explained. For non-coding sites 4% could be explained by missed indels, 93% were in reads that had low mapping quality scores and/or were orphan pairs, suggesting that the read could have been misaligned, and 3% appeared truly homozygous. Preprocessed data from this project are available via GenBank (Table 1), or at http://genome.jgi-psf.org/Neucr1/Neucr1.home.html and tables with the processed data are available as supplemental data.

**Table 5:** Sequencing statistics

Strain	Average Depth	No. SE	% SE Mapped	No. PE	% PE Mapped	% PE Pairs	Read Length	% Ref. Covered	No. Homozygous SNPs	No. Multi-Allelic SNPs	No. Homozygous Indels
106	57.74	0	NA	38170318	84	97	75	97	23083	496	2974
305	107.95	0	NA	62988628	94	97	76	95	88337	1858	11242
309	73.5	0	NA	43519088	95	96	75	99	12793	481	1502
322	30.74	0	NA	39552812	94	95	35	95	118890	23599	13171
821	28.6	0	NA	36997202	93	94	35	93	163792	24554	18872
1211	29.85	0	NA	35860788	98	98	35	99	10322	10171	1214
1303	29.93	0	NA	36903172	97	96	35	98	42851	16505	5376
1363	33.71	0	NA	27162118	69	97	75	86	145722	919	21906
2261	73.42	0	NA	49455190	88	90	76	95	44123	716	6019
3114	28.82	0	NA	35889678	95	96	35	98	27169	13916	3783
3246	32.31	0	NA	39347694	99	95	35	99	17290	4243	1992
3562	32.22	0	NA	39097638	98	98	35	96	99284	7249	12009
3564	32.39	0	NA	39134336	98	97	35	98	33587	14394	4221
3566	83.31	27708365	97	69894872	98	96	36	99	23580	13936	3195
3831	80.55	22353786	92	72379318	99	97	36	99	12885	10076	2022
3921	31.19	0	NA	38460204	96	97	35	96	73150	7161	8564
7022	47.17	0	NA	55991650	97	96	36	97	61278	17713	7634
7035	34.23	0	NA	90007810	52	86	35	99	13310	5177	1557

SE, single end reads; PE, paired end reads.

Because most of the target mutations were flanked by genetic markers that are already identified to the level of the gene sequence, the amount of the genome that require examination for each strain ranged from a low of 74 kilobases (kb) in strain FGSC 305 to over 4 megabases (Mb) in strain FGSC 3562 (Table 1). The average was just over 1.7 Mb, although for seven strains the amount of genome space that needed to be searched was less than 1 Mb. The number of annotated open reading frames provides another measure of the amount of genome space that must be searched to identify the mutation responsible for the relevant phenotype. For the strains in the current project this number ranged from a low of 18 ORFs in the 80 kb between flanking markers in strain FGSC 3246 to 1,433 ORFs in the 5.8 Mb to the right of *cen-1* in strain FGSC 3566 (Table 1).

**Figure 2:**
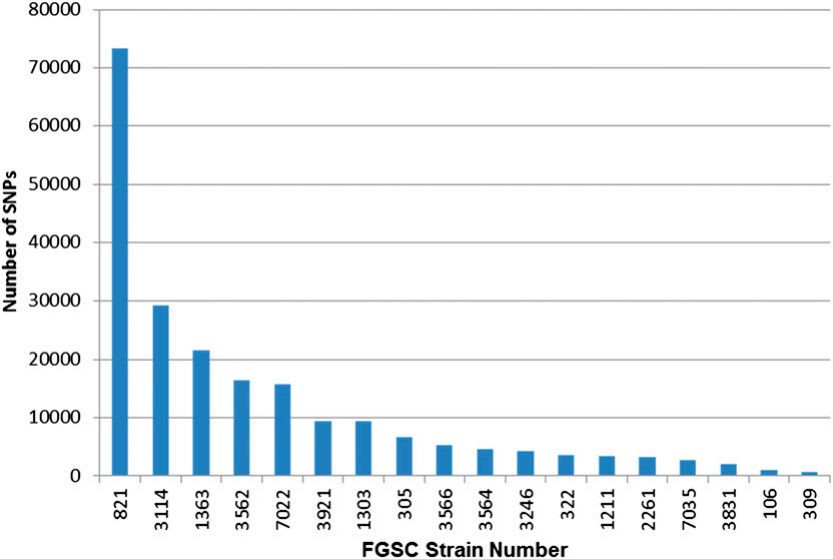
Unique single nucleotide polymorphisms (SNPs) in 18 strains of *Neurospora crassa*. The number of unique SNPs (Table 8) was plotted for each strain and strains were sorted based on the number of unique SNPs. For this analysis, SNPs that occurred more than once in the dataset were not included.

In the 18 genomes, there are 1,184,610 identified single nucleotide variants (Table 4). Of these, 1,137,606 (96.03%) map to contigs 1-7 (Table 6). Among these, there are 527,620 distinct positions with identified variants in all 18 strains. 244,043 of these variants occur only in one strain. 96 variants are identified as occurring in all 18 genomes, suggesting that the actual polymorphism belongs to the reference genome (Table 7). The distribution of variants among the eighteen strains varies from a high of over 188,346 to a low of 13,274 (Table 3), while the number of unique SNPs ranged from a high of 73,336 to a low of 704 (Figure 2 and Table 8). Overall, there were nearly three times more transition mutations than transversions (Table S1 in File S1). The distribution of SNPs in the 100 kb region directly flanking each mutation was calculated, separating shared and unique SNPs, and there was no apparent association between either whether the mutation was spontaneous or induced, or which mutagen was employed (Table S2 in File S1), suggesting that strain lineage and back-crossing are more important than the mutagen in influencing variant distribution. Remarkably, there are only 413 ORFs, out of nearly 10,000, that have no polymorphisms among the 18 strains (Table S3 in File S1). Among these are 274 conserved hypothetical proteins, hypothetical, or predicted proteins, but also several ribosomal protein genes, histone genes, a mating type gene, and the *frequency* gene that is central to the cell's circadian rhythm.

**Table 6:** Distribution of SNPs among the supercontigs

Strain	Contig 1	Contig 2	Contig 3	Contig 4	Contig 5	Contig 6	Contig 7	Contigs > 7
106	5044	947	3607	2250	853	391	9186	1301
305	38046	2537	8514	1019	26797	857	8075	4350
309	4943	2055	891	592	896	454	604	2839
322	29658	7256	28243	12754	24790	16428	22415	946
821	45205	12122	16891	41336	15980	30781	19517	6514
1211	4379	2207	4267	1635	1094	1608	3633	1670
1303	7227	13847	10817	4606	5237	12333	3341	1947
1363	43626	21549	19525	8625	32371	3402	17307	236
2261	1304	789	10200	3545	261	10966	15258	2516
3114	2725	2165	27516	1868	1614	1710	1731	1756
3246	7811	845	792	2606	6415	482	547	2035
3562	9178	4064	19381	28334	20810	13947	8024	2795
3564	5492	3142	1510	4691	16787	12046	1828	2485
3566	8701	5056	1402	9660	4716	1698	1570	4713
3831	2410	1584	6176	1187	2824	1177	5243	2360
3921	16641	2307	17416	9018	14521	5537	12038	2833
7022	32843	3897	1749	3105	11562	11989	10164	3682
7035	6040	1396	3145	3668	675	668	869	2026

**Table 7:** Frequency of individual SNPs among eighteen strains of *Neurospora crassa*

No. Occurrences	No. SNPs
1	227988
2	115667
3	68186
4	40033
5	24418
6	13026
7	5285
8	3817
9	2912
10	769
11	559
12	68
13	49
14	33
15	43
16	48
17	42
18	96

**Table 8:** Number of unique SNPs in each strain

Strain	Unique SNPs
821	73336
3114	29263
1363	21607
3562	16432
7022	15721
3921	9433
1303	9355
305	6653
3566	5306
3564	4612
3246	4253
322	3496
1211	3468
2261	3227
7035	2714
3831	1962
106	1033
309	704

There are 62,952 distinct indels (compared to the reference genome) present in the 18 strains analyzed (Table 4 and Table 9). Of these indels, 25,894 occur in only one strain (Table 9). Because many indels are present in multiple strains, the total number of indels mapped to the genomes is 146,762. Strains exhibit different numbers of indels (Table 4) ranging from a low of 1,494 in strain FGSC 1211 to a high of 24,952 in strain FGSC 1363. Thirteen strains have fewer than 10,000 indels and nine strains have fewer than 5,000 indels. Most strains have ten times more non coding indels than coding indels (Table 4). Indels affecting the splice junction are a small fraction of all indels, ranging from 1 to 3% of coding sequence indels in each strain. Of the distinct indels, 16,682 are repeated twice and 8,818 are repeated three times (Table 9). Relatively few distinct coding sequence indels are found more than ten times and there are more distinct indels that are present in all 18 strains than are in ten to seventeen strains combined, suggesting that the indels that are found in all 18 strains actually represent indels present in the reference genome relative to a putative consensus genome. 1,352 annotated ORFs have one indel and 495 contain two indels (Table 10). 198 ORFs have three indels and 96 ORFs have four indels. Fewer than 100 ORFs in total have more than five indels.

**Table 9:** Indels repeated among multiple strains

No. Indels	Present in How Many strains	No. CDS
25894	1	1885
16682	2	1168
8818	3	481
4968	4	284
3235	5	167
1586	6	69
640	7	39
453	8	25
347	9	17
108	10	2
56	11	4
25	12	0
22	13	0
14	14	1
15	15	1
13	16	1
19	17	2
57	18	15

**Table 10:** Number of indels per ORF among eighteen strains of *Neurospora crassa*

No. ORFs	Number of Indels
1352	1
495	2
198	3
96	4
55	5
31	6
19	7
9	8
3	9
4	10
3	11
0	12
4	13
1	23

**Figure 3:**
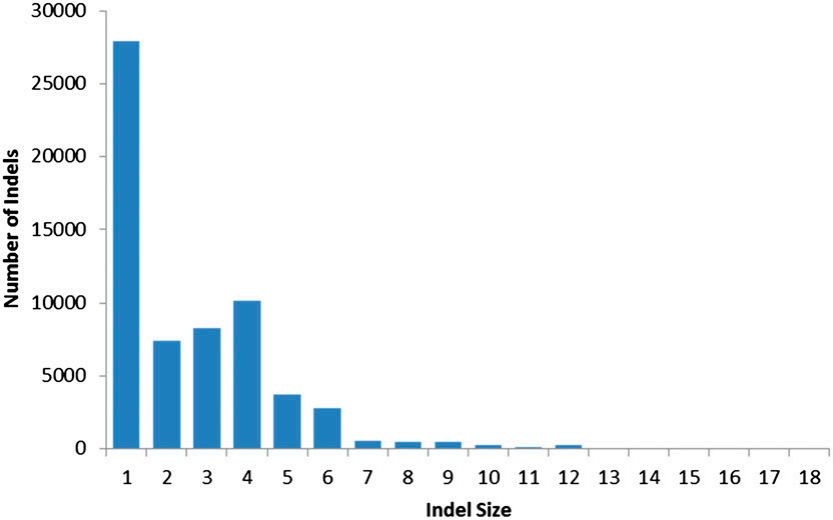
Distribution of unique indel sizes among eighteen strains of *Neurospora crassa*. The number of indels (Y axis) is plotted *vs.* the size of the indels (X axis). Insertions and deletions are pooled to give a single value for each size (Table S4 in File S1). Indels of size four are strongly overrepresented. Indels that are a multiple of three are also overrepresented. While indels may occur in multiple strains, each indel was only counted once to generate this dataset.

**Figure 4:**
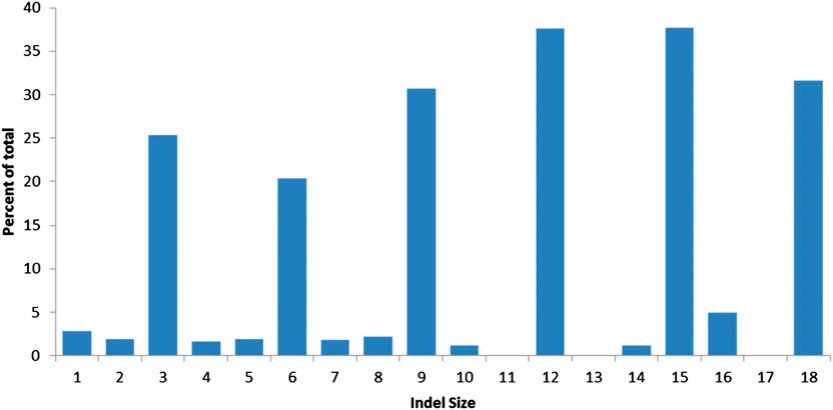
Indel size frequency in coding sequence among eighteen strains of *Neurospora crassa*. The total number of indels of a given size that occur in coding sequence was compared to the total number of indels of the same size and the resulting fraction was multiplied by 100 (Table S5 in File S1).

The distribution of indel sizes was found to be biased, both with regard to size and distribution in the genome. Indels of size +4 or −4 nucleotides were more common than would be predicted (Figure 3) and are dominated indels of a few specific sequences. Though neither is overrepresented in the genome, TACC and TAGG, often tandemly repeated, comprise 30% of the tetrameric indels. Indels that are +/− 3 or are a multiple of 3 nucleotides are up to ten times more common among coding sequence than in non-coding regions (Figure 4). Over 24% of all indels of three nucleotides occur in protein coding sequence, whereas less than two percent of indels of two or four nucleotides occur in protein coding sequence. The size of indel that is identifiable by the maq software is dependent upon the length of the read, with insertions being harder to identify. To determine the effect of read length on the ability to identify small indels, we altered the reference of supercontig 1 at evenly spaced intervals to simulate indels, aligned un-altered data to the altered reference, and then determined if maq was able to correctly identify the simulated indels. In general insertions are harder to identify than deletions, and longer reads allow more indels to be identified (Figure S18). BreakDancer (Chen *et al.* 2009) was used to identify larger events. It is known that this program will report false positive calls, especially for genomes that are repetitive, thus validation of predicted structural variation was necessary, as for FGSC 3921, above, although *Neurospora* has relatively low levels of repeated sequence (Galagan *et al.* 2003).

**Figure 5:**
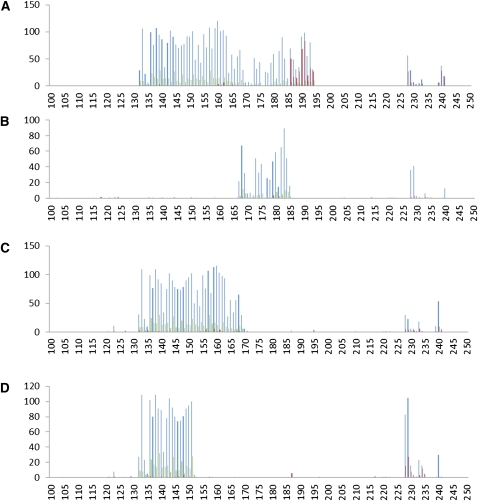
Putative mating type haplotype region. (A) FGSC 3246, (B) FGSC 7035, (C) FGSC 3562, (D) FGSC 3566. Distribution of SNPs and indels on the left arm of supercontig 1 in representative strains. The region from the left telomere through 2.5 million bases is shown. Total SNPs are plotted in blue. SNPs that are unique to each strain are plotted in red. Indels are plotted in green. The total number in a 10 Kb moving window is plotted on the Y axis. The X axis corresponds to the position along the contig. Strain 3246 is *mat a* while strains 7035, 3562, and 3566 are *mat A*.

Finally, the distribution of all polymorphisms is not uniform and demonstrates that different regions of each chromosome are inherited as blocks, or haplotypes (Figure S1, Figure S2, Figure S3, Figure S4, Figure S5, Figure S6, Figure S7, Figure S8, Figure S9, Figure S10, Figure S11, Figure S12, Figure S13, Figure S14, Figure S15, Figure S16, Figure S17, and Figure S19). It is suggested that such a block occurs around the mating type locus (Pomraning *et al.* 2011; Wik *et al.* 2008), which is on supercontig 1 at approximately 1,850,000. While most strains have either uniformly low or uniformly high divergence from the reference genome (*e.g.*, strains 106 or 821 in Figure S3 and Figure S16, respectively) in this region, strains that show abrupt changes from high divergence from the reference genome to low divergence from the reference genome in this region delimit a sub-region from position 1,840,000 on the left (Strain 7035) to position 2,280,000 on the right (in multiple strains), which has no shifts in the level of divergence from the reference genome (Figure 5). This sub-region contains 116 annotated ORFs (Colot *et al.* 2006) and includes 440 kilobases of DNA sequence. Immediately adjacent to the right end of this sub-region (from 2,280,000 to 2,410,000) is another sub-region with a high number of unique SNPs (in 15 strains). This sub-region contains 1,677 unique SNPs and 2,721 shared SNPs. Of these, 4,015 SNPs are non-coding, 123 are synonymous, and only 80 are nonsynonymous. The density of unique SNPs in this region is 13 per kb, as compared to the overall unique SNP density, which ranges from 0.02 per kb (in strain FGSC 309) to 1.8 per kb (in strain 821). The *mat a* region is defined by high a region of high SNPs from 1,320,000 to 1,940,000 in strain FGSC 3246 (the only *mat a* strain, shown in Figure 5A).

### Nonsense mutations

Four hundred and five distinct nonsense mutations were detected in the current data set for a total of 884 nonsense codons. The number of nonsense mutations varies among strains from a low of 11 in strain FGSC 309 to a high of 137 in strain FGSC 1363 (Table 11). Of these 405 different nonsense mutations, 188 occur once each. Nonsense SNPs occur in 337 different NCUs of which 175 occur in ORFs that have been deleted by the functional genomics program. While many of the ORFs carrying nonsense mutations are hypothetical or putative genes, fifteen of the genes carrying nonsense mutations have annotations in the *Neurospora* gene compendium, including genes such as *sad-2* (NCU04294; (Shiu *et al.* 2006)), or *so* (*ham-1*, NCU02794; (Fleissner *et al.* 2005)). Only four strains exhibit any variants in *sad-2*, including two strains, FGSC 821 and FGSC 3921, which feature a nonsense mutation that causes a stop codon at amino acid position 208 out of 1098. This same ORF has no indels in any strains analyzed in the current work.

**Table 11:** Number of nonsense mutations among eighteen strains of *Neurospora crassa*

Strain	Nonsense SNP
106	18
305	67
309	11
322	95
821	122
1211	19
1303	35
1363	137
2261	37
3114	31
3246	14
3562	78
3564	36
3566	27
3831	15
3921	84
7022	45
7035	13

Four strains carry nonsense mutations at amino acid position 21 in NCU09544 (*pod-2*; (Seiler and Plamann 2003)). Two of these, strains, FGSC 305 and FGSC 1363, have morphological phenotypes, while a third putatively carries a mutation in a regulatory protein, and the last is a male barren mutant. While there are multiple alleles of NCU09544 present among these four strains, all alleles carry the same nonsense mutation and vary in the number SNPs and the sizes of indels present. NCU09544 has been deleted by the *Neurospora* Functional Genomics program and the resulting mutant (FGSC 12737) has no phenotype (Dunlap *et al.* 2007) suggesting that the temperature sensitive *pod-2* allele previously isolated (Seiler and Plamann 2003) is actually a defective interfering mutation (Fujimura *et al.* 1993).

Multiple nonsense mutations in single ORFs led us to identify two genes that appear to have been RIPed (Freitag *et al.* 2002), NCU09968 and NCU09969. Neither gene is well conserved across sequenced fungi, although there does appear to be an expansion of putatively NCU09969 related genes in *Chaetomium globosum*. These two ORFs are adjacent on supercontig 6 and the co-occurrence of polymorphisms in around these two ORFs suggest that they are co-inherited. Neither have been deleted by the functional genomics program. Of the 405 nonsense mutations, 313 (77%) occur in ORFs annotated as conserved hypothetical proteins, 21 as ”hypothetical protein” and 24 as “predicted protein.” There are 9,734 total ORFs in the dataset, and 6,481 (48%) are conserved hypothetical proteins. Comparing the ratio of nonsense mutations among conserved hypothetical proteins to the ratio of conserved hypothetical proteins among all proteins shows that conserved hypothetical proteins are overrepresented among the ORFs carrying nonsense mutations by nearly 30%.

### Introgressed region in strain FGSC 3114

**Figure 6:**
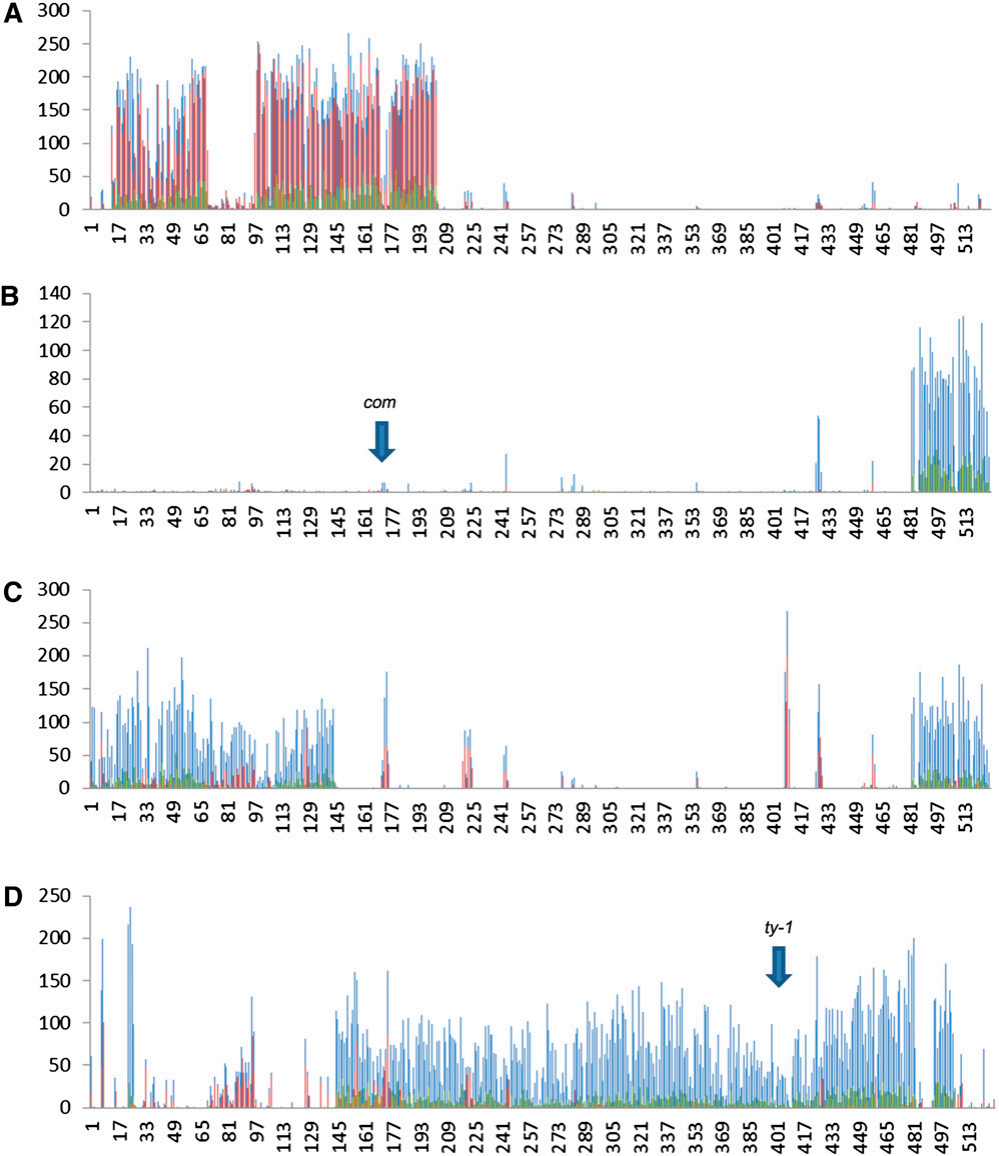
Distribution of polymorphisms on supercontig 3. (A) Strain FGSC 3114 containing the introgressed Sk-2 region. (B) Strain FGSC 106 with a vertical arrow showing the location of *com*. (C) Strain FGSC 305. (D) Strain FGSC 1363. Total SNPs are plotted in blue. Unique SNPs are plotted in red. Indels are plotted in green. Polymorphisms were sorted by supercontig and position and the total number in a 10 Kb moving window is plotted on the Y axis. The X axis corresponds to the position along the supercontig (X 10 Kb).

The genome sequence of the strain carrying the introgressed meiotic drive element, Sk-2, has relatively low divergence from the reference genome in terms of SNPs (Table 3) and indels (Table 4). The variants are predominantly seen on supercontig 3, consistent with the genetic identification of the meiotic drive element (Table 6 and Figure S19). The genome sequence data clearly shows that the introgressed region does not include the left telomere on contig 3, but does span the centromere (Figure 6). The number of SNPs on supercontig 3 ranges from a low of 792 in strain FGSC 3246 to a high of 28,243 in strain 322 (Table 6). This variability is not evenly distributed with strains FGSC 322 and FGSC 1363 sharing most SNPs while other strains, such as FGSC 106 and FGSC 305 share SNPs only on the right end. While there are large numbers of SNPs and indels in the introgressed region, in the absence of a complete sequence for *N. intermedia*, their identity or distribution do not provide significant insight into the nature of the Sk-2 element.

### Spontaneous mutation rate

The ability to sample genome sequence of strains that have been highly back-crossed provides an estimate of the upper limit of the rate of spontaneous mutations in sexual reproduction (µ) (Baer *et al.* 2007). To generate this estimate, the number of SNPs unique to each strain was calculated (Table 8). The distribution of unique SNPs among strains follows a generally Poisson distribution (Figure 2). Strains 106 and 309 were deposited into the FGSC collection in 1960 and had been backcrossed into the reference genome three times. These strains have the lowest number of unique SNPs and hence provide the best estimate for the maximum value of the background mutation rate in the organism. The total sequenced genome size in the current dataset is 41,061,603, and strain FGSC 309 has 704 unique SNPs, which translates to a mutation rate of 1.71 × 10^−5^, whereas strain FGSC 106 has 1,033 unique SNPs, which translates to a mutation rate of 2.5 × 10^−5^. These values are two orders of magnitude lower than the rate suggested in (Drake *et al.* 1998) who reported a rate of 0.003 for *N. crassa*. Adding in the unique indels does not significantly change this calculation. Strain FGSC 309 has 123 unique indels and adding these to the 704 unique SNPs gives an estimate for the maximum value of µ = 2 × 10^−5^. While some of the difference in value could be explained by false base calls in our data set, it is doubtful that the false call rate would account for the magnitude of this difference.

## Discussion

Our sequencing and analysis has led to the putative identification of sixteen of the seventeen mutations in the strains sequenced, and delimited an introgressed region in the eighteenth strain. Because the sequence of each strain can be evaluated in the context of the sequence of eighteen other strains, the ability to evaluate causative sequence polymorphisms was markedly enhanced. Moreover, since each mutant strain has a defined phenotype that segregates in a limited region of the genome, any sequence polymorphisms that are outside that defined region could be excluded from the specific analysis to identify the lesion responsible for the phenotype. Within the genetically defined region, each sequence polymorphism was evaluated relative to the eighteen other sequences. If a polymorphism occurred in a strain that did not display the mutant phenotype, it was presumed that the sequence polymorphism is not responsible for the phenotype. This procedure constituted, is in one manner of speaking, an *in silico* bulk analysis. The ability to recover the same mutation by manual sequencing, as in FGSC 7035 or by PCR in FGSC 3921 (Figure 1), provided technical validation of the whole genome sequencing approach.

Additionally, these evaluations of putative mutations were strengthened by data from the *Neurospora* Functional Genomics program (Dunlap *et al.* 2007), which has generated knockout strains for 7,669 genes (at the time of writing). Of these 7,699, 1,271 are only available as heterokaryons. These heterokaryons were not able to be purified by crossing to a wild type strains, suggesting that the deleted ORFs are essential, or at least essential for sexual reproduction. The availability of these mutants as viable homokaryons or as heterokaryons facilitates evaluation of whether the polymorphisms identified by whole genome sequencing would likely be responsible for the phenotypes seen in the mutants sequenced in the current project. Five mutations were associated with knock-out strains exhibiting the same phenotype and seven mutations exhibit similar phenotypes to the phenotypes of classical mutants in the same gene in *Neurospora*, or in related organisms. While complementing each mutation is outside the scope of the current study, the ability to associate an otherwise anonymous sequence variant with characterized mutations in *Neurospora* or related organisms validates the approach taken in the current project.

The distribution of sequence divergence among these eighteen strains is highly related to the strain histories. Strains that are closely related to the reference genome strain have the lowest sequence divergence relative to the reference genome, while those that were generated in a different background and not crossed into the reference genome background have the highest sequence divergence. For example, strain FGSC 821 is in the Emerson lineage and this strain has the highest sequence divergence from the reference of all the strains. Strains FGSC 106 and FGSC 309 were both deposited into the FGSC collection in 1960, but both bore mutations that had been induced in the reference genome background, and both were backcrossed three times into the reference genome background. Both of these strains have very low sequence divergence from the reference. However, the expectation that the mutations would be found in small regions of relatively high divergence was not supported. Instead, each strain has small regions of high divergence that have the appearance of blocks of DNA that has been inherited together, or haplotypes (Figure S1, Figure S2, Figure S3, Figure S4, Figure S5, Figure S6, Figure S7, Figure S8, Figure S9, Figure S10, Figure S11, Figure S12, Figure S13, Figure S14, Figure S15, Figure S16, and Figure S17). The characterization of a region surrounding the mating type locus provides a starting point for additional analysis of haplotype distribution among these strains.

The distribution of indels, and especially those whose size is a multiple of three, was highly biased in coding regions (Figure 4). This distribution is similar to the distribution seen in the human genome (Messer and Arndt 2007). Although the present analysis does not include a characterization of the nature of indels, beyond their size and location, the abundance of tetrameric indels was unexpected.

Nonsense mutations were identified in a surprisingly large number of exons, which, when considered in the context of the large number of numerous nonsynonymous SNPs, emphasizes the observation that most genes can be deleted with no visible phenotype (Dunlap *et al.* 2007). The number of nonsense mutations and frameshift inducing insertions and deletions suggest that most of the classical mutant strains carry significant cryptic second-site mutations. Thus careful evaluation of historical functional analysis of classical mutations is warranted. Furthermore, the value of backcrossing a strain into a well characterized genetic background is strongly validated.

Our analysis of genomic polymorphisms among eighteen strains of *Neurospora crassa* has led to identification of the nature of mutations associated with historically described phenotypes. It has also associated some of these classical mutants with other strains that have been characterized both in terms of phenotype and genotype. The ability to evaluate these polymorphisms in the context of classical genetic analysis as well as in the context of a functional genomics program has leveraged the whole genome analysis. The value of the *in silico* bulk sequence analysis is demonstrated by the facility with which polymorphisms were associated with phenotypes. The added value of the whole genome resequencing has included a characterization of SNP distribution, the identification of unexpected nonsense mutations, and has revealed the strong bias in the size of insertions and deletions relative to their location in coding *vs.* non-coding sequence. While genome resequencing is becoming more accessible, the ability to find meaningful information in this data, especially when combined with both classical and modern genetic data, is demonstrated by the present approach.

## Supplementary Material

Supporting Information

Corrigendum
