# Supplementary material for: Rediscovery by Whole Genome Sequencing: Classical Mutations and Genome Polymorphisms in Neurospora crassa
Source: G3 (Bethesda). 2011 Sep 1;1(4):303–16. doi: 10.1534/g3.111.000307 (PMC3276140; doi:10.1534/g3.111.000307)
Supplement: Supporting Information [file supp_1_4_303__index.html]

Supporting Information 

# Rediscovery by Whole Genome Sequencing: Classical Mutations and Genome Polymorphisms in *Neurospora crassa*

## Supporting Information for McCluskey *et al.*, 2011

**Files in this Data Supplement:**

- Supporting Information - Figures S1-S19 and File S1 (PDF, 2.3 MB)
- Figure S1 - Distribution of polymorphisms in strain 3921 (PDF, 124 KB)
- Figure S2 - Distribution of polymorphisms in strain 7022 (PDF, 132 KB)
- Figure S3 - Distribution of polymorphisms in strain 106 (PDF, 104 KB)
- Figure S4 - Distribution of polymorphisms in strain 305 (PDF, 128 KB)
- Figure S5 - Distribution of polymorphisms in strain 309 (PDF, 108 KB)
- Figure S6 - Distribution of polymorphisms in strain 1303 (PDF, 112 KB)
- Figure S7 - Distribution of polymorphisms in strain 1363 (PDF, 140 KB)
- Figure S8 - Distribution of polymorphisms in strain 1211 (PDF, 116 KB)
- Figure S9 - Distribution of polymorphisms in strain 2261 (PDF, 108 KB)
- Figure S10 - Distribution of polymorphisms in strain 3831 (PDF, 104 KB)
- Figure S11 - Distribution of polymorphisms in strain 3246 (PDF, 100 KB)
- Figure S12 - Distribution of polymorphisms in strain 322 (PDF, 152 KB)
- Figure S13 - Distribution of polymorphisms in strain 3562 (PDF, 116 KB)
- Figure S14 - Distribution of polymorphisms in strain 3564 (PDF, 112 KB)
- Figure S15 - Distribution of polymorphisms in strain 3566 (PDF, 116 KB)
- Figure S16 - Distribution of polymorphisms in strain 821 (PDF, 160 KB)
- Figure S17 - Distribution of polymorphisms in strain 7035 (PDF, 100 KB)
- Figure S18 - Detection of simulated insertions and deletions in whole genome sequence of Neurospora (PDF, 56 KB)
- Figure S19 - Distribution of polymorphisms in strain 3114 (PDF, 96 KB)
- File S1 - Supporting Data (.zip, 11.5 MB)
