# Supplementary material for: Rediscovery by Whole Genome Sequencing: Classical Mutations and Genome Polymorphisms in Neurospora crassa
Source: G3 (Bethesda). 2011 Sep 1;1(4):303–16. doi: 10.1534/g3.111.000307 (PMC3276140; doi:10.1534/g3.111.000307)
Supplement: Supporting Information [file supp_1.4.303_000307.pdf]

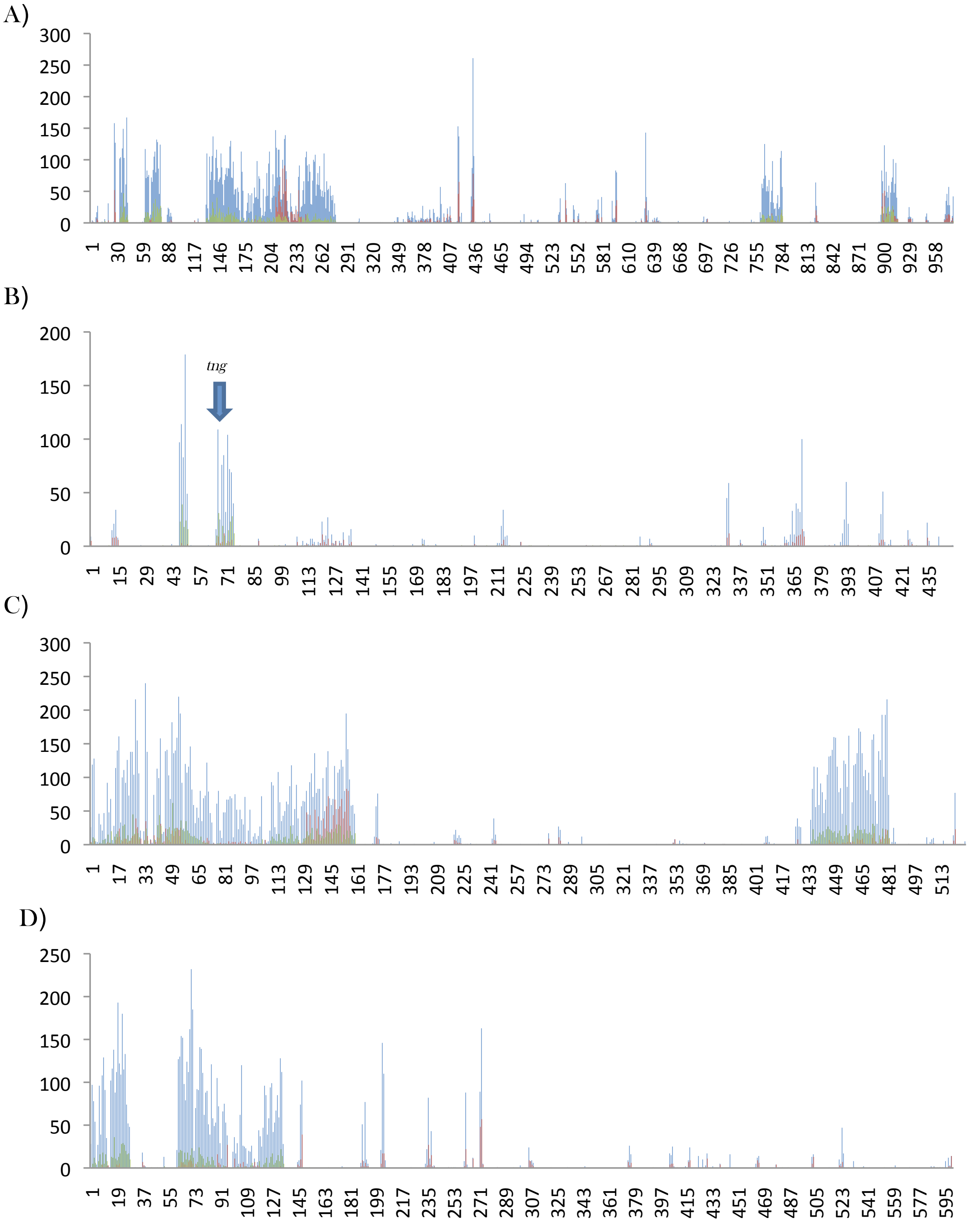
A)
300
250
200
150
100
50
0
1 30 59 88 117 146 175 204 233 262 291 320 349 378 407 436 465 494 523 552 581 610 639 668 697 726 755 784 813 842 871 900 929 958
B)
200
150
100
50
0
tng
1 15 29 43 57 71 85 99 113 127 141 155 169 183 197 211 225 239 253 267 281 295 309 323 337 351 365 379 393 407 421 435
C)
300
250
200
150
100
50
0
1 17 33 49 65 81 97 113 129 145 161 177 193 209 225 241 257 273 289 305 321 337 353 369 385 401 417 433 449 465 481 497 513
D)
250
200
150
100
50
0
1 19 37 55 73 91 109 127 145 163 181 199 217 235 253 271 289 307 325 343 361 379 397 415 433 451 469 487 505 523 541 559 577 595

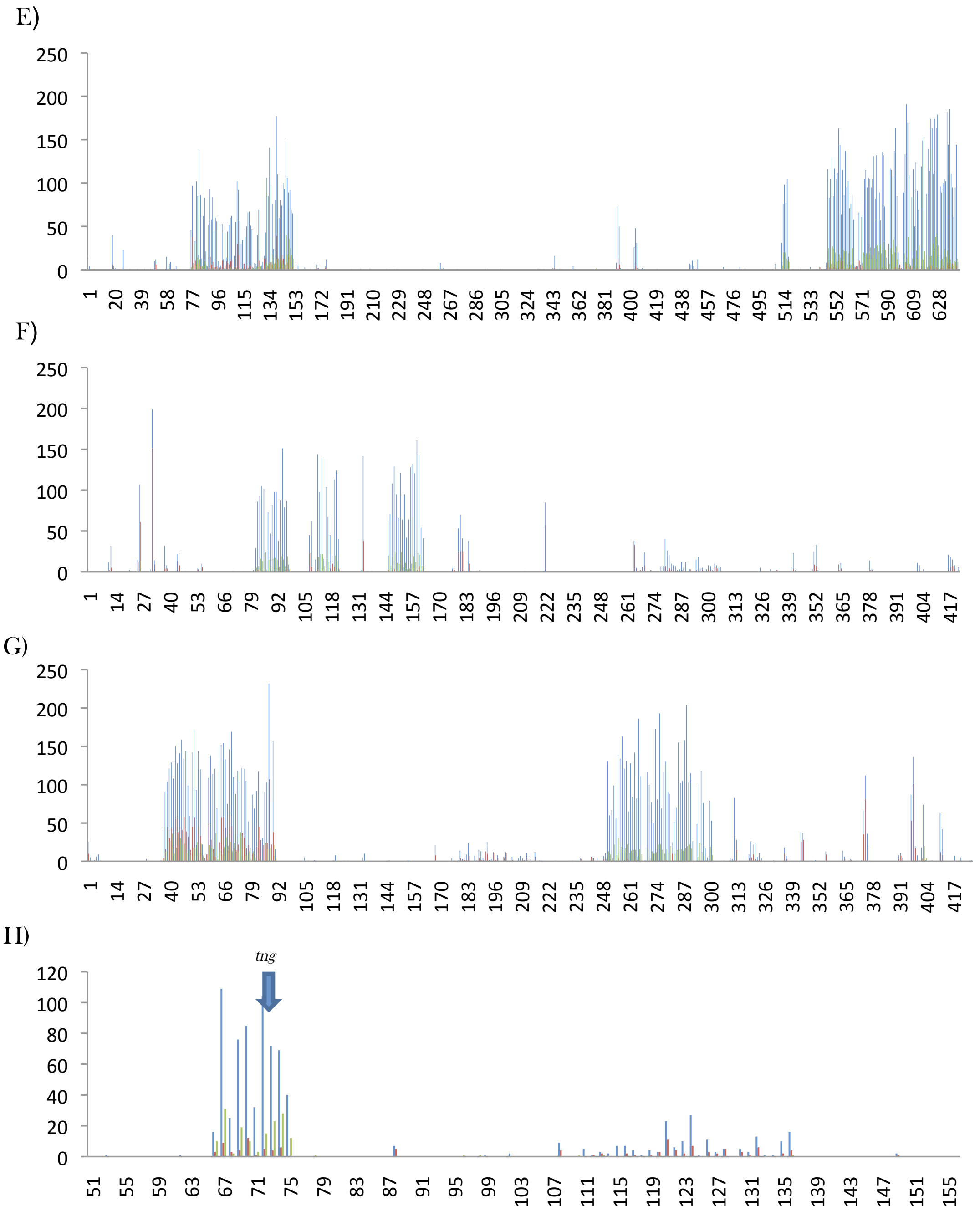

Figure S1 Distribution of polymorphisms in strain 3921. A) Supercontig 1, B) Supercontig 2. The location of *tng* is indicated by a vertical arrow, C) Supercontig 3, D) Supercontig 4, E) Supercontig 5, F) Supercontig 6, G) Supercontig 7, H) The region from 513651 to 1551100 on Supercontig 2. The location of *tng* is indicated by a vertical arrow. Total SNPs are plotted in blue. SNPs that are unique to strain 3921 are plotted in red. Indels are plotted in green. Polymorphisms were sorted by Supercontig and position and the total number in a 10 kb moving window is plotted on the Y axis. The X axis corresponds to the position along the Supercontig.

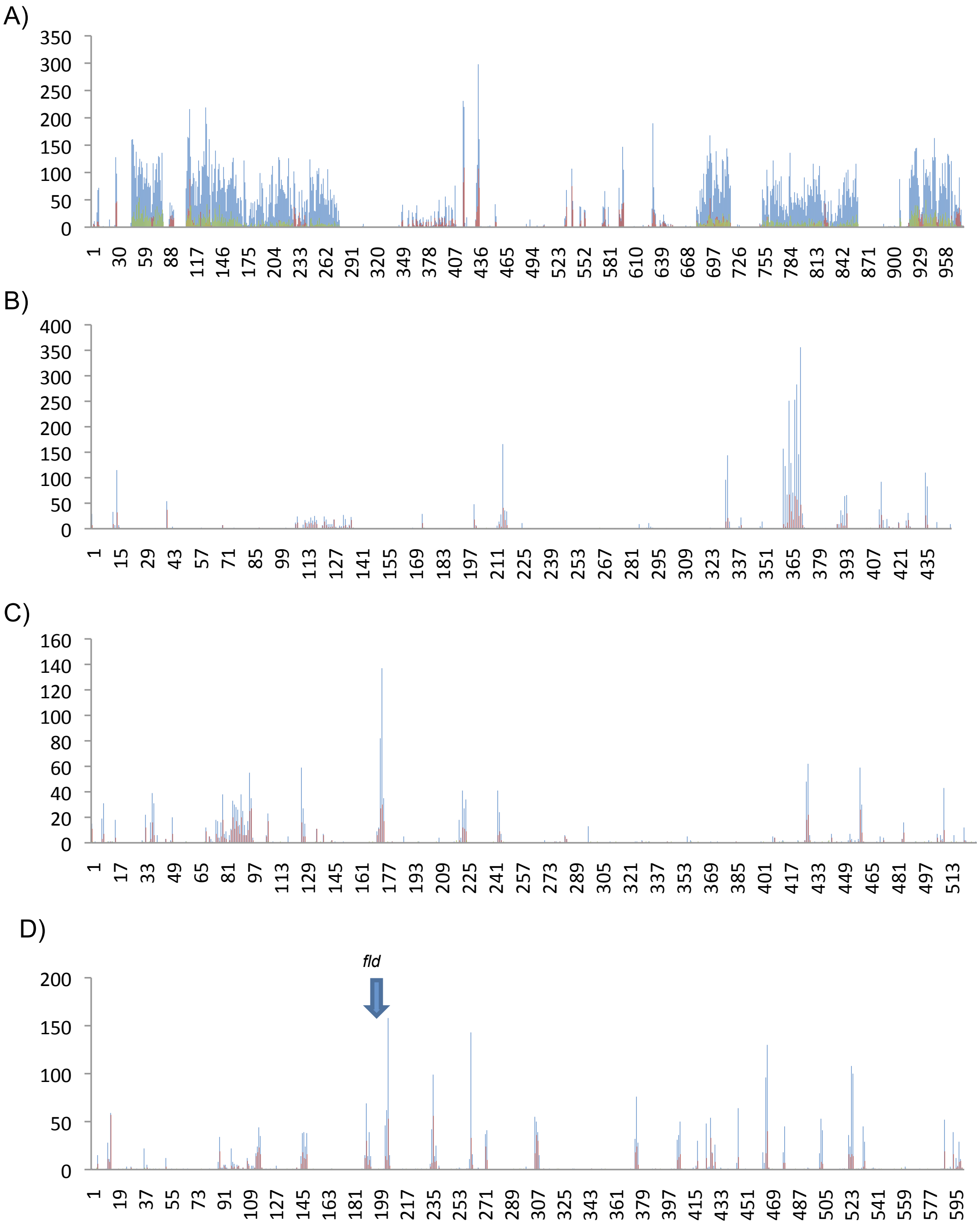
A)
B)
C)
D)
fld

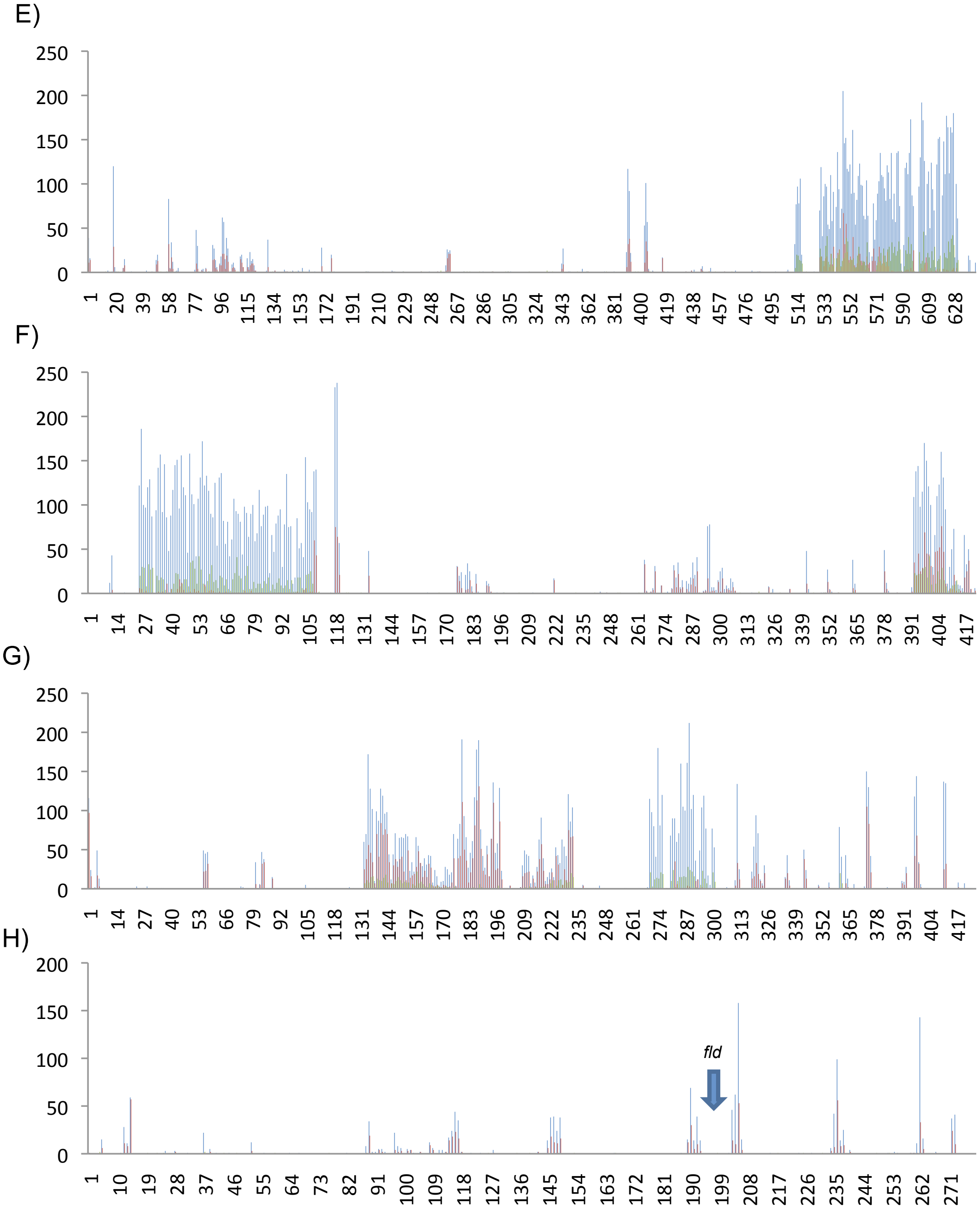

Figure S2 Distribution of polymorphisms in strain 7022. A) Supercontig 1, B) Supercontig 2, C) Supercontig 3, D) Supercontig 4. The location of *fld* is indicated by a vertical arrow, E) Supercontig 5, F) Supercontig 6, G) Supercontig 7, H) The region to the left of *his-5* on Supercontig 4. The location of *fld* is shown by a vertical arrow. Total SNPs are plotted in blue. SNPs that are unique to strain 7022 are plotted in red. Indels are plotted in green. Polymorphisms were sorted by Supercontig and position and the total number in a 10 kb moving window is plotted on the Y axis. The X axis corresponds to the position along the Supercontig (X 10 kb).

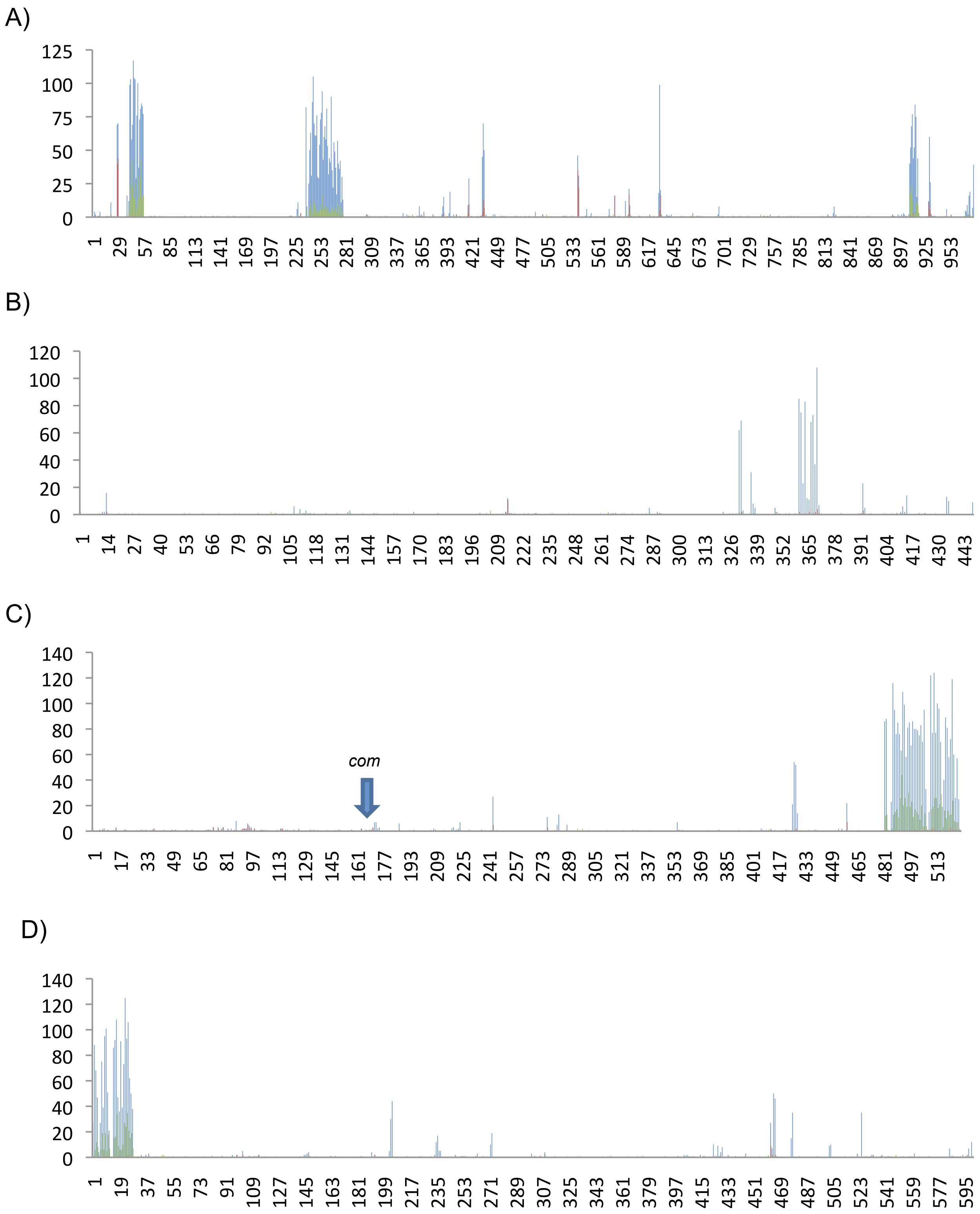
A)
125
100
75
50
25
0
1 29 57 85 113 141 169 197 225 253 281 309 337 365 393 421 449 477 505 533 561 589 617 645 673 701 729 757 785 813 841 869 897 925 953
B)
120
100
80
60
40
20
0
1 14 27 40 53 66 79 92 105 118 131 144 157 170 183 196 209 222 235 248 261 274 287 300 313 326 339 352 365 378 391 404 417 430 443
C)
140
120
100
80
60
40
20
0
com
1 17 33 49 65 81 97 113 129 145 161 177 193 209 225 241 257 273 289 305 321 337 353 369 385 401 417 433 449 465 481 497 513
D)
140
120
100
80
60
40
20
0
1 19 37 55 73 91 109 127 145 163 181 199 217 235 253 271 289 307 325 343 361 379 397 415 433 451 469 487 505 523 541 559 577 595

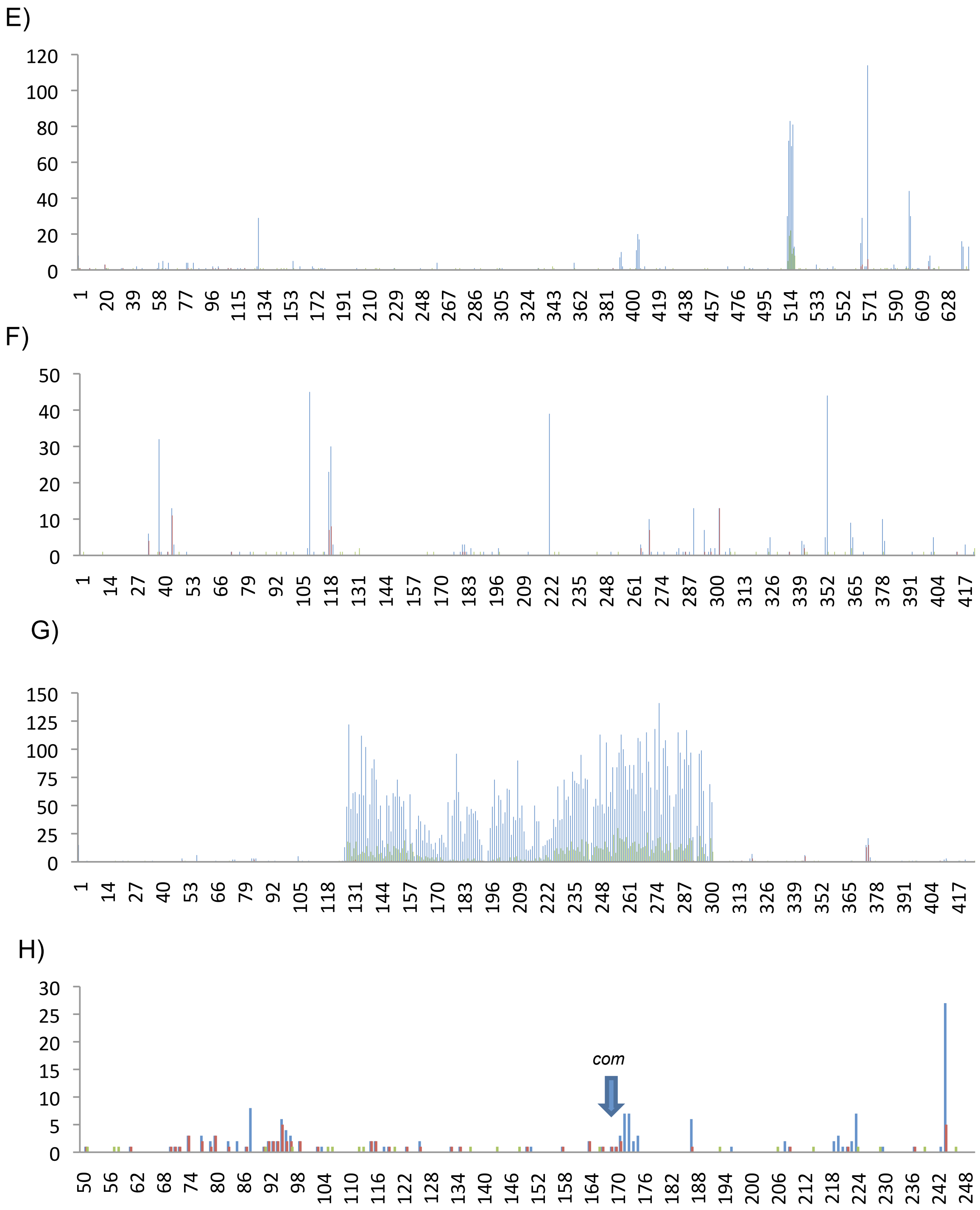

Figure S3 Distribution of polymorphisms in strain 106. A) Supercontig 1, B) Supercontig 2, C) Supercontig 3. The location of *com* (1680135) is indicted by a vertical arrow. D) Supercontig 4, E) Supercontig 5, F) Supercontig 6, G) Supercontig 7. H) The region defined by flanking markers on Supercontig 3 The location of *com* (1680135) is indicted by a vertical arrow. Total SNPs are plotted in blue. SNPs that are unique to strain 106 are plotted in red. Indels are plotted in green. Polymorphisms were sorted by Supercontig and position and the total number in a 10 kb moving window is plotted on the Y axis. The X axis corresponds to the position along the Supercontig. Numbers are X 10 kb

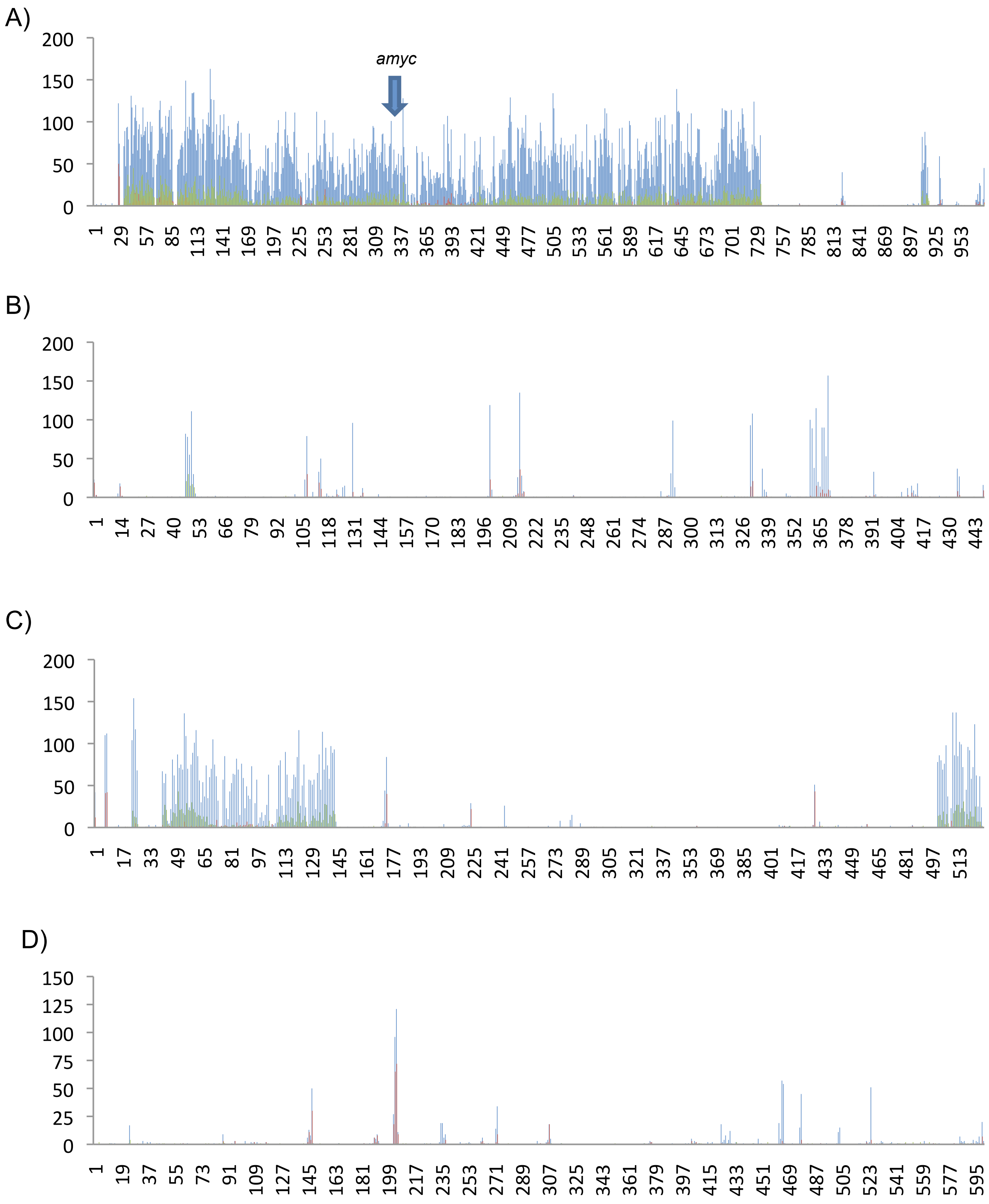
A)
amyc
200
150
100
50
0
1 29 57 85 113 141 169 197 225 253 281 309 337 365 393 421 449 477 505 533 561 589 617 645 673 701 729 757 785 813 841 869 897 925 953
B)
200
150
100
50
0
1 14 27 40 53 66 79 92 105 118 131 144 157 170 183 196 209 222 235 248 261 274 287 300 313 326 339 352 365 378 391 404 417 430 443
C)
200
150
100
50
0
1 17 33 49 65 81 97 113 129 145 161 177 193 209 225 241 257 273 289 305 321 337 353 369 385 401 417 433 449 465 481 497 513
D)
150
125
100
75
50
25
0
1 19 37 55 73 91 109 127 145 163 181 199 217 235 253 271 289 307 325 343 361 379 397 415 433 451 469 487 505 523 541 559 577 595

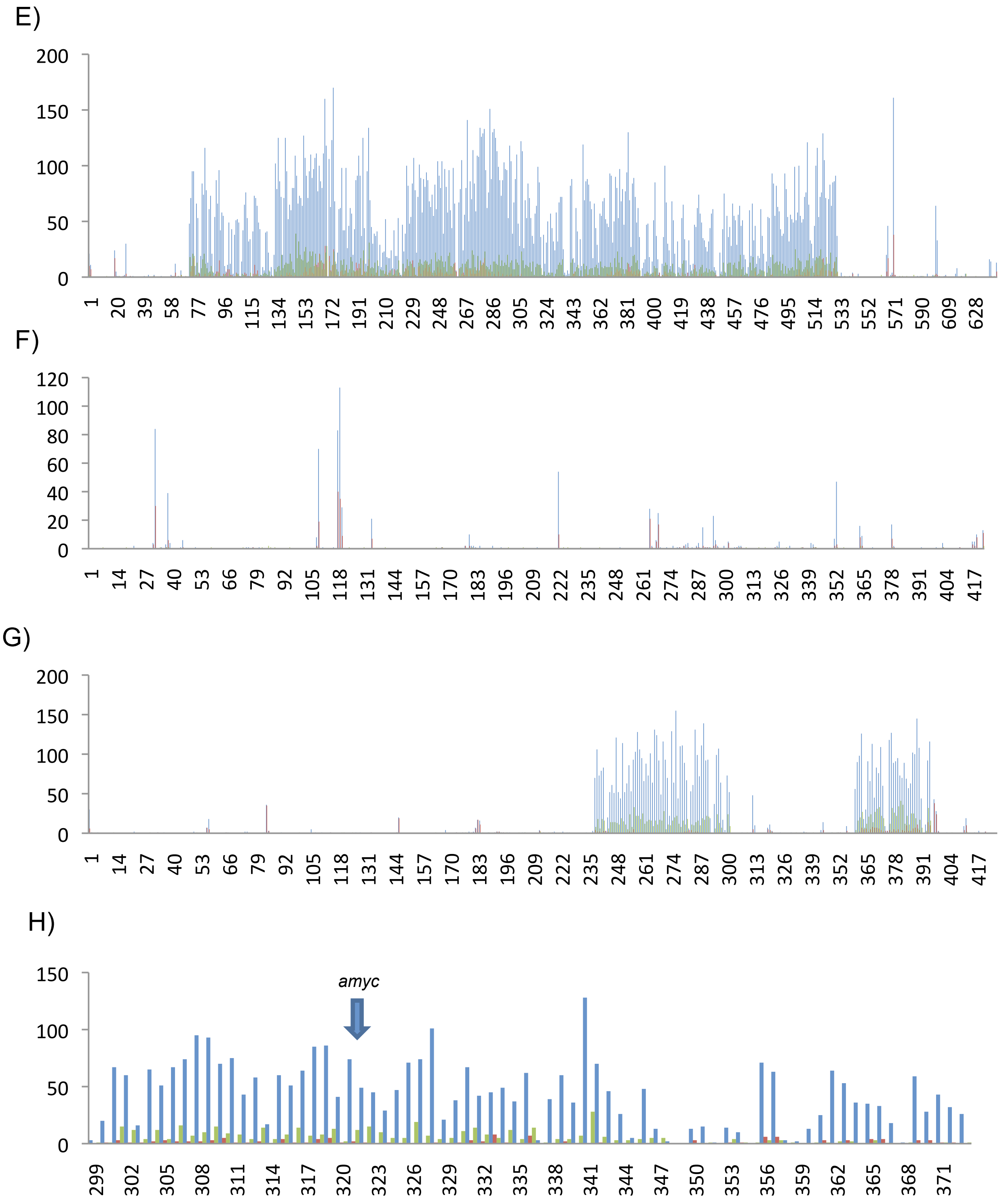

Figure S4 Distribution of polymorphisms in strain 305. A) Supercontig 1. The location of amyc is indicated by a vertical arrow, B) Supercontig 2, C) Supercontig 3, D) Supercontig 4, E) Supercontig 5, F) Supercontig 6, G) Supercontig 7. H) The region from 2988766 to 3730000 on Supercontig 1. The location of *amyc* is indicated by a vertical arrow. Total SNPs are plotted in blue. SNPs that are unique to strain 305 are plotted in red. Indels are plotted in green. Polymorphisms were sorted by Supercontig and position and the total number in a 10 kb moving window is plotted on the Y axis. The X axis corresponds to the position along the Supercontig. Distances are X 10 kb

A)

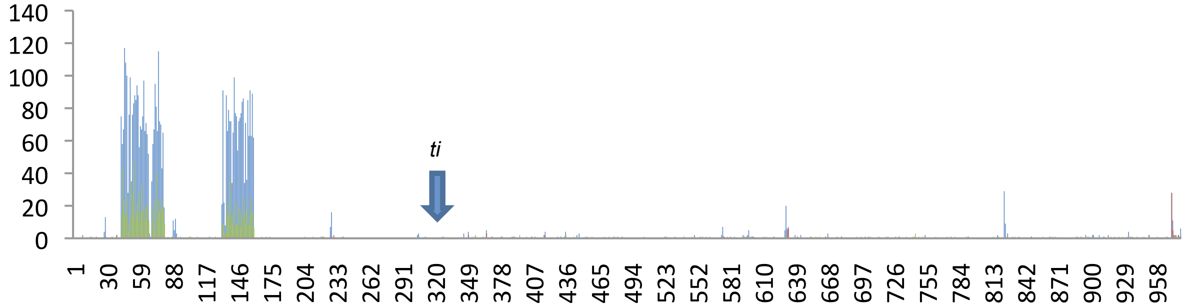
ti
140
120
100
80
60
40
20
0
1 30 59 88 117 146 175 204 233 262 291 320 349 378 407 436 465 494 523 552 581 610 639 668 697 726 755 784 813 842 871 900 929 958

B)

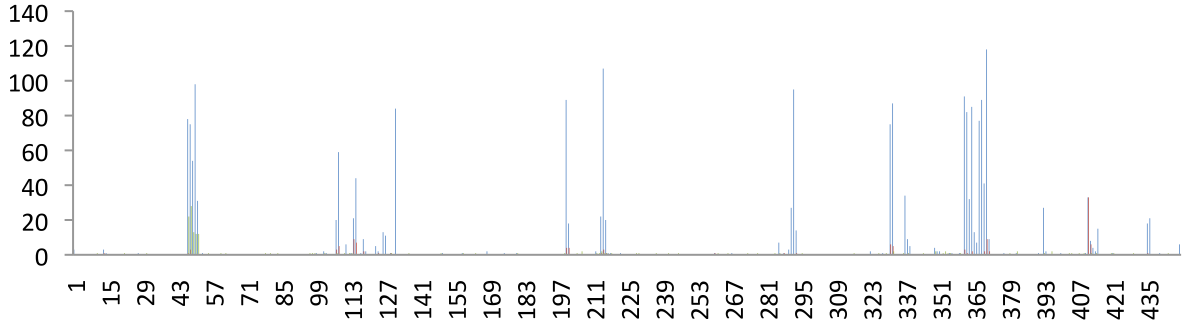
140
120
100
80
60
40
20
0
1 15 29 43 57 71 85 99 113 127 141 155 169 183 197 211 225 239 253 267 281 295 309 323 337 351 365 379 393 407 421 435

C)

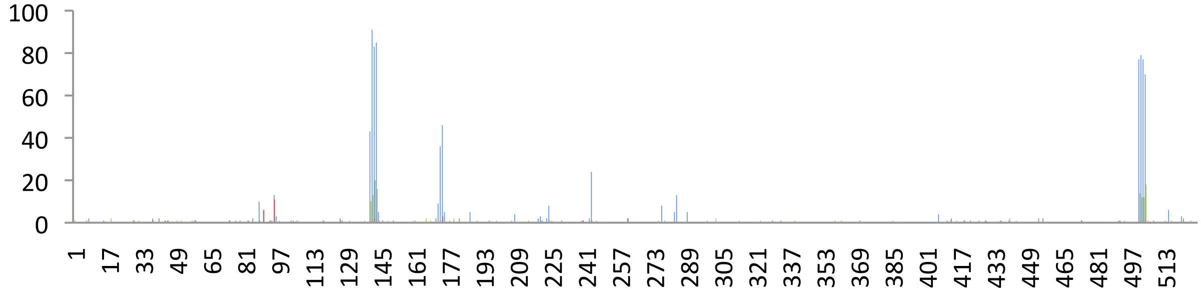
100
80
60
40
20
0
1 17 33 49 65 81 97 113 129 145 161 177 193 209 225 241 257 273 289 305 321 337 353 369 385 401 417 433 449 465 481 497 513

D)

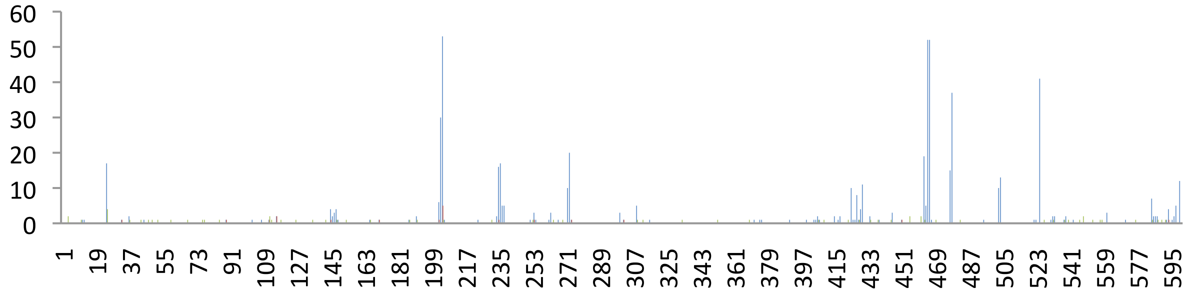
60
50
40
30
20
10
0
1 19 37 55 73 91 109 127 145 163 181 199 217 235 253 271 289 307 325 343 361 379 397 415 433 451 469 487 505 523 541 559 577 595

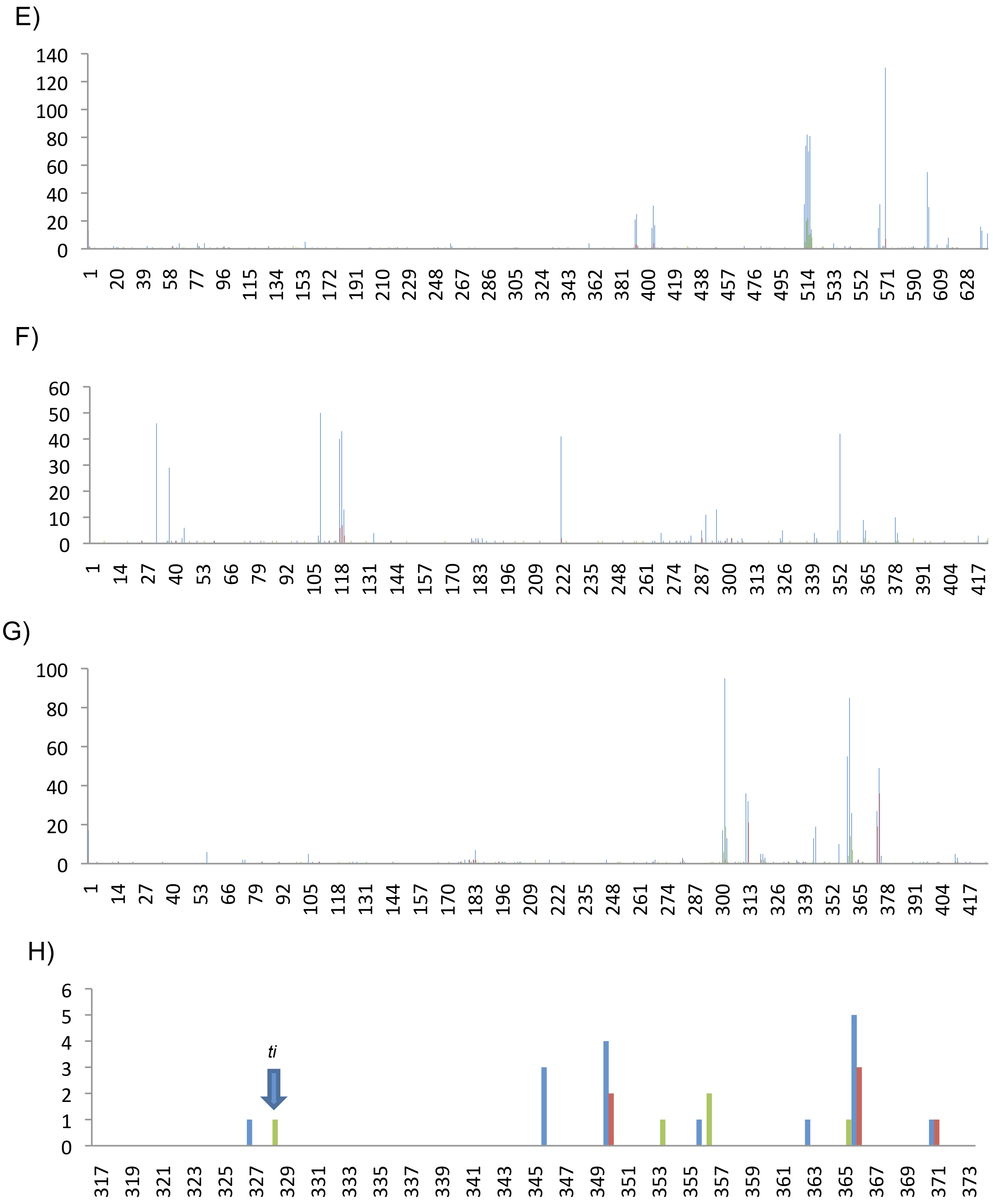

Figure S5 Distribution of polymorphisms in strain 309. A) Supercontig 1. The location of *ti* is indicated by a vertical arrow, B) Supercontig 2, C) Supercontig 3, D) Supercontig 4, E) Supercontig 5, F) Supercontig 6, G) Supercontig 7. H) The region from 3170432 to 3730000 on Supercontig 1. The location of *ti* is indicated by a vertical arrow Total SNPs are plotted in blue. SNPs that are unique to strain 309 are plotted in red. Indels are plotted in green. Polymorphisms were sorted by Supercontig and position and the total number in a 10 kb moving window is plotted on the Y axis. The X axis corresponds to the position along the Supercontig. Distances are X 10 kb.

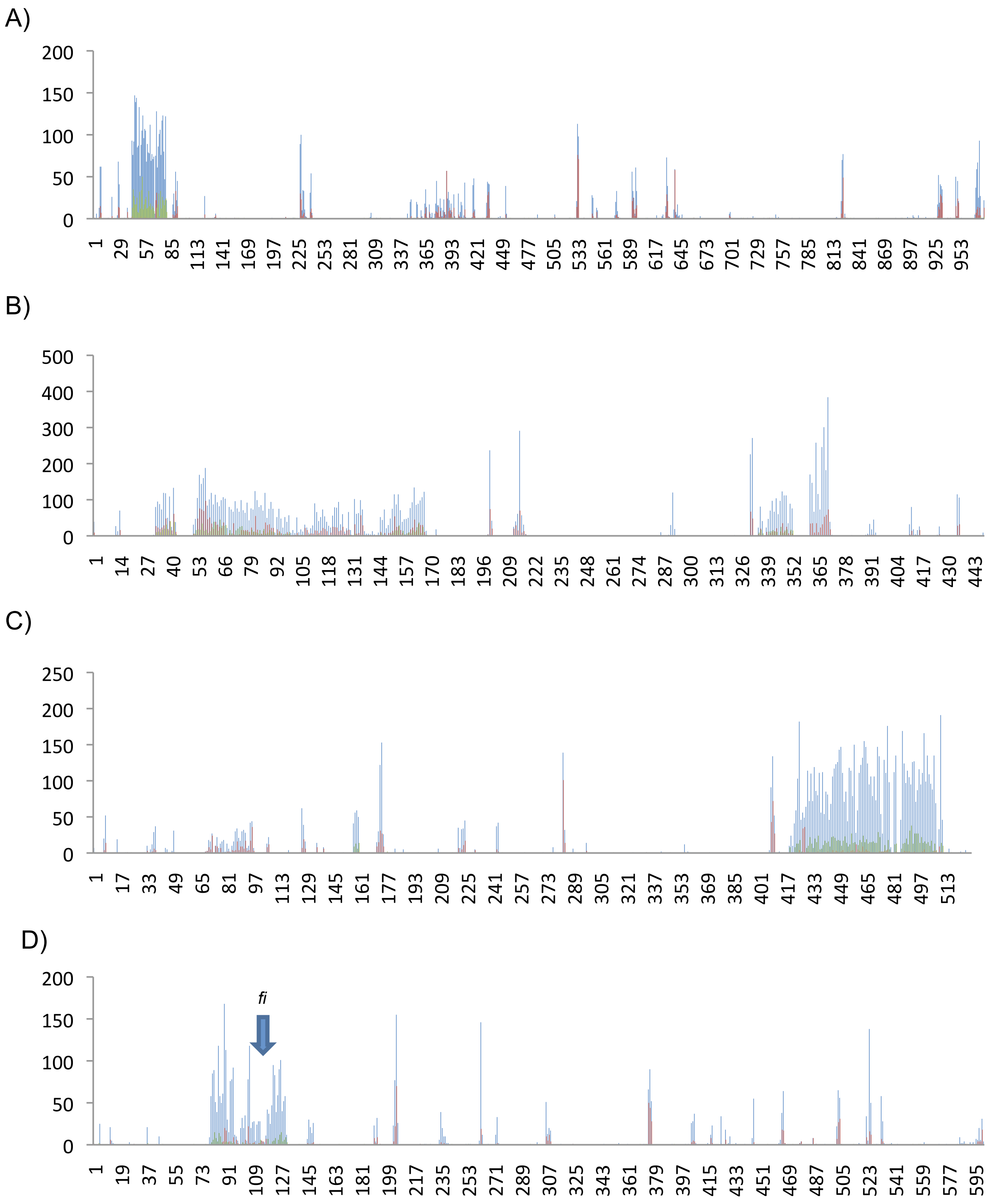
A)
200
150
100
50
0
1 29 57 85 113 141 169 197 225 253 281 309 337 365 393 421 449 477 505 533 561 589 617 645 673 701 729 757 785 813 841 869 897 925 953
B)
500
400
300
200
100
0
1 14 27 40 53 66 79 92 105 118 131 144 157 170 183 196 209 222 235 248 261 274 287 300 313 326 339 352 365 378 391 404 417 430 443
C)
250
200
150
100
50
0
1 17 33 49 65 81 97 113 129 145 161 177 193 209 225 241 257 273 289 305 321 337 353 369 385 401 417 433 449 465 481 497 513
D)
200
150
100
50
0
fi
1 19 37 55 73 91 109 127 145 163 181 199 217 235 253 271 289 307 325 343 361 379 397 415 433 451 469 487 505 523 541 559 577 595

E)

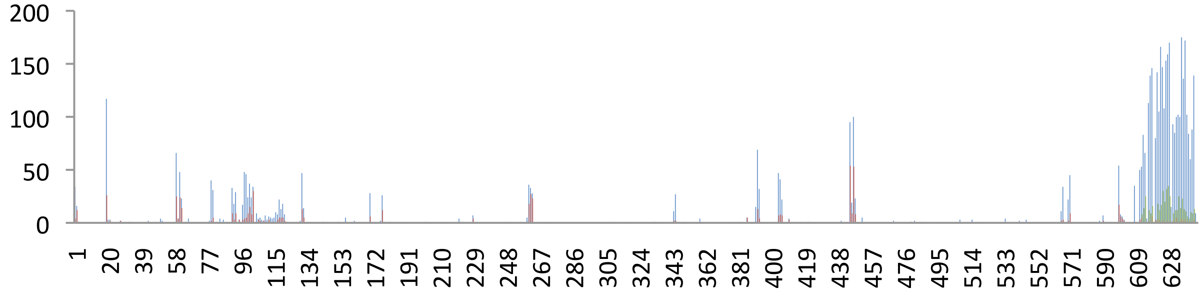

F)

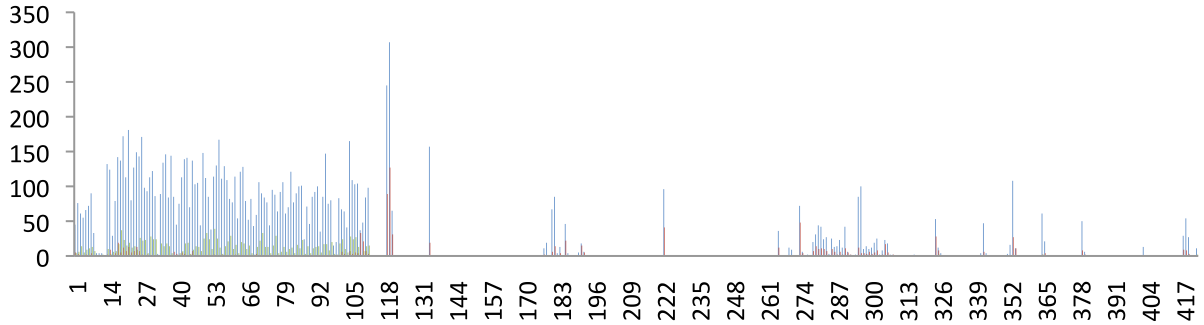

G)

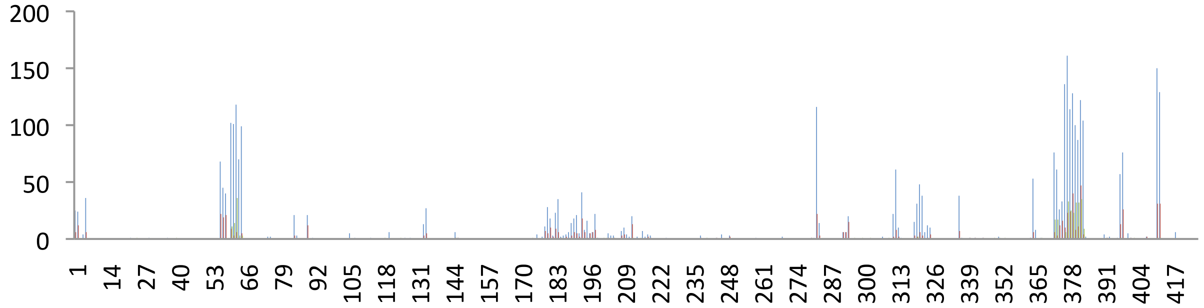

H)

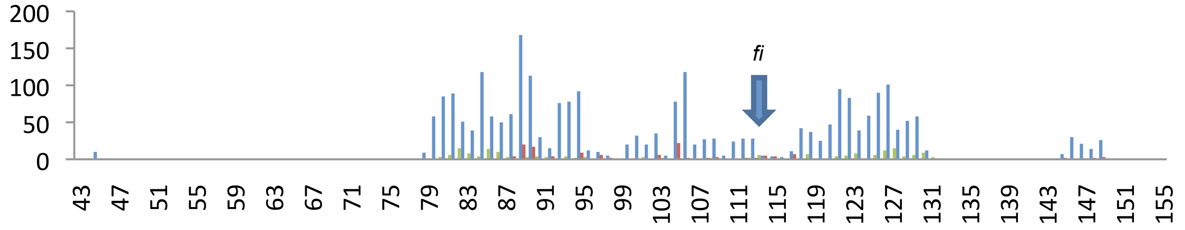

Figure S6 Distribution of polymorphisms in strain 1303. A) Supercontig 1, B) Supercontig 2, C) Supercontig 3, D) Supercontig 4. The location of *fi* is indicated by a vertical arrow, E) Supercontig 5, F) Supercontig 6, G) Supercontig 7, H) The region from 430000 to 1550000 on Supercontig 4. The location of *fi* is indicated by a vertical arrow. Total SNPs are plotted in blue. SNPs that are unique to strain 1303 are plotted in red. Indels are plotted in green. Polymorphisms were sorted by Supercontig and position and the total number in a 10 kb moving window is plotted on the Y axis. The X axis corresponds to the position along the Supercontig. (X 10 kb)

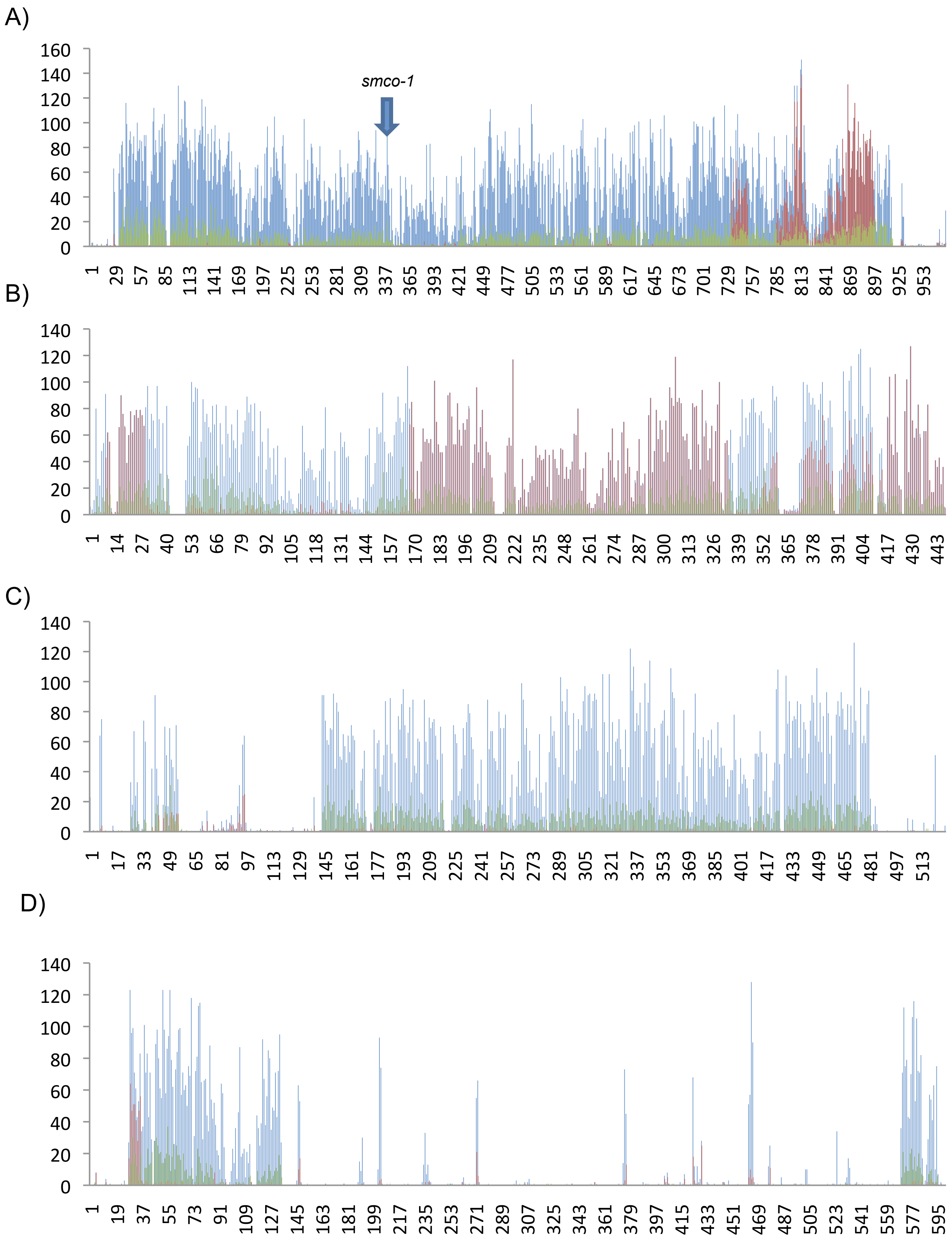
A)
smco-1
B)
C)
D)

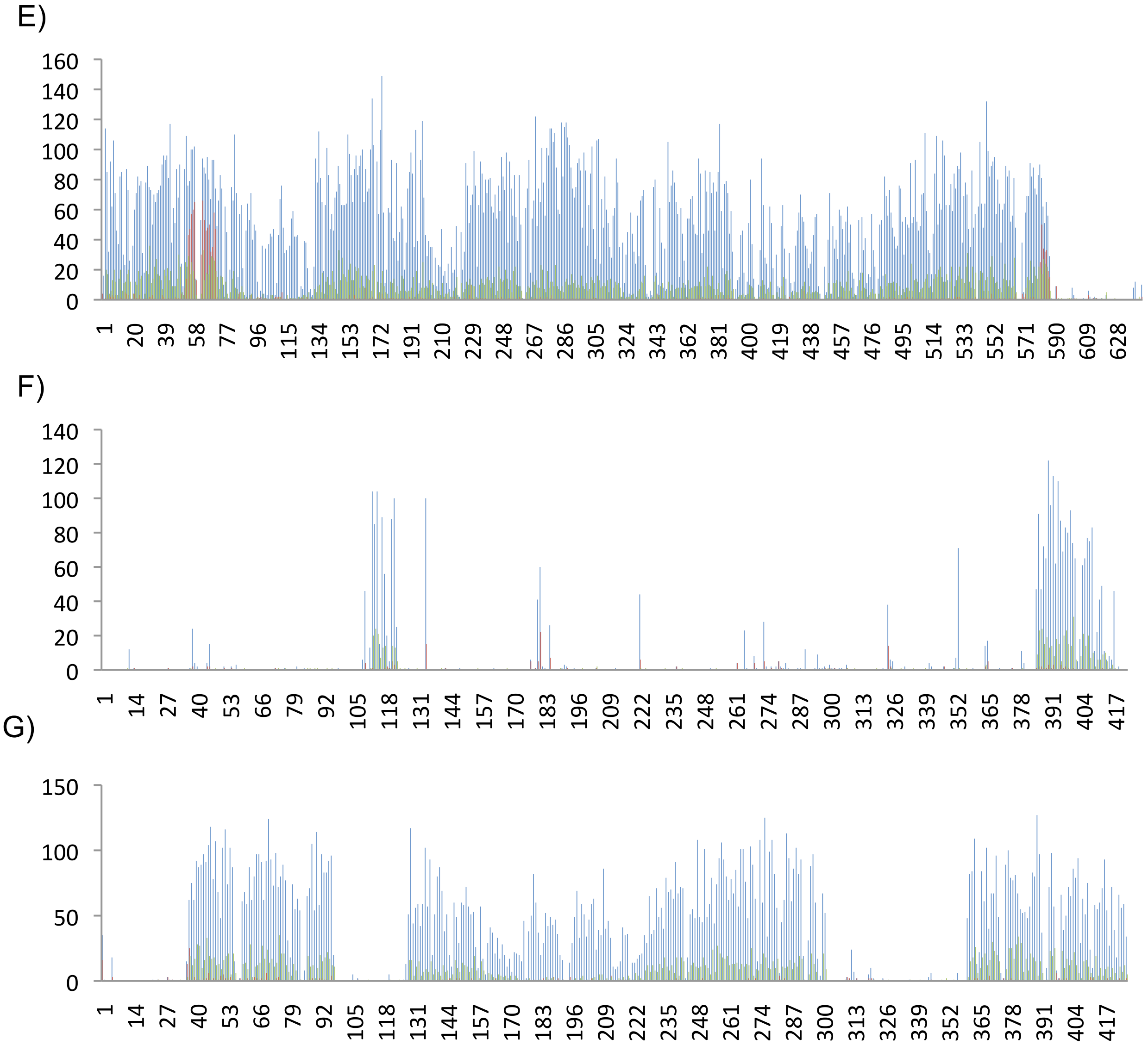

Figure S7 Distribution of polymorphisms in strain 1363. A) Supercontig 1, B) Supercontig 2, C) Supercontig 3, D) Supercontig 4, E) Supercontig 5, F) Supercontig 6, G) Supercontig 7. Total SNPs are plotted in blue. SNPs that are unique to strain 1363 are plotted in red. Indels are plotted in green. Polymorphisms were sorted by Supercontig and position and the total number in a 10 kb moving window is plotted on the Y axis. The X axis corresponds to the position along the Supercontig. (X 10 kb)

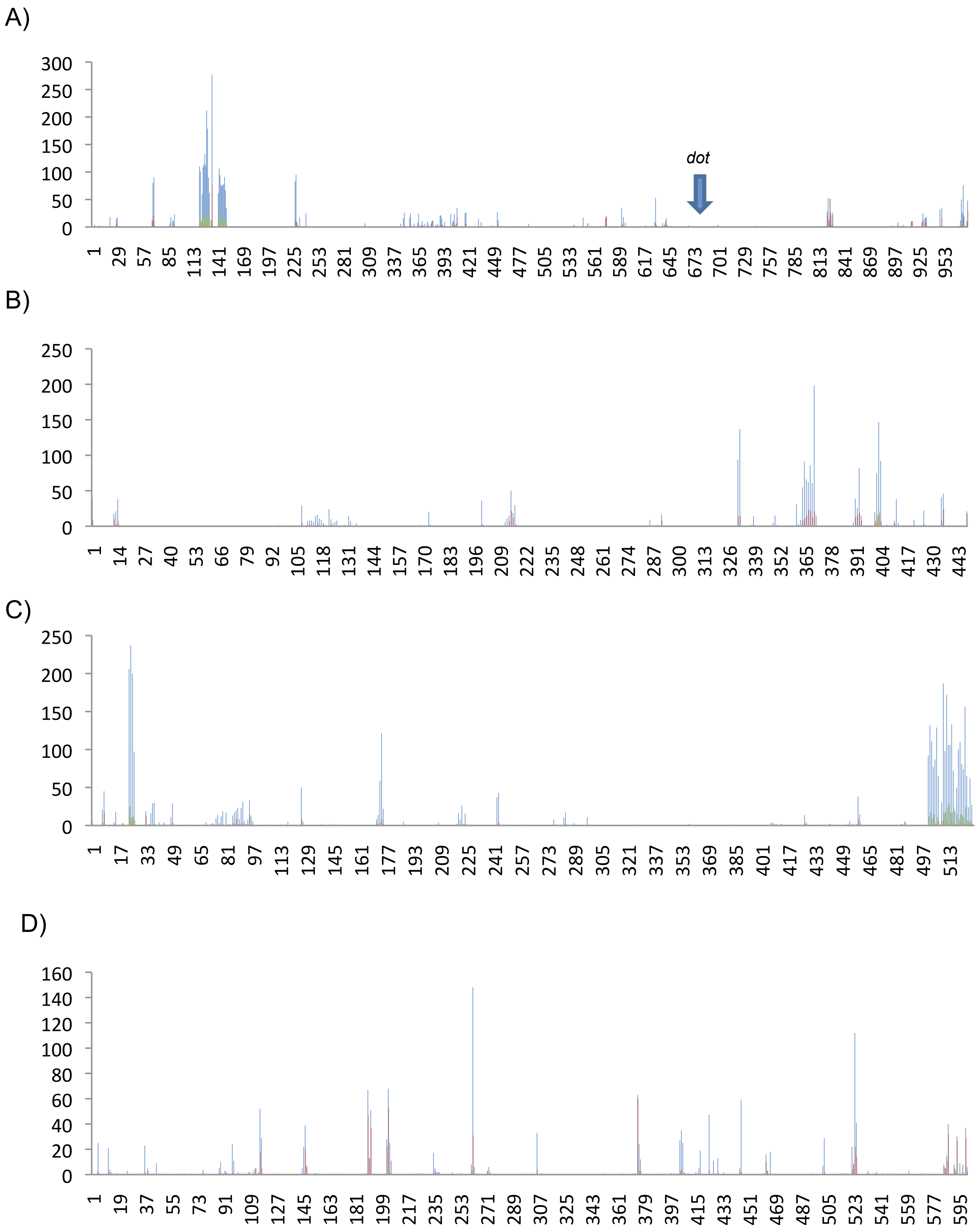
A)
dot
B)
C)
D)

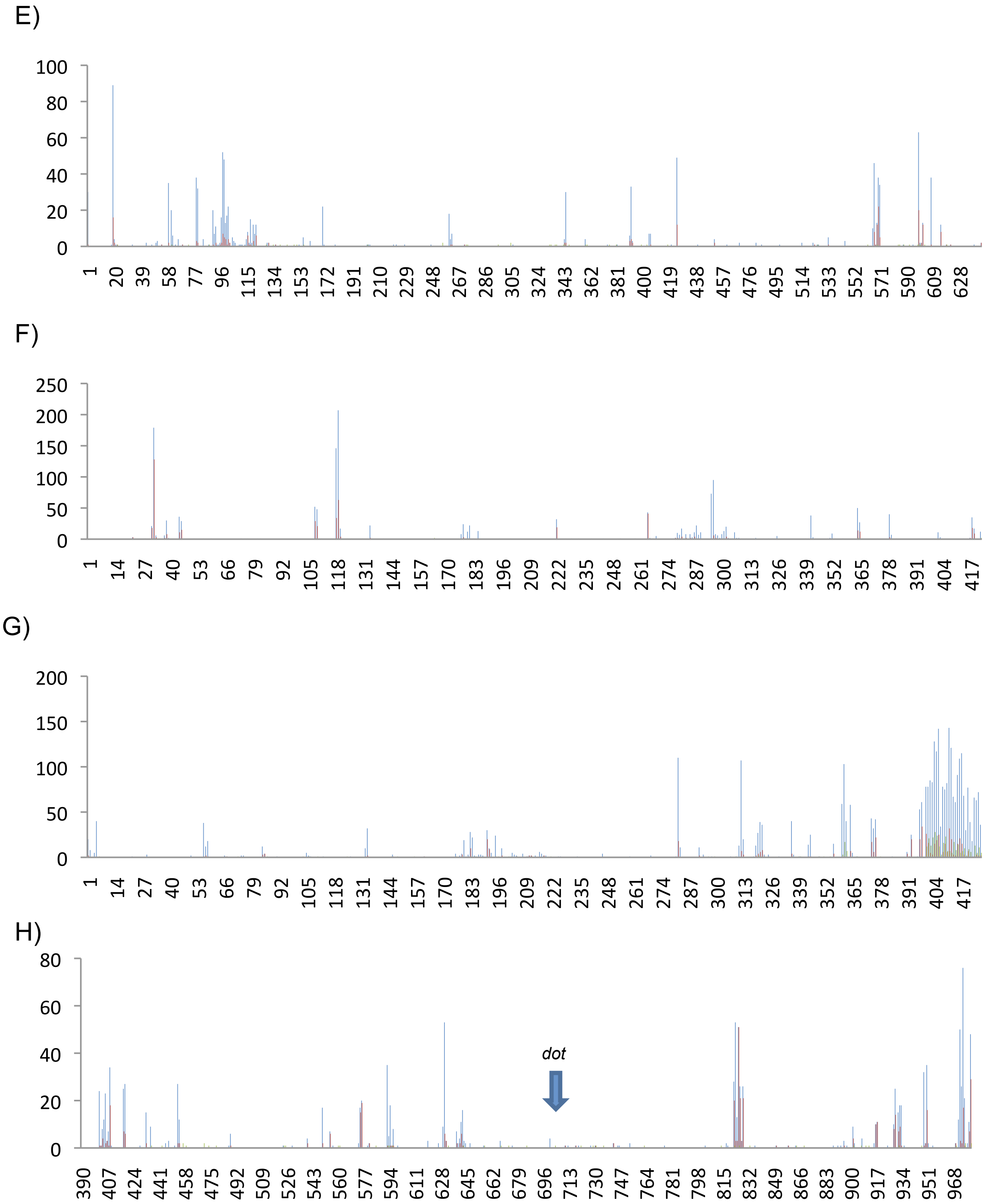

Figure S8 Distribution of polymorphisms in strain 1211. A) Supercontig 1. The location of *dot* is indicated by a vertical arrow, B) Supercontig 2, C) Supercontig 3, D) Supercontig 4, E) Supercontig 5, F) Supercontig 6, G) Supercontig 7, H) The region >3970000 on Supercontig 1. The location of *dot* is indicated by a vertical arrow. Total SNPs are plotted in blue. SNPs that are unique to strain 1211 are plotted in red. Indels are plotted in green. Polymorphisms were sorted by Supercontig and position and the total number in a 10 kb moving window is plotted on the Y axis. The X axis corresponds to the position along the Supercontig. Values are X 10kb.

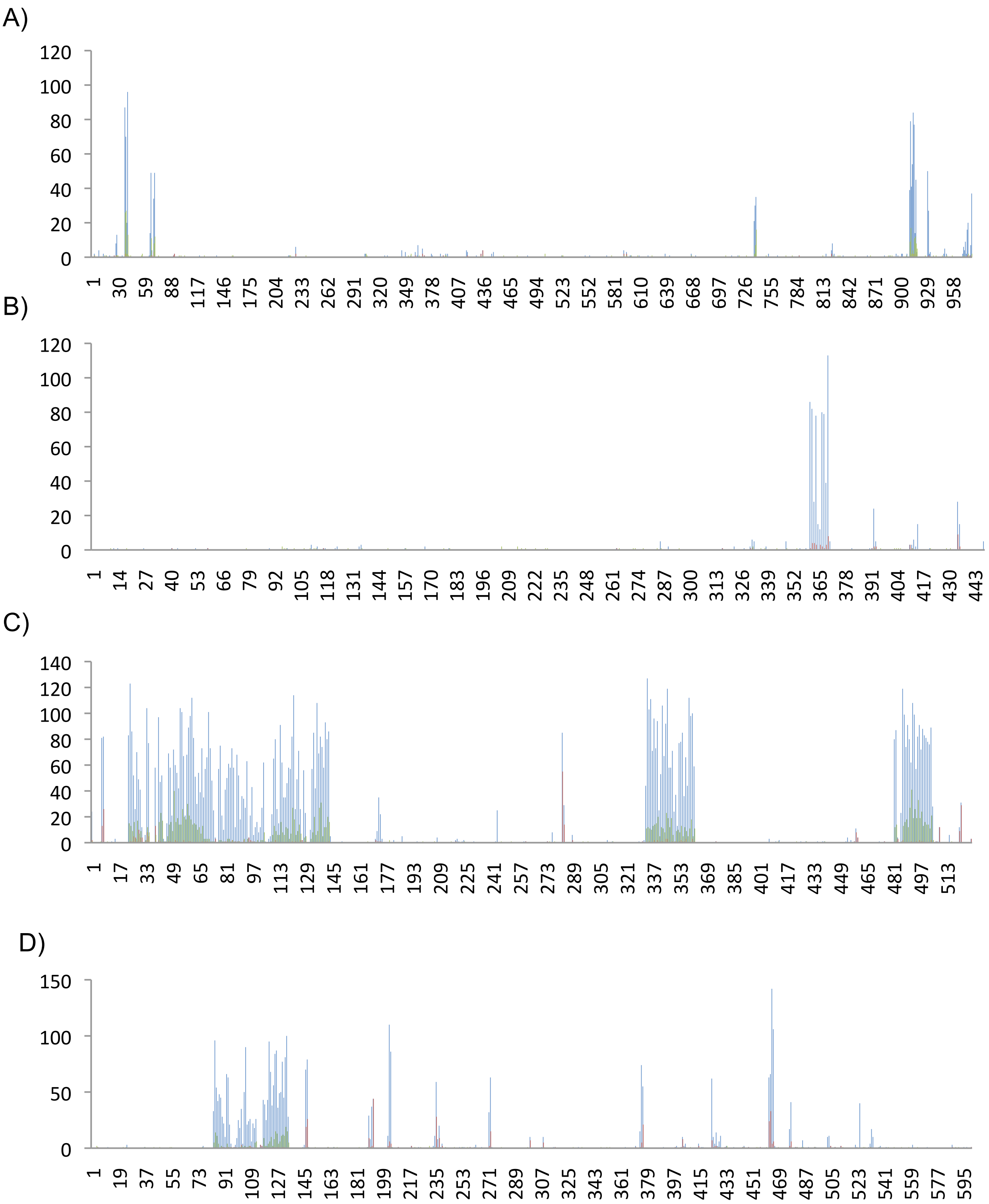
A)
120
100
80
60
40
20
0
1 30 59 88 117 146 175 204 233 262 291 320 349 378 407 436 465 494 523 552 581 610 639 668 697 726 755 784 813 842 871 900 929 958
B)
120
100
80
60
40
20
0
1 14 27 40 53 66 79 92 105 118 131 144 157 170 183 196 209 222 235 248 261 274 287 300 313 326 339 352 365 378 391 404 417 430 443
C)
140
120
100
80
60
40
20
0
1 17 33 49 65 81 97 113 129 145 161 177 193 209 225 241 257 273 289 305 321 337 353 369 385 401 417 433 449 465 481 497 513
D)
150
100
50
0
1 19 37 55 73 91 109 127 145 163 181 199 217 235 253 271 289 307 325 343 361 379 397 415 433 451 469 487 505 523 541 559 577 595

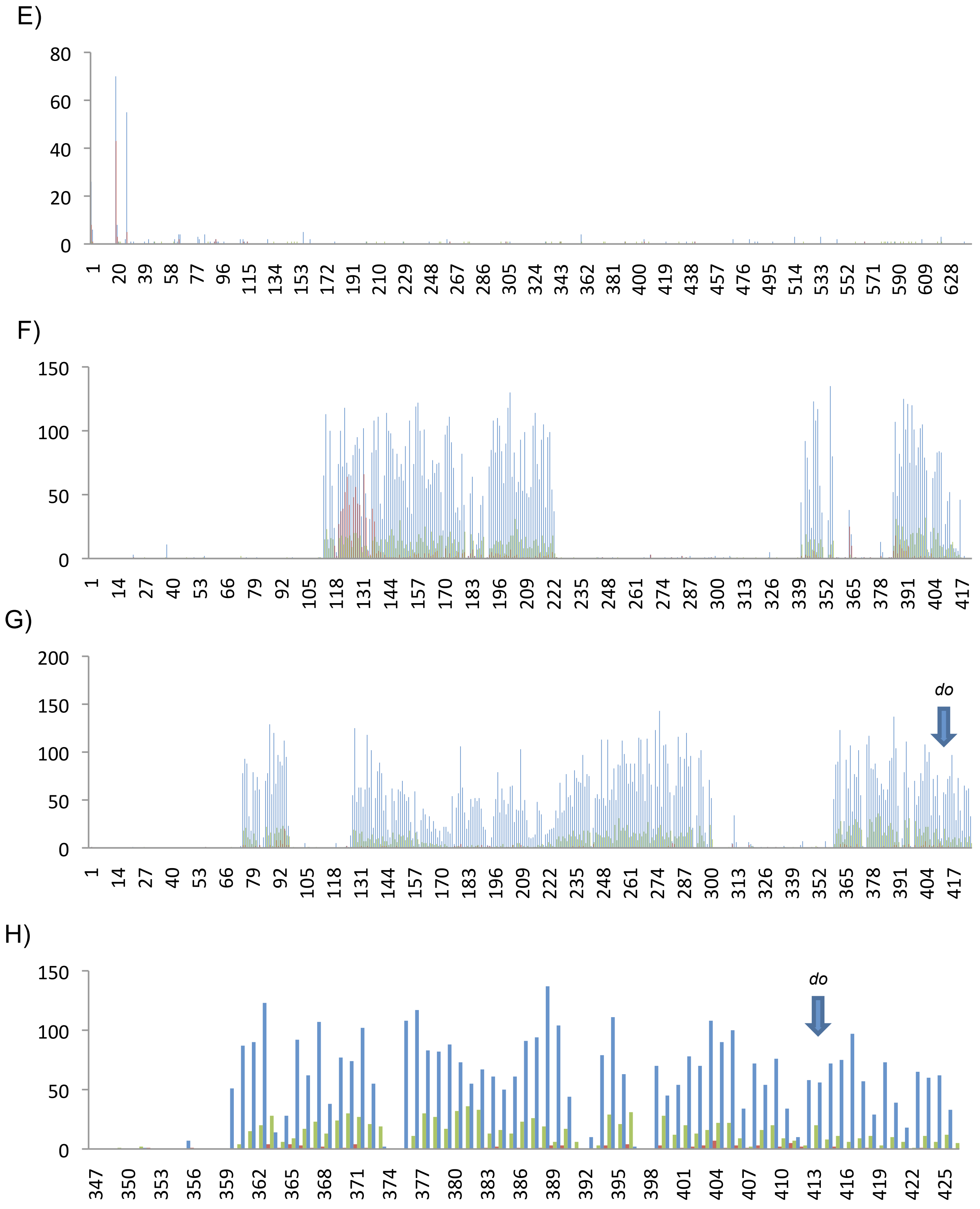

Figure S9 Distribution of polymorphisms in strain 2261. A) Supercontig 1, B) Supercontig 2, C) Supercontig 3, D) Supercontig 4, E) Supercontig 5, F) Supercontig 6, G) Supercontig 7. The location of *do* is indicated by a vertical arrow, H) the region >3475561 on Supercontig 7. The location of *do* is indicated by a vertical arrow. Total SNPs are plotted in blue. SNPs that are unique to strain 2261are plotted in red. Indels are plotted in green. Polymorphisms were sorted by Supercontig and position and the total number in a 10 kb moving window is plotted on the Y axis. The X axis corresponds to the position along the Supercontig(X 10 kb).

A)

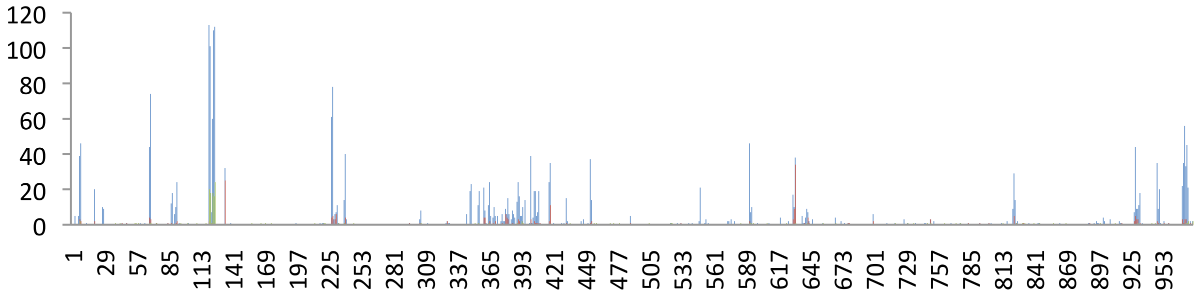
0
20
40
60
80
100
120
1 29 57 85 113 141 169 197 225 253 281 309 337 365 393 421 449 477 505 533 561 589 617 645 673 701 729 757 785 813 841 869 897 925 953

B)

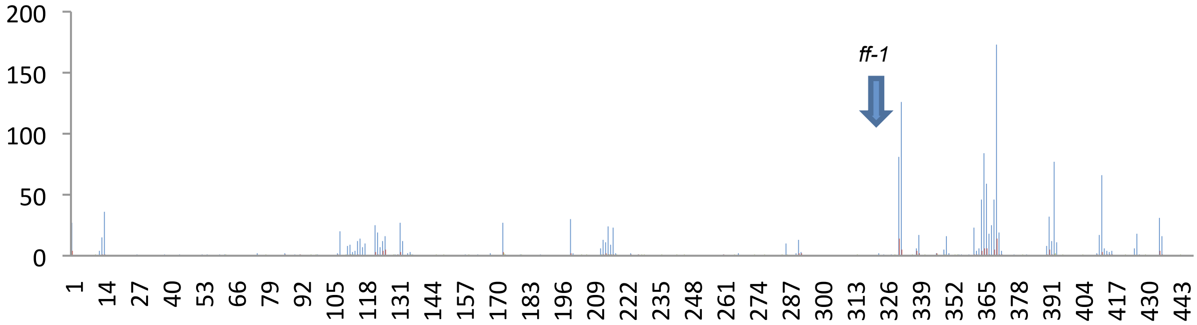
ff-1
0
50
100
150
200
1 14 27 40 53 66 79 92 105 118 131 144 157 170 183 196 209 222 235 248 261 274 287 300 313 326 339 352 365 378 391 404 417 430 443

C)

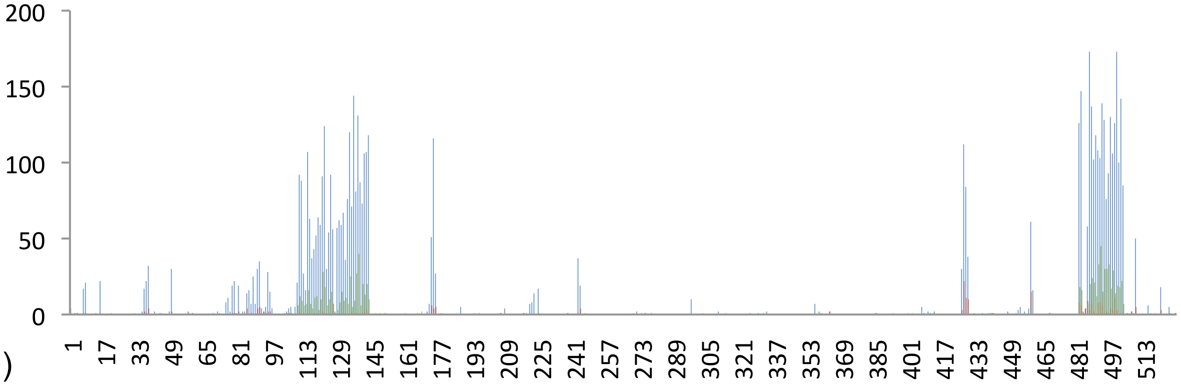
0
50
100
150
200
1 17 33 49 65 81 97 113 129 145 161 177 193 209 225 241 257 273 289 305 321 337 353 369 385 401 417 433 449 465 481 497 513

D)

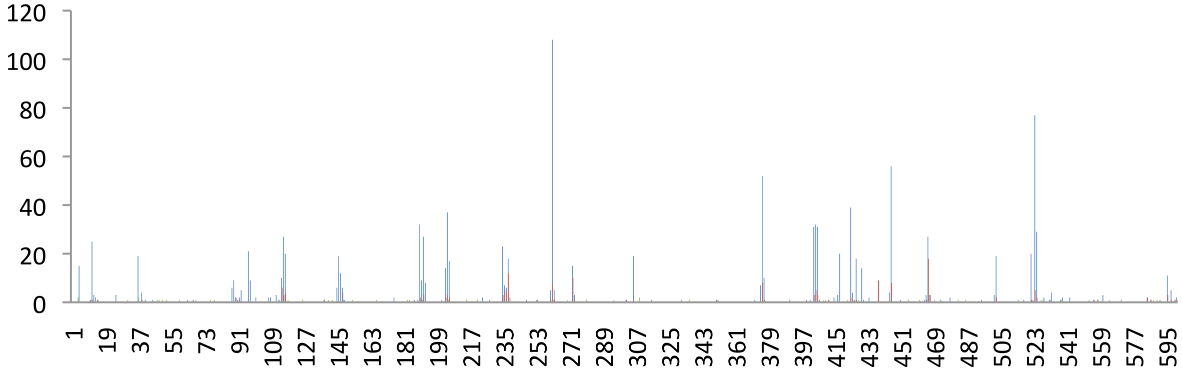
0
20
40
60
80
100
120
1 19 37 55 73 91 109 127 145 163 181 199 217 235 253 271 289 307 325 343 361 379 397 415 433 451 469 487 505 523 541 559 577 595

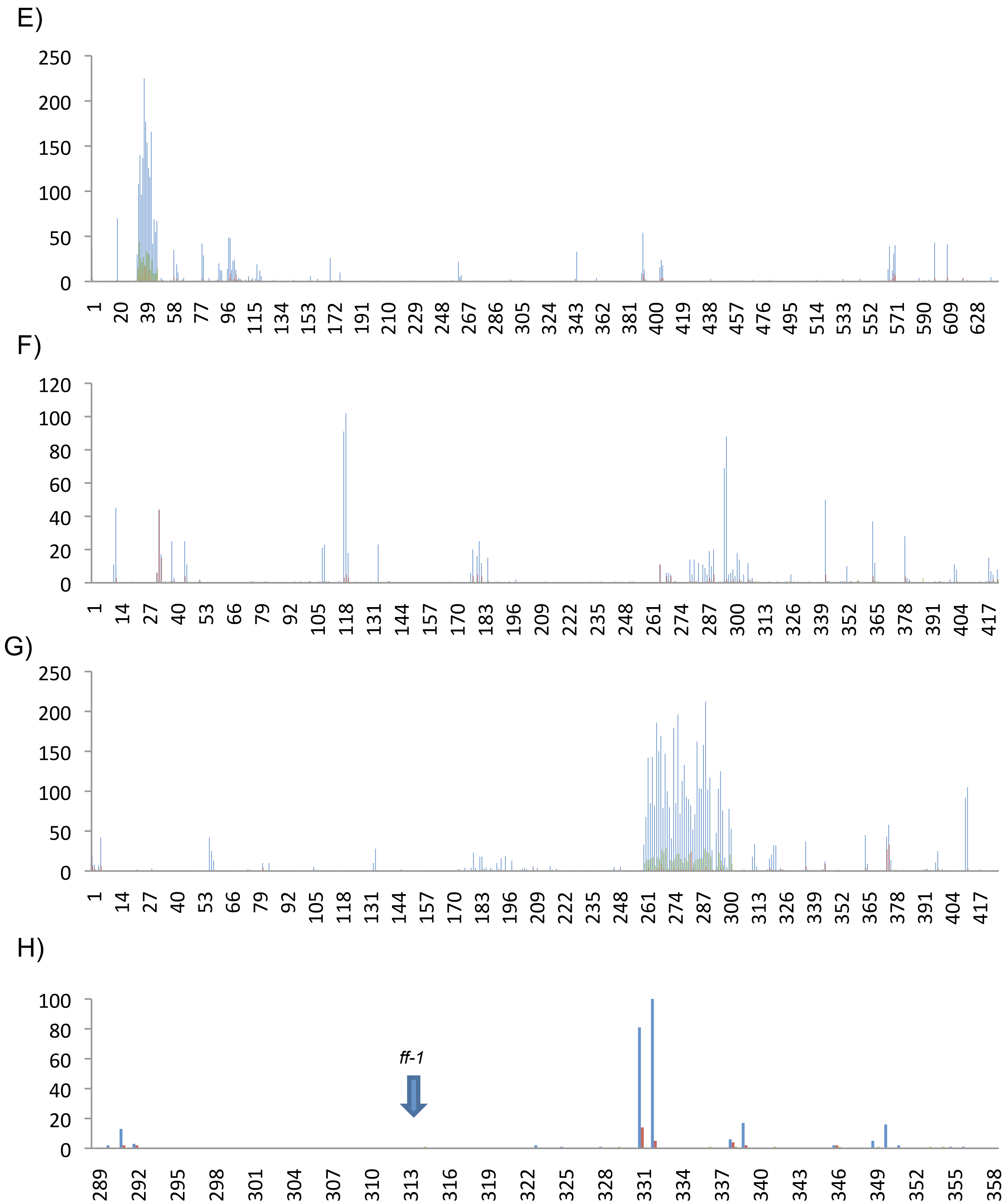

Figure S10 Distribution of polymorphisms in strain 3831. A) Supercontig 1, B) Supercontig 2. The location of *ff-1*is indicated by a vertical arrow, C) Supercontig 3, D) Supercontig 4, E) Supercontig 5, F) Supercontig 6, G) Supercontig 7, H) The region from 2897659 to 3571946 on Supercontig 2. The location of *ff-1* is indicated by a vertical arrow. Total SNPs are plotted in blue. SNPs that are unique to strain 3831 are plotted in red. Indels are plotted in green. Polymorphisms were sorted by Supercontig and position and the total number in a 10 kb moving window is plotted on the Y axis. The X axis corresponds to the position along the Supercontig. (X 10 kb)

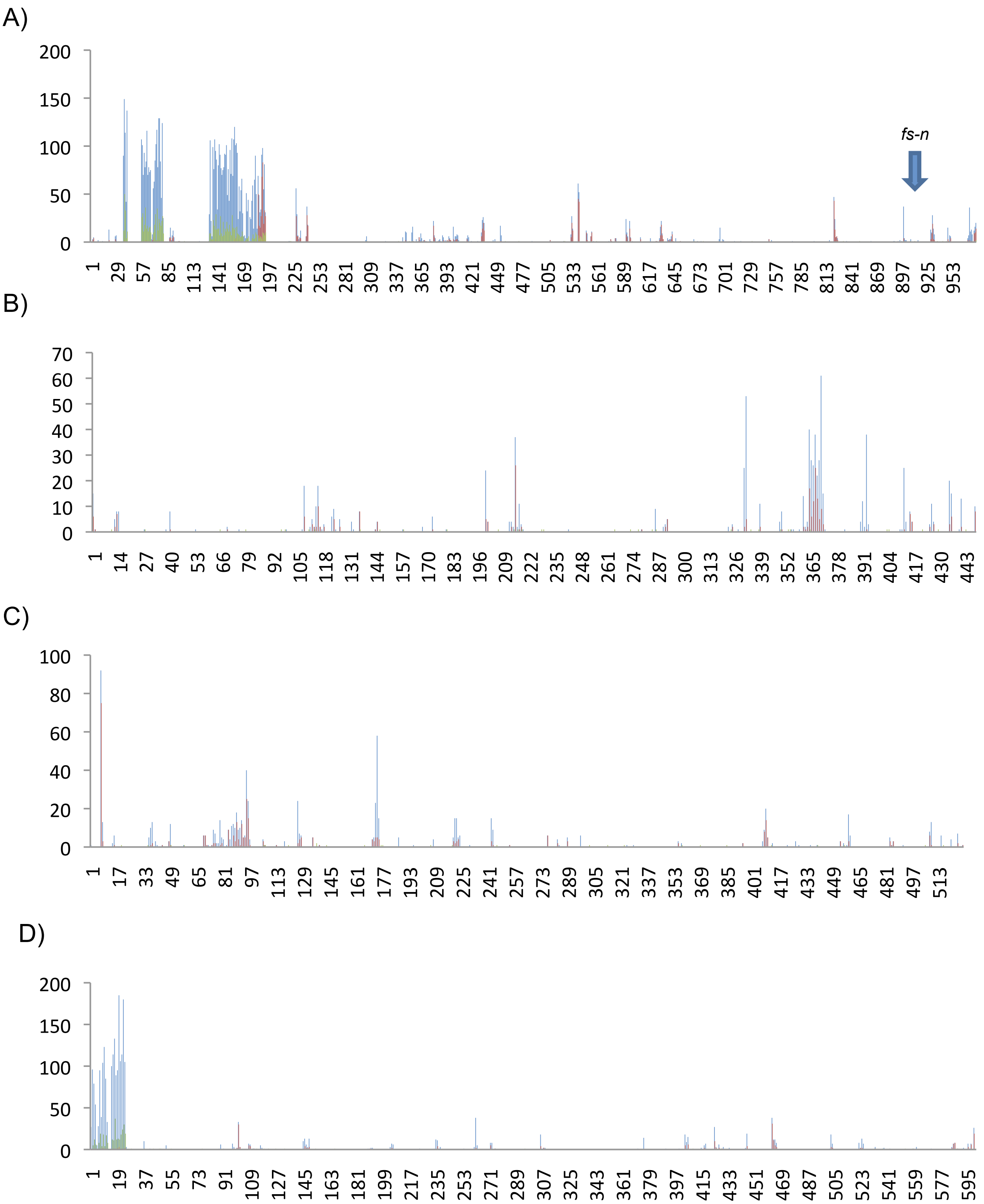
A)
200
150
100
50
0
fs-n
1 29 57 85 113 141 169 197 225 253 281 309 337 365 393 421 449 477 505 533 561 589 617 645 673 701 729 757 785 813 841 869 897 925 953
B)
70
60
50
40
30
20
10
0
1 14 27 40 53 66 79 92 105 118 131 144 157 170 183 196 209 222 235 248 261 274 287 300 313 326 339 352 365 378 391 404 417 430 443
C)
100
80
60
40
20
0
1 17 33 49 65 81 97 113 129 145 161 177 193 209 225 241 257 273 289 305 321 337 353 369 385 401 417 433 449 465 481 497 513
D)
200
150
100
50
0
1 19 37 55 73 91 109 127 145 163 181 199 217 235 253 271 289 307 325 343 361 379 397 415 433 451 469 487 505 523 541 559 577 595

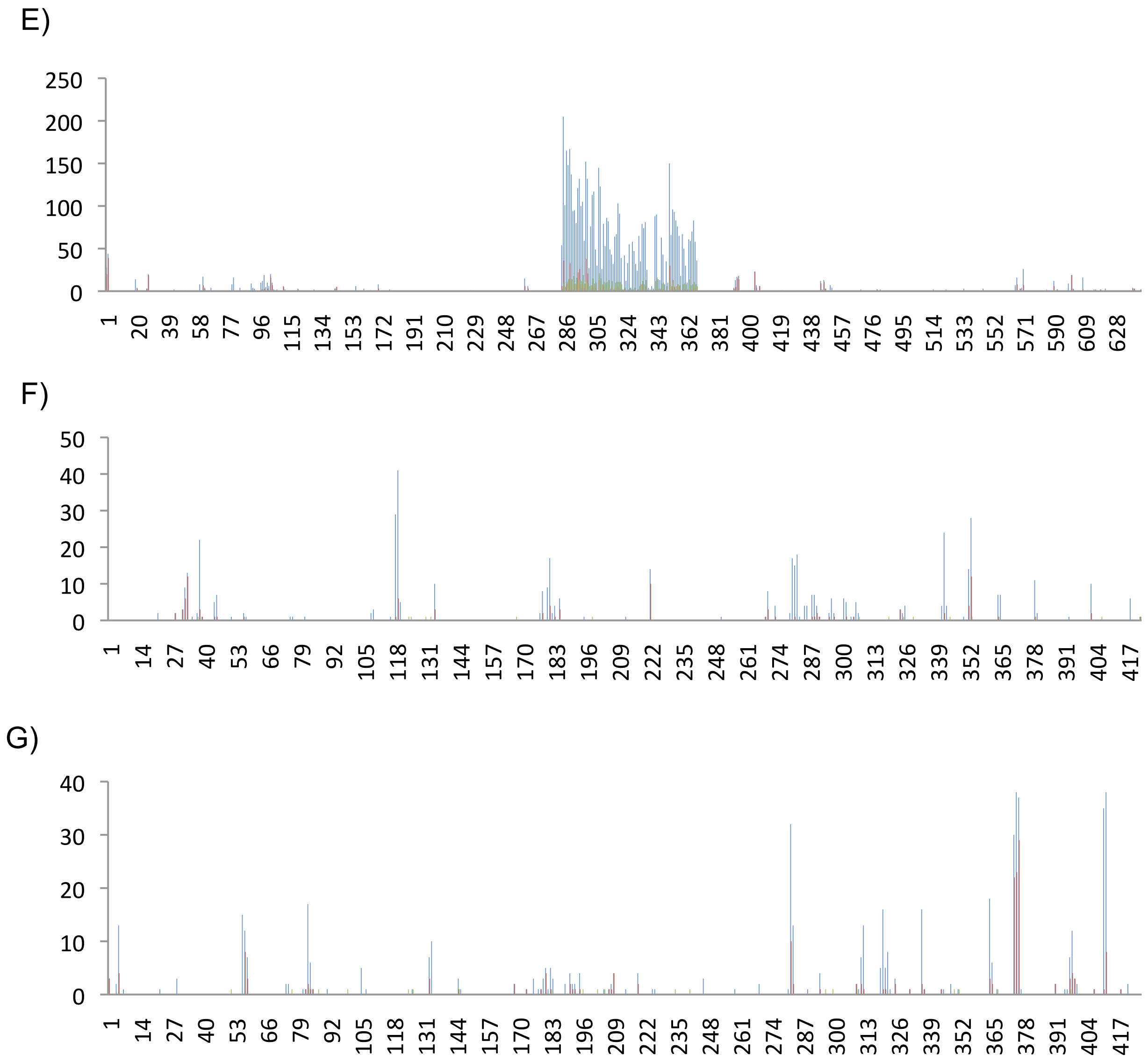

Figure S11 Distribution of polymorphisms in strain 3246. A) Supercontig 1. The location of *fs-n* is indicated by a vertical arrow, B) Supercontig 2, C) Supercontig 3, D) Supercontig 4, E) Supercontig 5, F) Supercontig 6, G) Supercontig 7. Total SNPs are plotted in blue. SNPs that are unique to strain 3246 are plotted in red. Indels are plotted in green. Polymorphisms were sorted by Supercontig and position and the total number in a 10 kb moving window is plotted on the Y axis. The X axis corresponds to the position along the Supercontig. Values are X 10 kb.

A)

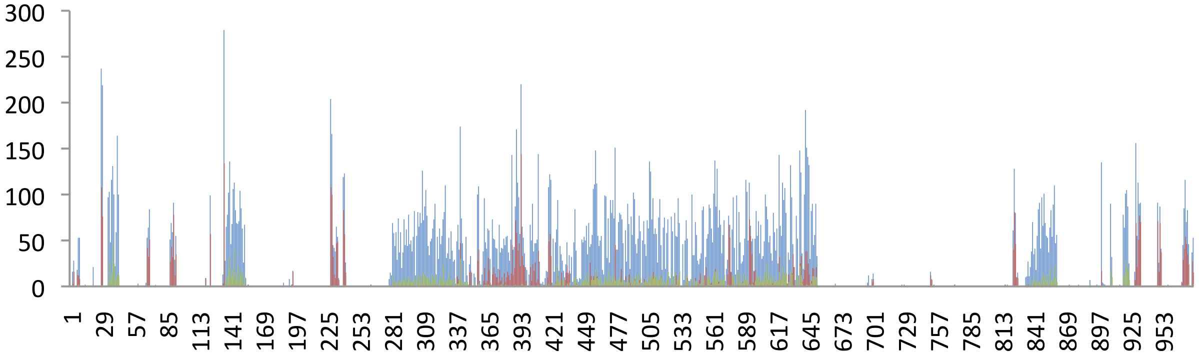
300
250
200
150
100
50
0
1 29 57 85 113 141 169 197 225 253 281 309 337 365 393 421 449 477 505 533 561 589 617 645 673 701 729 757 785 813 841 869 897 925 953

B)

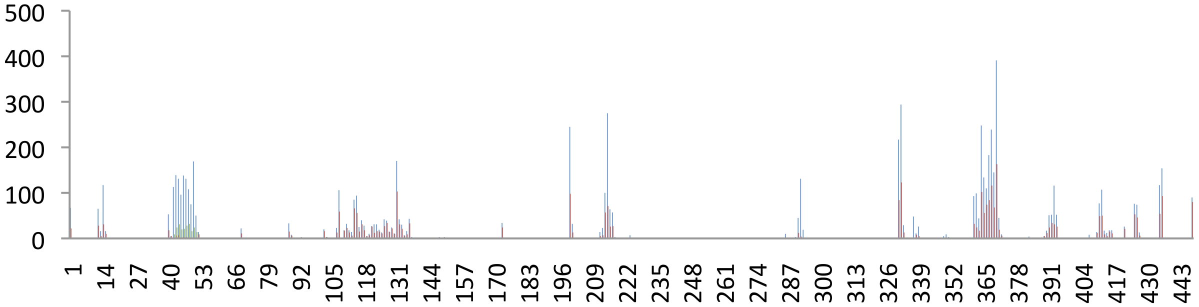
500
400
300
200
100
0
1 14 27 40 53 66 79 92 105 118 131 144 157 170 183 196 209 222 235 248 261 274 287 300 313 326 339 352 365 378 391 404 417 430 443

C)

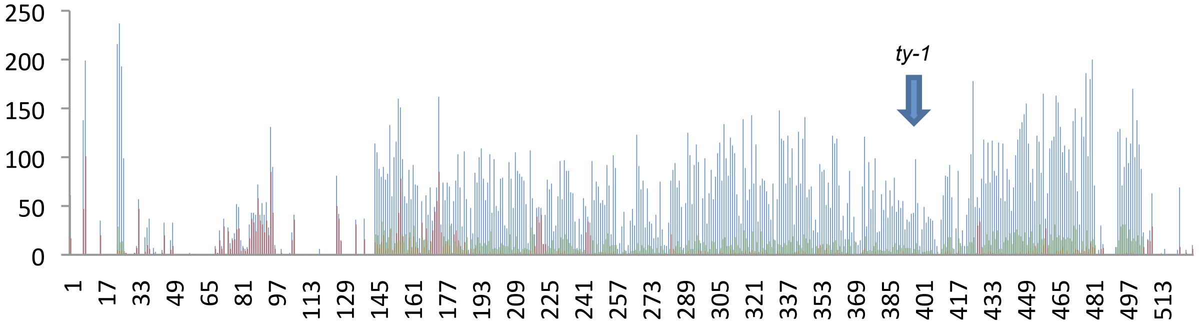
250
200
150
100
50
0
ty-1
1 17 33 49 65 81 97 113 129 145 161 177 193 209 225 241 257 273 289 305 321 337 353 369 385 401 417 433 449 465 481 497 513

D)

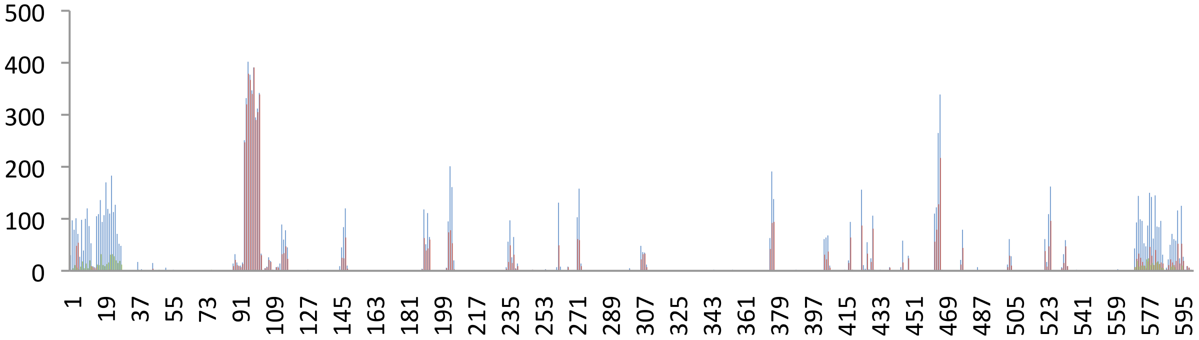
500
400
300
200
100
0
1 19 37 55 73 91 109 127 145 163 181 199 217 235 253 271 289 307 325 343 361 379 397 415 433 451 469 487 505 523 541 559 577 595

E)

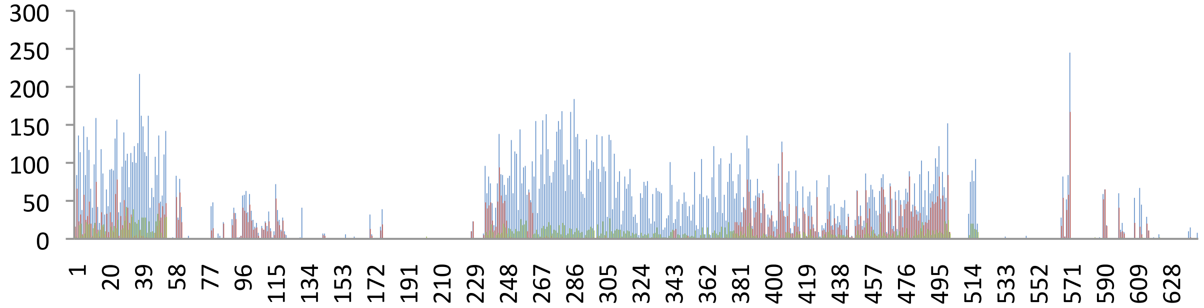

F)

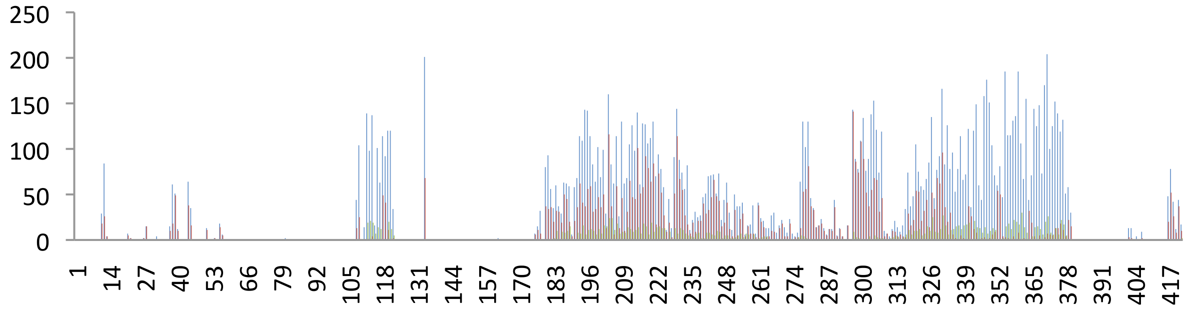

G)

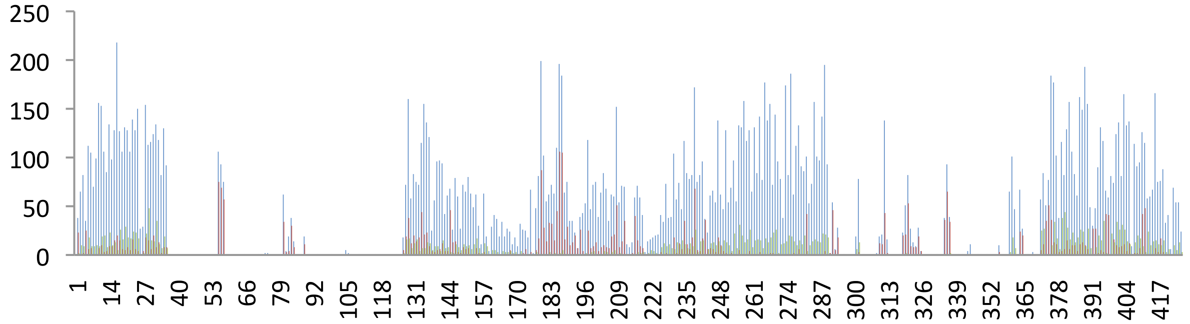

H)

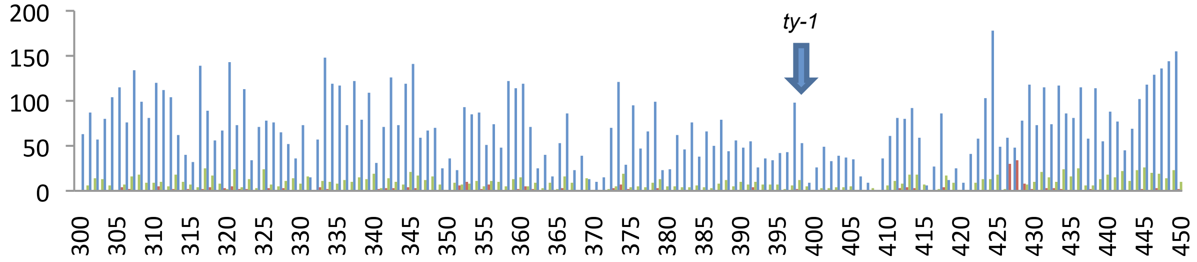

Figure S12 Distribution of polymorphisms in strain 322. A) Supercontig 1, B) Supercontig 2, C) Supercontig 3. The location of *ty-1* is indicated by a vertical arrow. , D) Supercontig 4, E) Supercontig 5, F) Supercontig 6, G) Supercontig 7, H) The region from 3000000 to 4500000 on Supercontig 3. The location of *ty-1* is indicated by a vertical arrow. Total SNPs are plotted in blue. SNPs that are unique to strain 322 are plotted in red. Indels are plotted in green. Polymorphisms were sorted by Supercontig and position and the total number in a 10 kb moving window is plotted on the Y axis. The X axis corresponds to the position along the Supercontig.

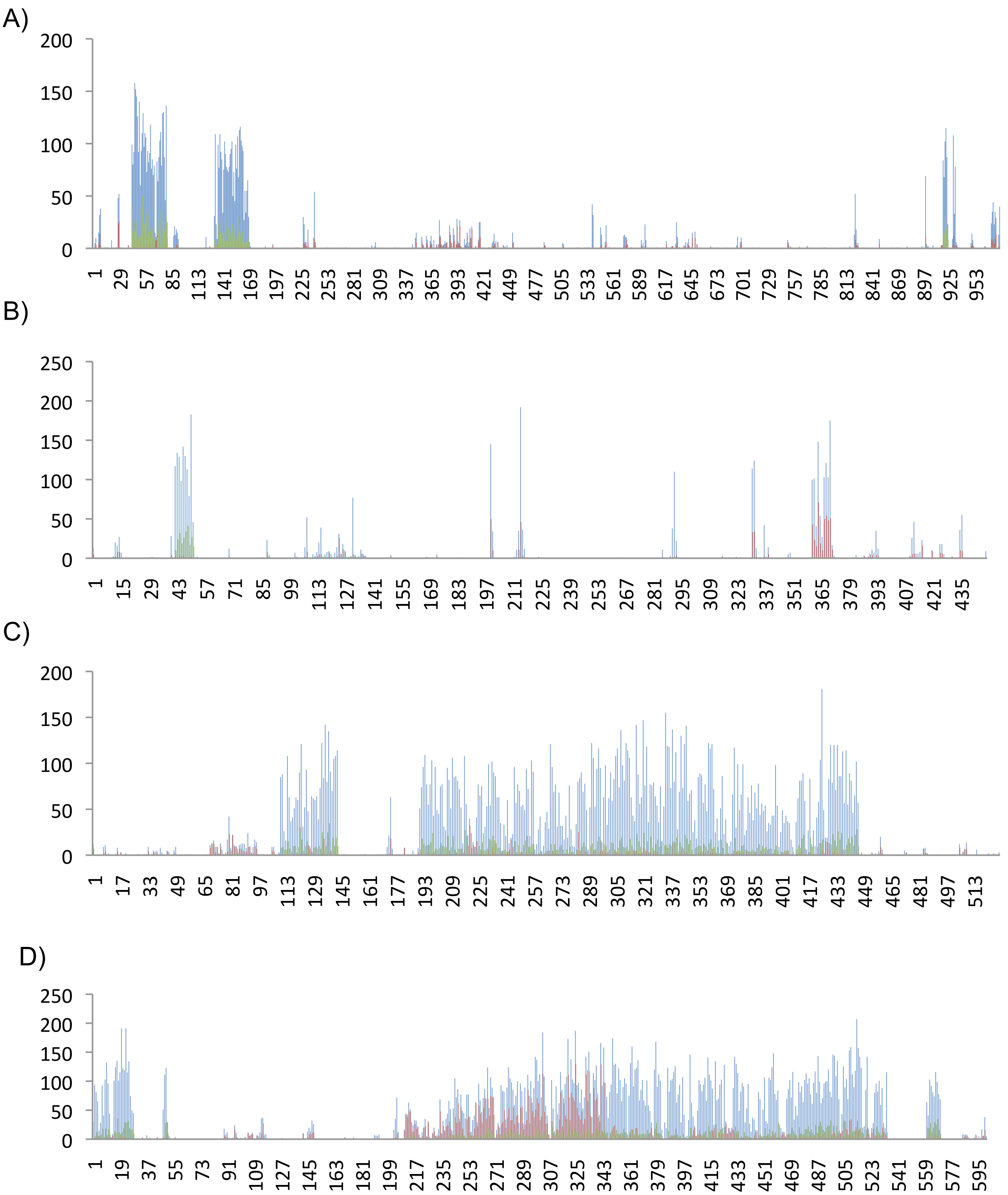
A)
200
150
100
50
0
1 29 57 85 113 141 169 197 225 253 281 309 337 365 393 421 449 477 505 533 561 589 617 645 673 701 729 757 785 813 841 869 897 925 953
B)
250
200
150
100
50
0
1 15 29 43 57 71 85 99 113 127 141 155 169 183 197 211 225 239 253 267 281 295 309 323 337 351 365 379 393 407 421 435
C)
200
150
100
50
0
1 17 33 49 65 81 97 113 129 145 161 177 193 209 225 241 257 273 289 305 321 337 353 369 385 401 417 433 449 465 481 497 513
D)
250
200
150
100
50
0
1 19 37 55 73 91 109 127 145 163 181 199 217 235 253 271 289 307 325 343 361 379 397 415 433 451 469 487 505 523 541 559 577 595

E)

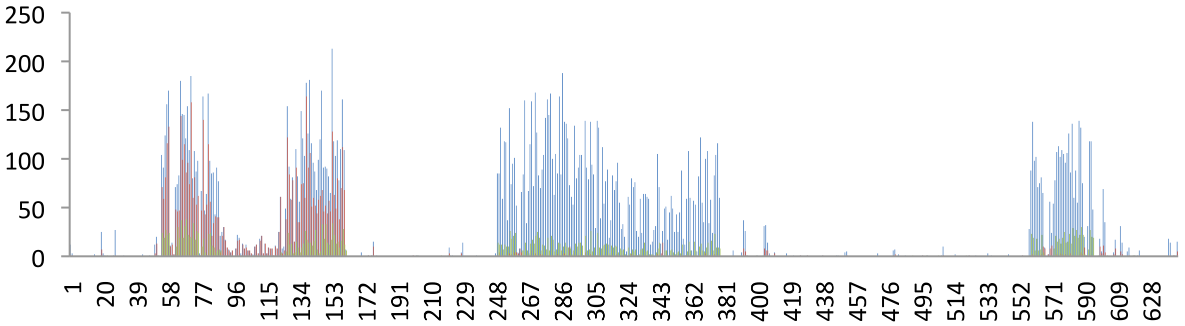

F)

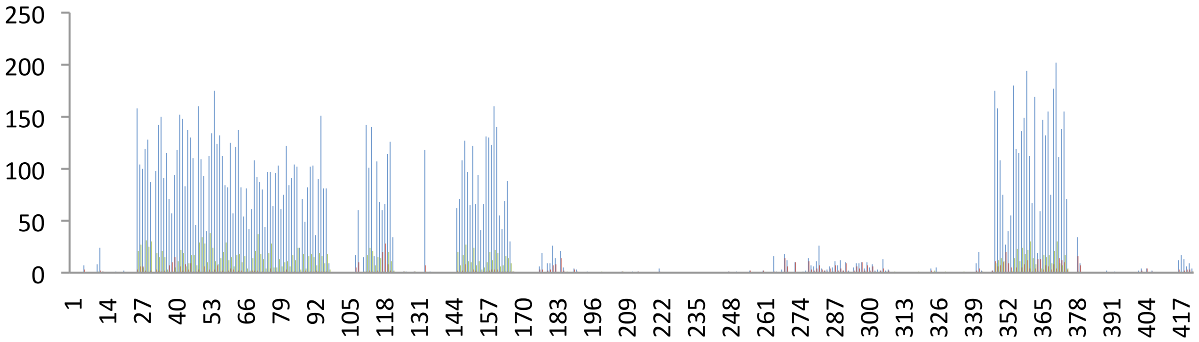

G)

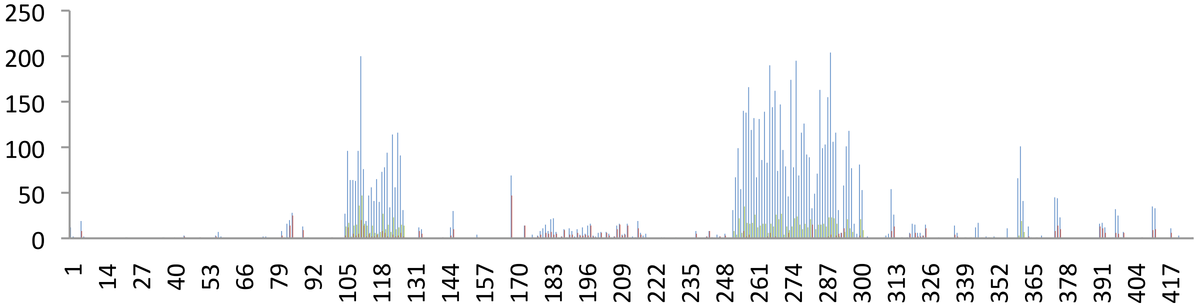

Figure S13 Distribution of polymorphisms in strain 3562. A) Supercontig 1, B) Supercontig 2, C) Supercontig 3, D) Supercontig 4, E) Supercontig 5, F) Supercontig 6, G) Supercontig 7. Total SNPs are plotted in blue. SNPs that are unique to strain 3562 are plotted in red. Indels are plotted in green. Polymorphisms were sorted by Supercontig and position and the total number in a 10 kb moving window is plotted on the Y axis. The X axis corresponds to the position along the Supercontig. Values are X 10 kb.

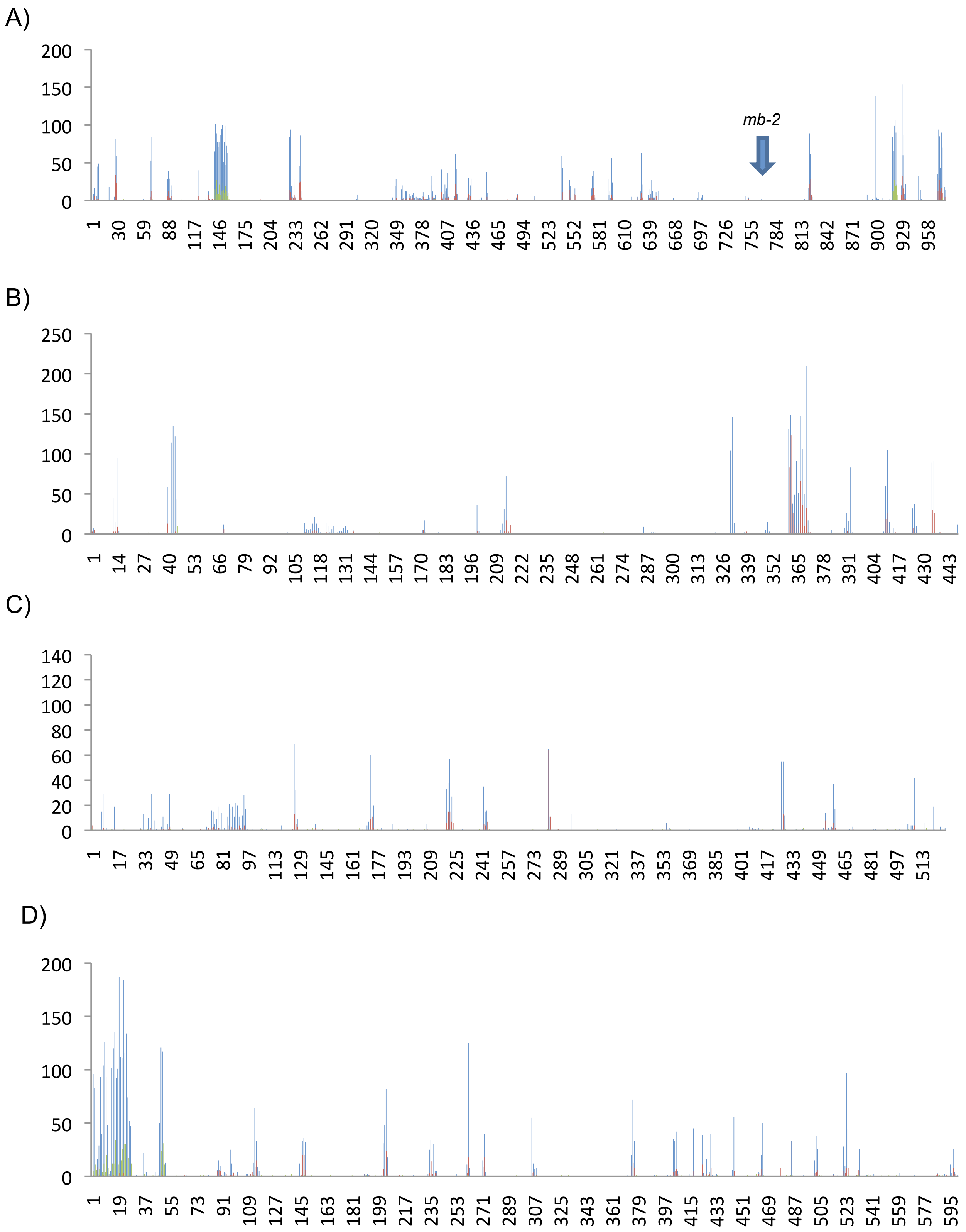
A)
mb-2
B)
C)
D)

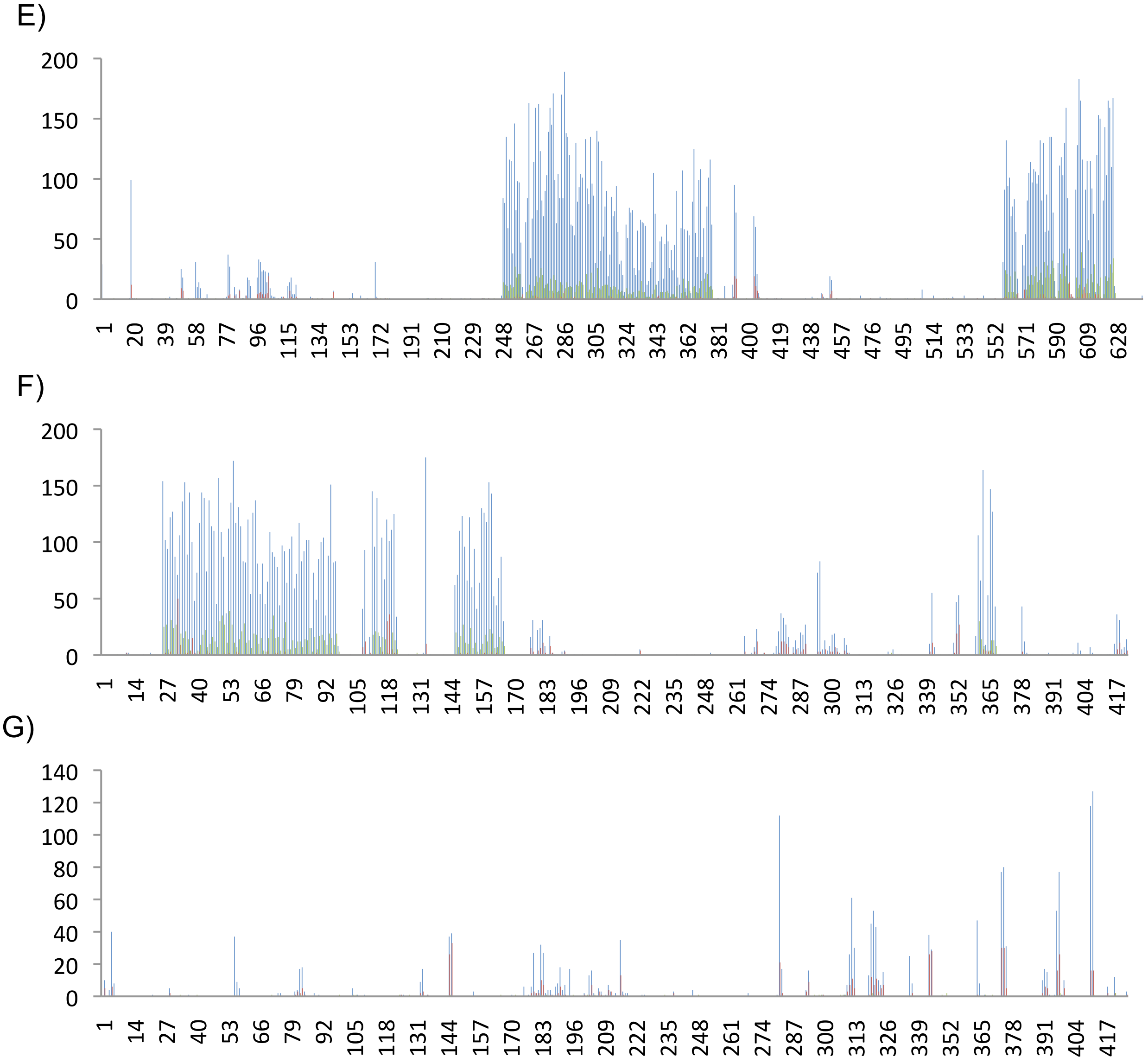

Figure S14 Distribution of polymorphisms in strain 3564. A) Supercontig 1. The location of *mb-2* is indicated by a vertical arrow. There are only eight SNPs in the region bounded by the flanking markers in this strain, B) Supercontig 2, C) Supercontig 3, D) Supercontig 4, E) Supercontig 5, F) Supercontig 6, G) Supercontig 7. Total SNPs are plotted in blue. SNPs that are unique to strain 3564 are plotted in red. Indels are plotted in green. Polymorphisms were sorted by Supercontig and position and the total number in a 10 kb moving window is plotted on the Y axis. The X axis corresponds to the position along the Supercontig (X 10 kb).

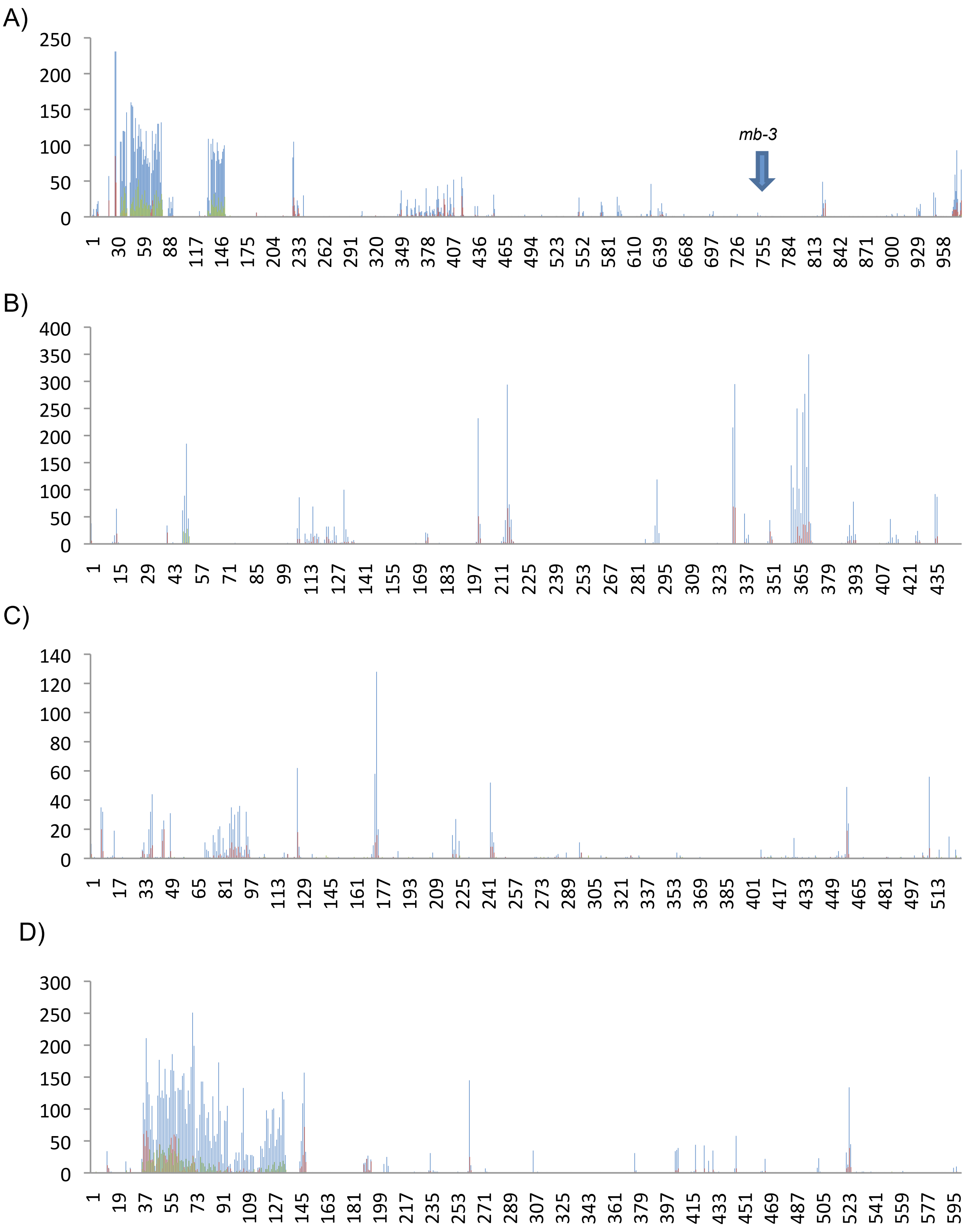
A)
mb-3
B)
C)
D)

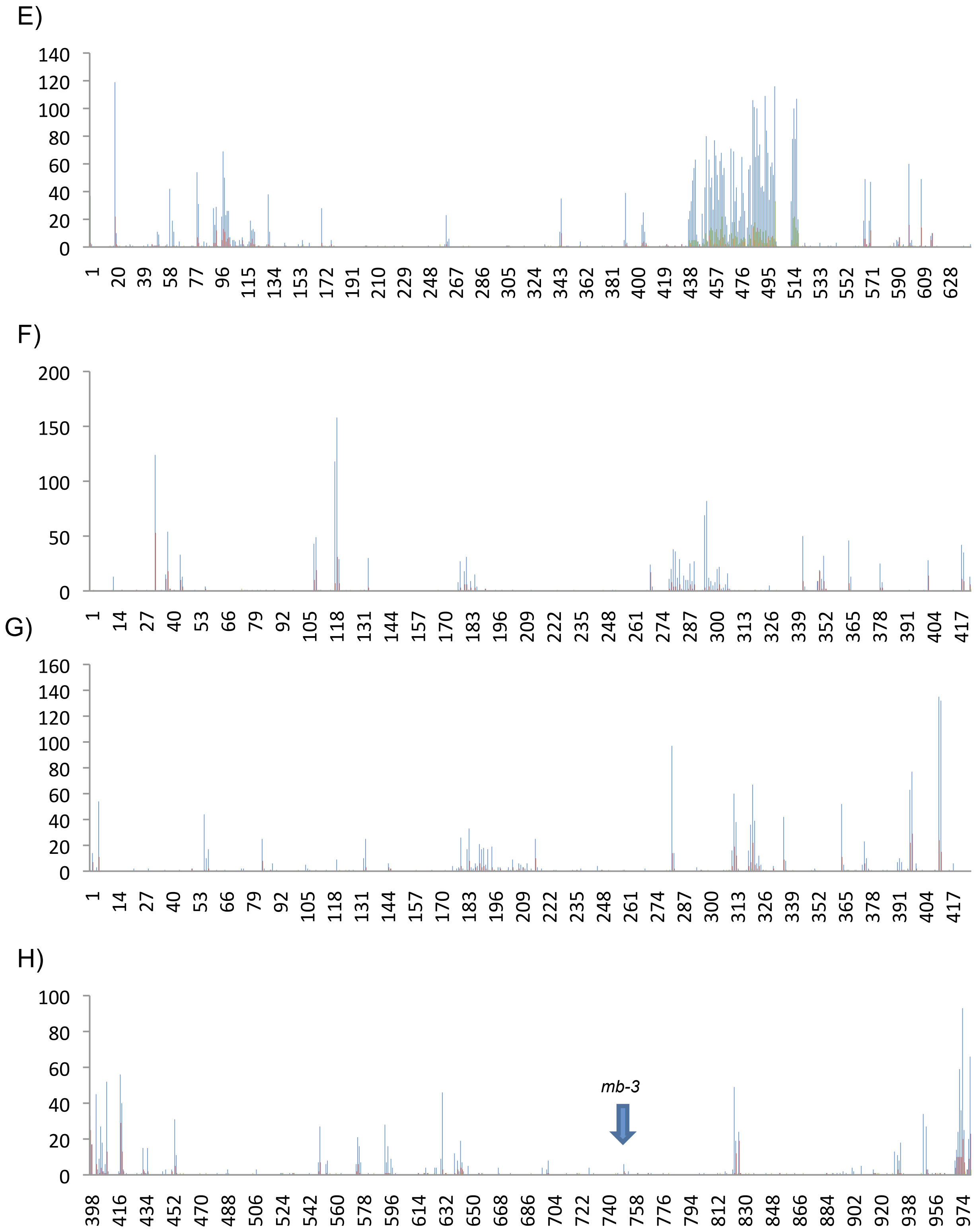

Figure S15 Distribution of polymorphisms in strain 3566. A) Supercontig 1. The location of *mb-3* is indicated by a vertical arrow. B) Supercontig 2, C) Supercontig 3, D) Supercontig 4, E) Supercontig 5, F) Supercontig 6, G) Supercontig 7, H) The region to the right of *cen-1* on Supercontig 1. The location of *mb-3* is indicated by a vertical arrow. Total SNPs are plotted in blue. SNPs that are unique to strain 3566 are plotted in red. Indels are plotted in green. Polymorphisms were sorted by Supercontig and position and the total number in a 10 kb moving window is plotted on the Y axis. The X axis corresponds to the position along the Supercontig. (X 10 kb)

A)

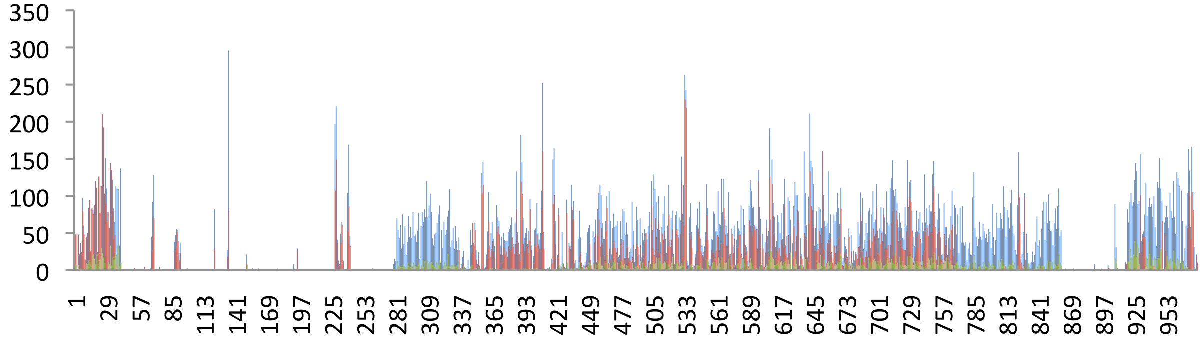
350
300
250
200
150
100
50
0
1 29 57 85 113 141 169 197 225 253 281 309 337 365 393 421 449 477 505 533 561 589 617 645 673 701 729 757 785 813 841 869 897 925 953

B)

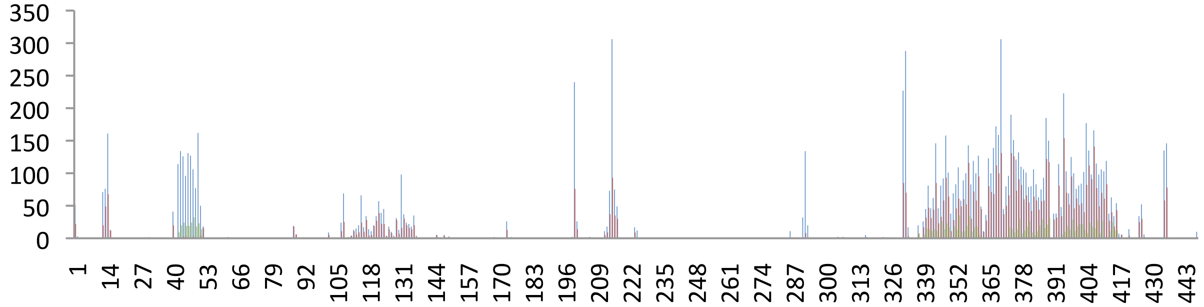
350
300
250
200
150
100
50
0
1 14 27 40 53 66 79 92 105 118 131 144 157 170 183 196 209 222 235 248 261 274 287 300 313 326 339 352 365 378 391 404 417 430 443

C)

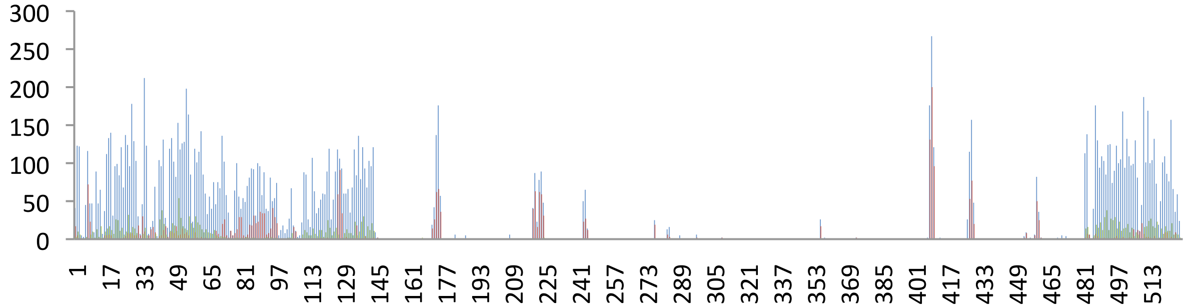
300
250
200
150
100
50
0
1 17 33 49 65 81 97 113 129 145 161 177 193 209 225 241 257 273 289 305 321 337 353 369 385 401 417 433 449 465 481 497 513

D)

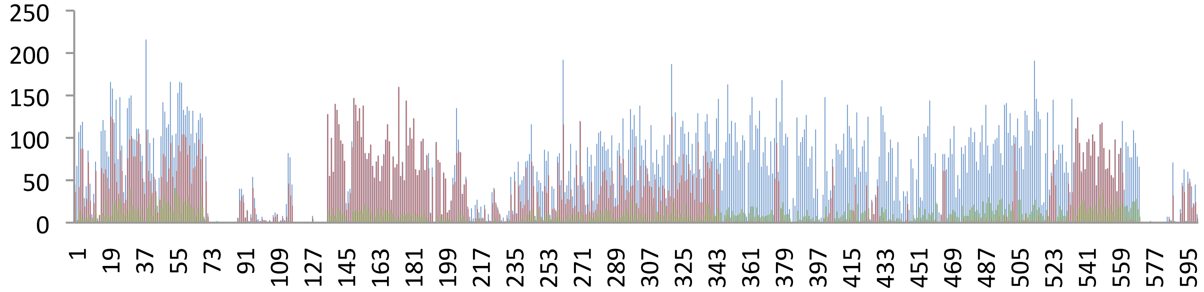
250
200
150
100
50
0
1 19 37 55 73 91 109 127 145 163 181 199 217 235 253 271 289 307 325 343 361 379 397 415 433 451 469 487 505 523 541 559 577 595

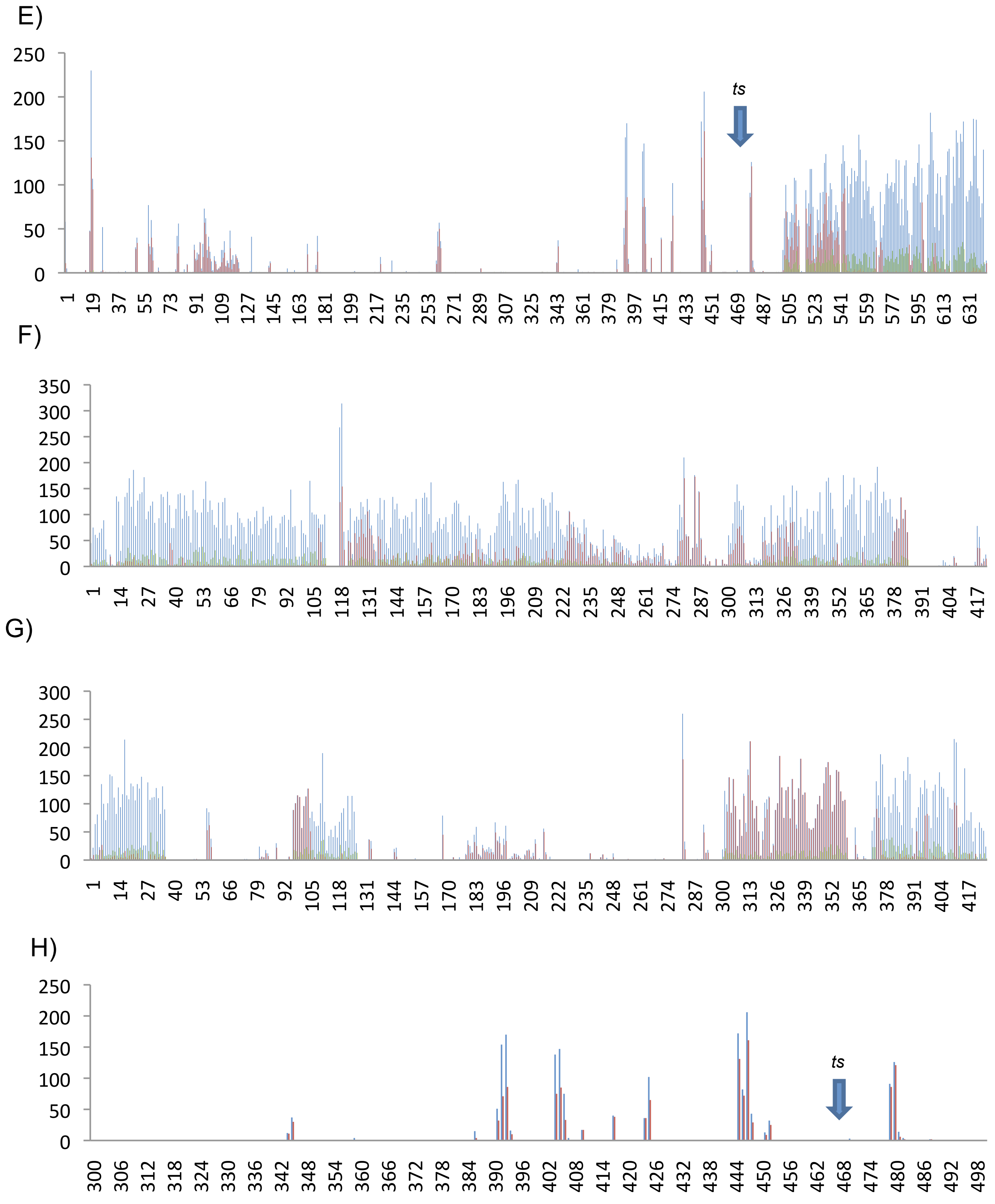

Figure S16 Distribution of polymorphisms in strain 821. A) Supercontig 1, B) Supercontig 2, C) Supercontig 3, D) Supercontig 4, E) Supercontig 5. The location of *ts* is indicated by a vertical arrow, F) Supercontig 6, G) Supercontig 7, H) The region from 3,000000 to 5000000 on Supercontig 5. The location of *ts* is indicated by a vertical arrow.Total SNPs are plotted in blue. SNPs that are unique to strain 821 are plotted in red. Indels are plotted in green. Polymorphisms were sorted by Supercontig and position and the total number in a 10 kb moving window is plotted on the Y axis. The X axis corresponds to the position along the Supercontig. Values are X 10 kb

A)

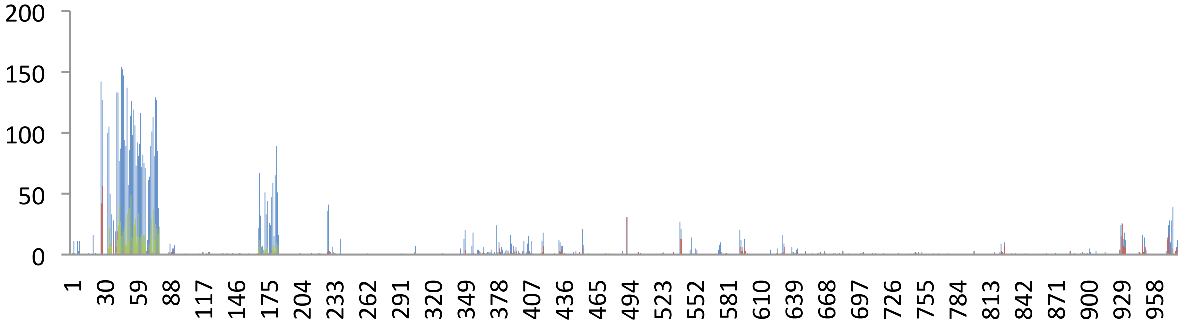
200
150
100
50
0
1 30 59 88 117 146 175 204 233 262 291 320 349 378 407 436 465 494 523 552 581 610 639 668 697 726 755 784 813 842 871 900 929 958

B)

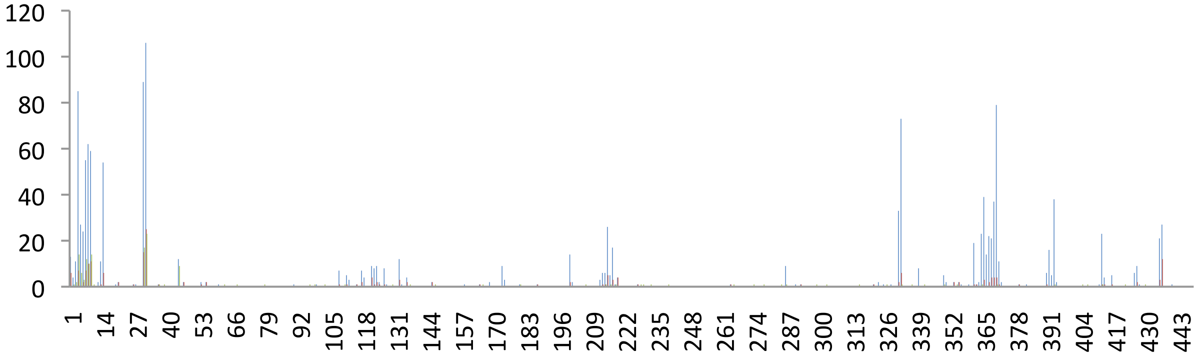
120
100
80
60
40
20
0
1 14 27 40 53 66 79 92 105 118 131 144 157 170 183 196 209 222 235 248 261 274 287 300 313 326 339 352 365 378 391 404 417 430 443

C)

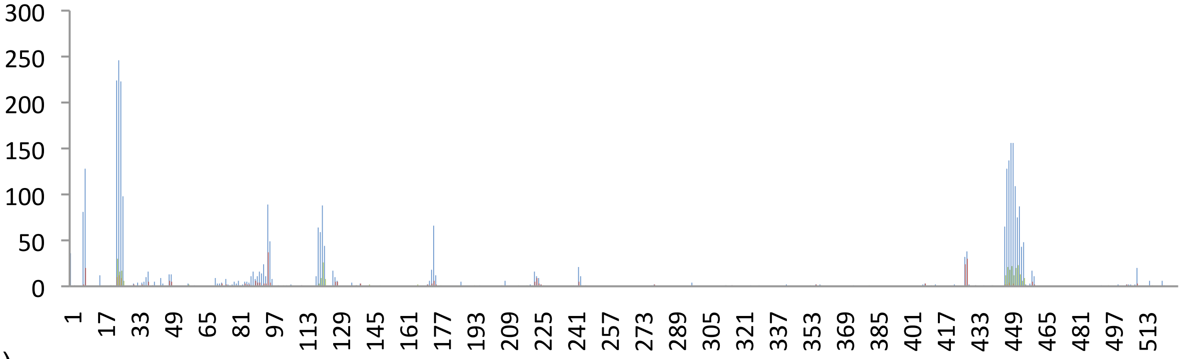
300
250
200
150
100
50
0
1 17 33 49 65 81 97 113 129 145 161 177 193 209 225 241 257 273 289 305 321 337 353 369 385 401 417 433 449 465 481 497 513

D)

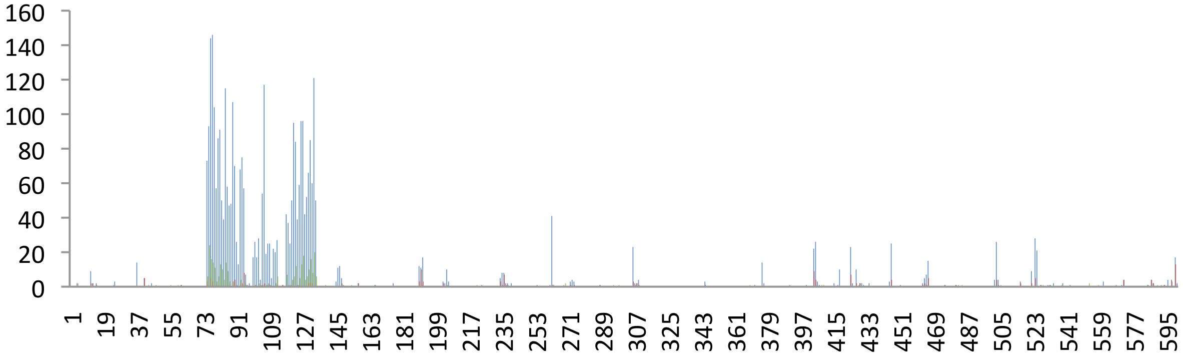
160
140
120
100
80
60
40
20
0
1 19 37 55 73 91 109 127 145 163 181 199 217 235 253 271 289 307 325 343 361 379 397 415 433 451 469 487 505 523 541 559 577 595

E)

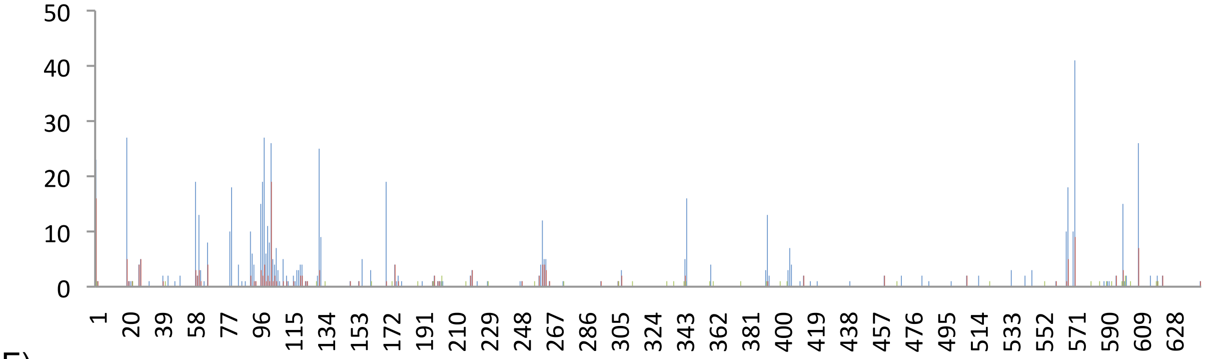

F)

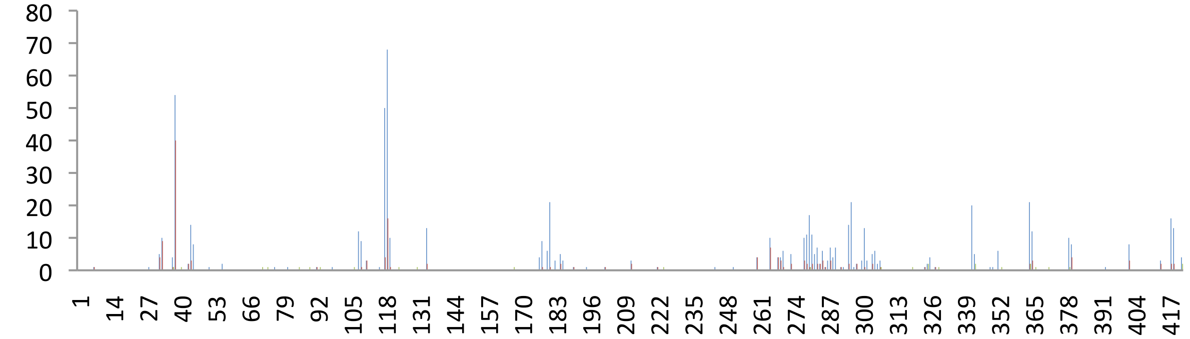

G)

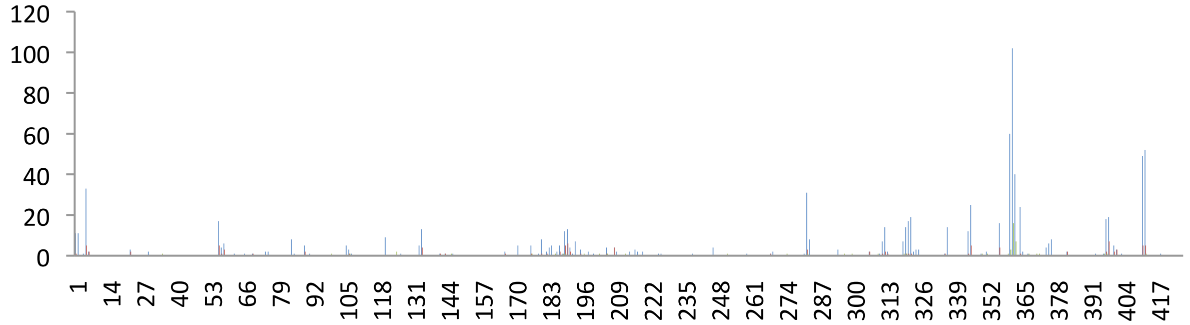

Figure S17 Distribution of polymorphisms in strain 7035. A) Supercontig 1, B) Supercontig 2, C) Supercontig 3, D) Supercontig 4, E) Supercontig 5, F) Supercontig 6, G) Supercontig 7. Total SNPs are plotted in blue. SNPs that are unique to strain 7035 are plotted in red. Indels are plotted in green. Polymorphisms were sorted by Supercontig and position and the total number in a 10 kb moving window is plotted on the Y axis. The X axis corresponds to the position along the Supercontig (X 10 kb).

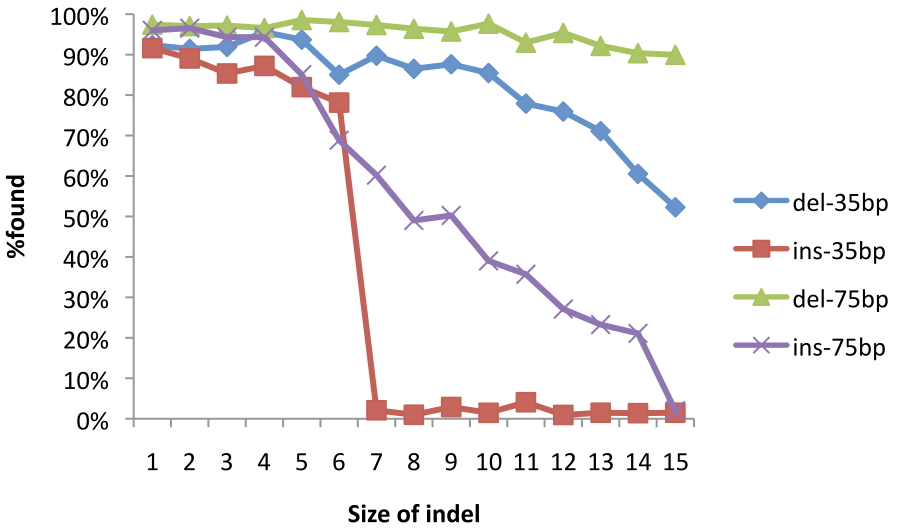

Figure S18 Detection of simulated insertions and deletions in whole genome sequence of Neurospora.
To determine the effect of read length on the ability to identify small indels, we altered the reference of supercont10.1 at evenly spaced intervals to simulate indels, aligned un-altered data to the altered reference, and then determined if maq was able to correctly identify the simulated indels. In general insertions are harder to identify than deletions, and longer reads allow more indels to be identified.

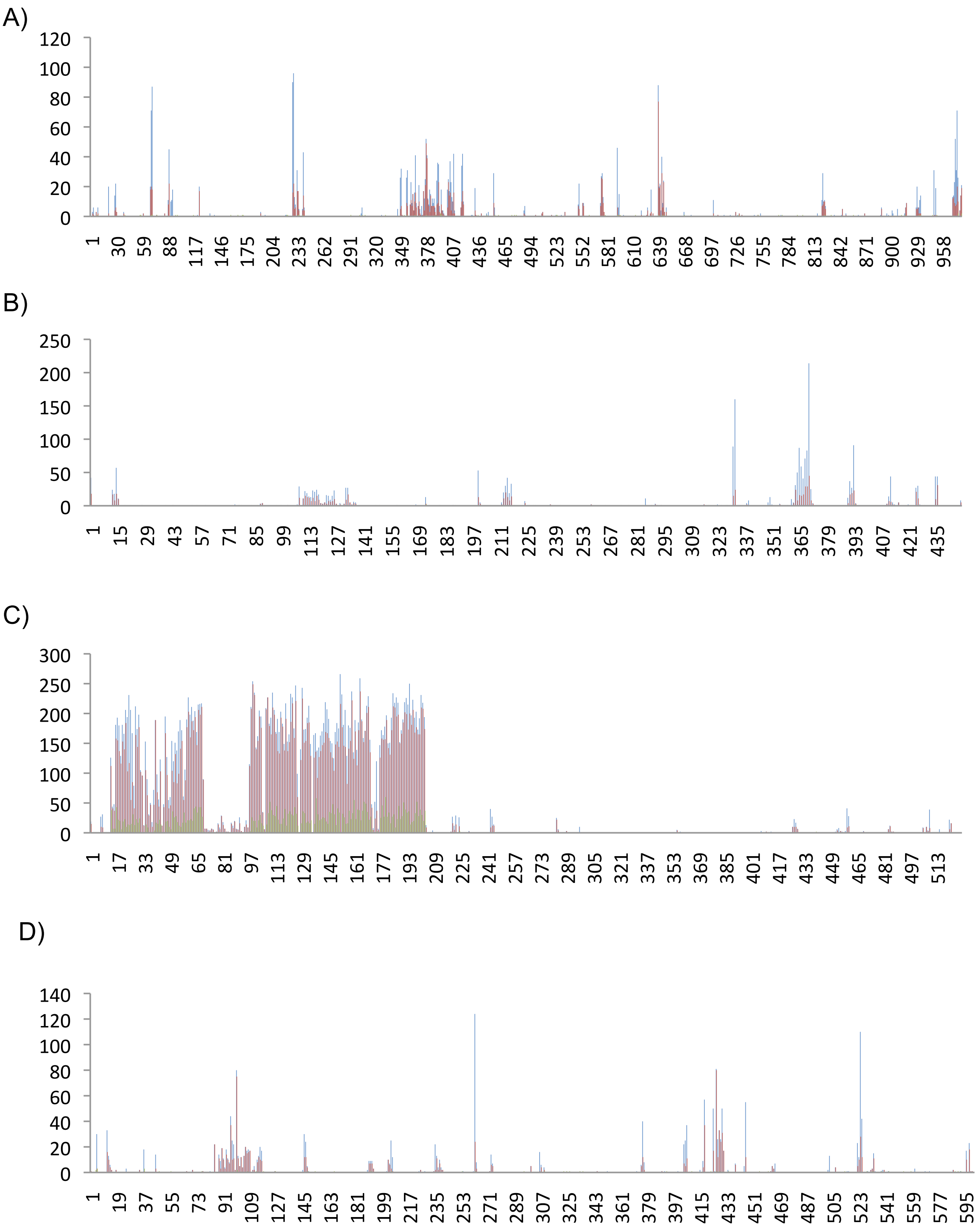
A)
0 20 40 60 80 100 120
1 30 59 88 117 146 175 204 233 262 291 320 349 378 407 436 465 494 523 552 581 610 639 668 697 726 755 784 813 842 871 900 929 958
B)
0 50 100 150 200 250
1 15 29 43 57 71 85 99 113 127 141 155 169 183 197 211 225 239 253 267 281 295 309 323 337 351 365 379 393 407 421 435
C)
0 50 100 150 200 250 300
1 17 33 49 65 81 97 113 129 145 161 177 193 209 225 241 257 273 289 305 321 337 353 369 385 401 417 433 449 465 481 497 513
D)
0 20 40 60 80 100 120 140
1 19 37 55 73 91 109 127 145 163 181 199 217 235 253 271 289 307 325 343 361 379 397 415 433 451 469 487 505 523 541 559 577 595

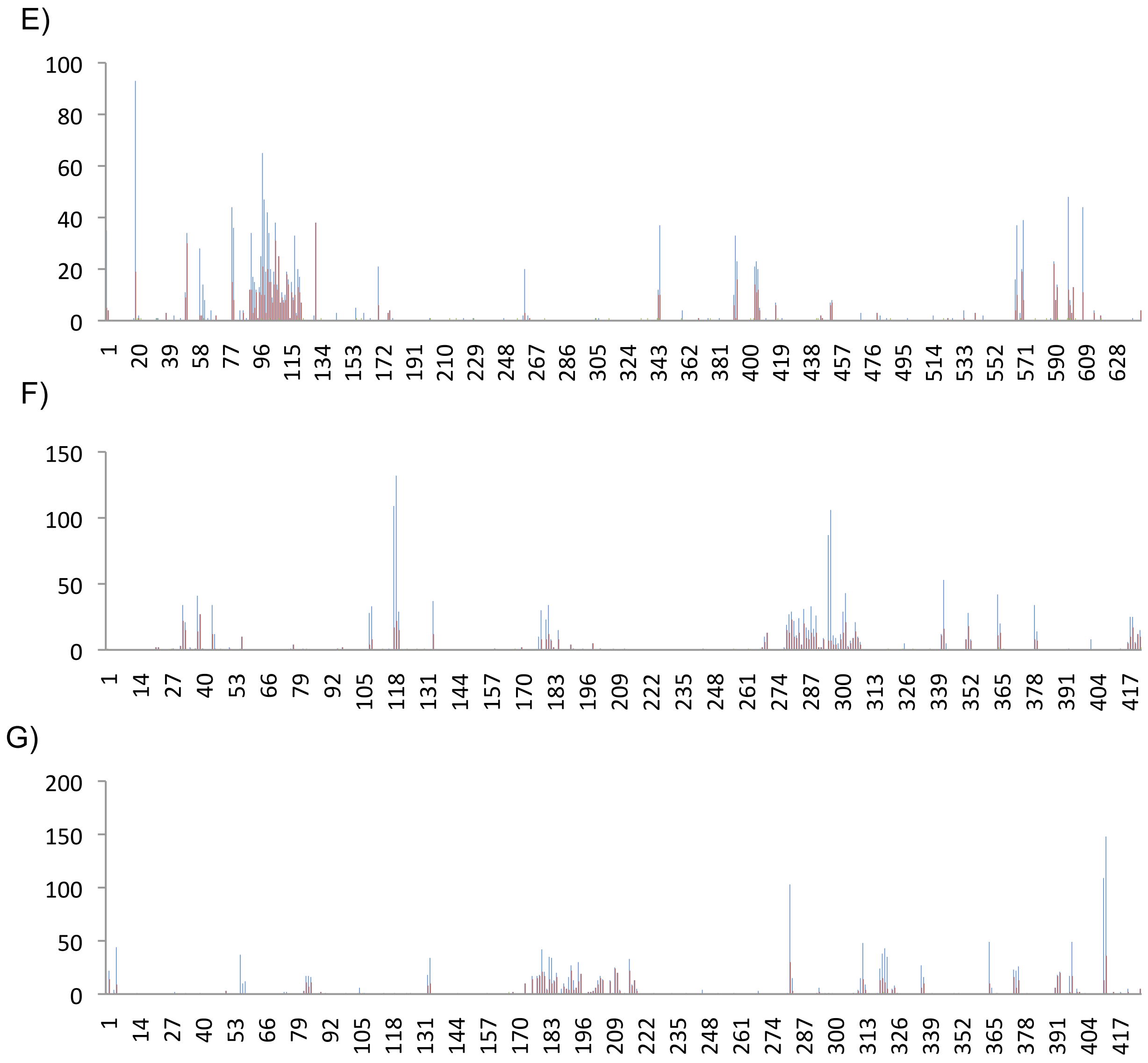

Figure S19 Distribution of polymorphisms in strain 3114. A) Supercontig 1, B) Supercontig 2, C) Supercontig 3, D) Supercontig 4, E) Supercontig 5, F) Supercontig 6, G) Supercontig 7. Total SNPs are plotted in blue. SNPs that are unique to strain 3114 are plotted in red. Indels are plotted in green. Polymorphisms were sorted by Supercontig and position and the total number in a 10 kb moving window is plotted on the Y axis. The X axis corresponds to the position along the Supercontig.

File S1

Supporting Data

File S1 is available for a download at http://www.g3journal.org/lookup/suppl/doi:10.1534/g3.111.000307/-/DC1/FileS1.zip as a compressed folder.
