# Supplementary material for: Rediscovery by Whole Genome Sequencing: Classical Mutations and Genome Polymorphisms in Neurospora crassa
Source: G3 (Bethesda). 2011 Sep 1;1(4):303–16. doi: 10.1534/g3.111.000307 (PMC3276140; doi:10.1534/g3.111.000307)
Supplement: Supporting Information [file supp_1.4.303_FigureS2.pdf]

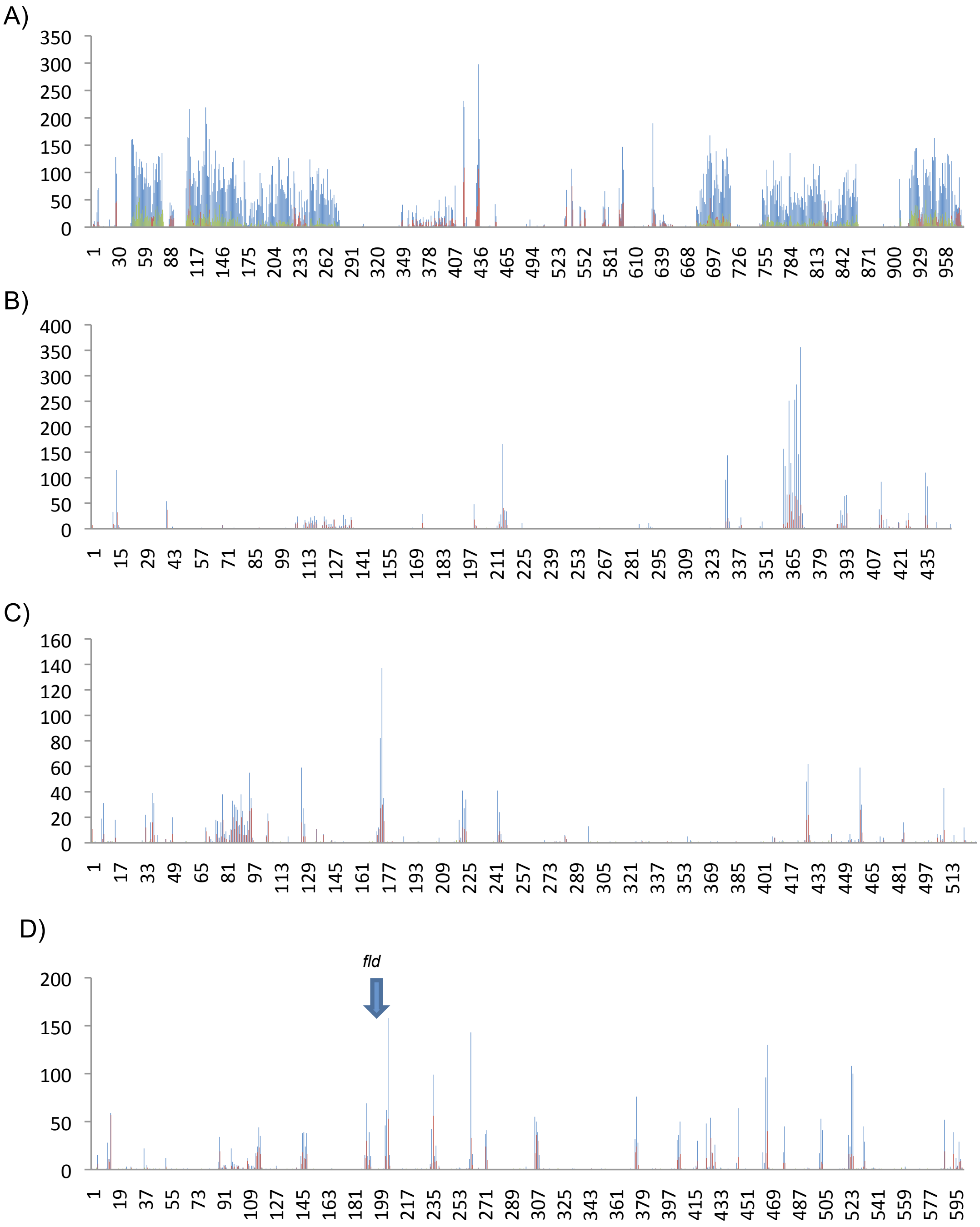
A)
B)
C)
D)
fld

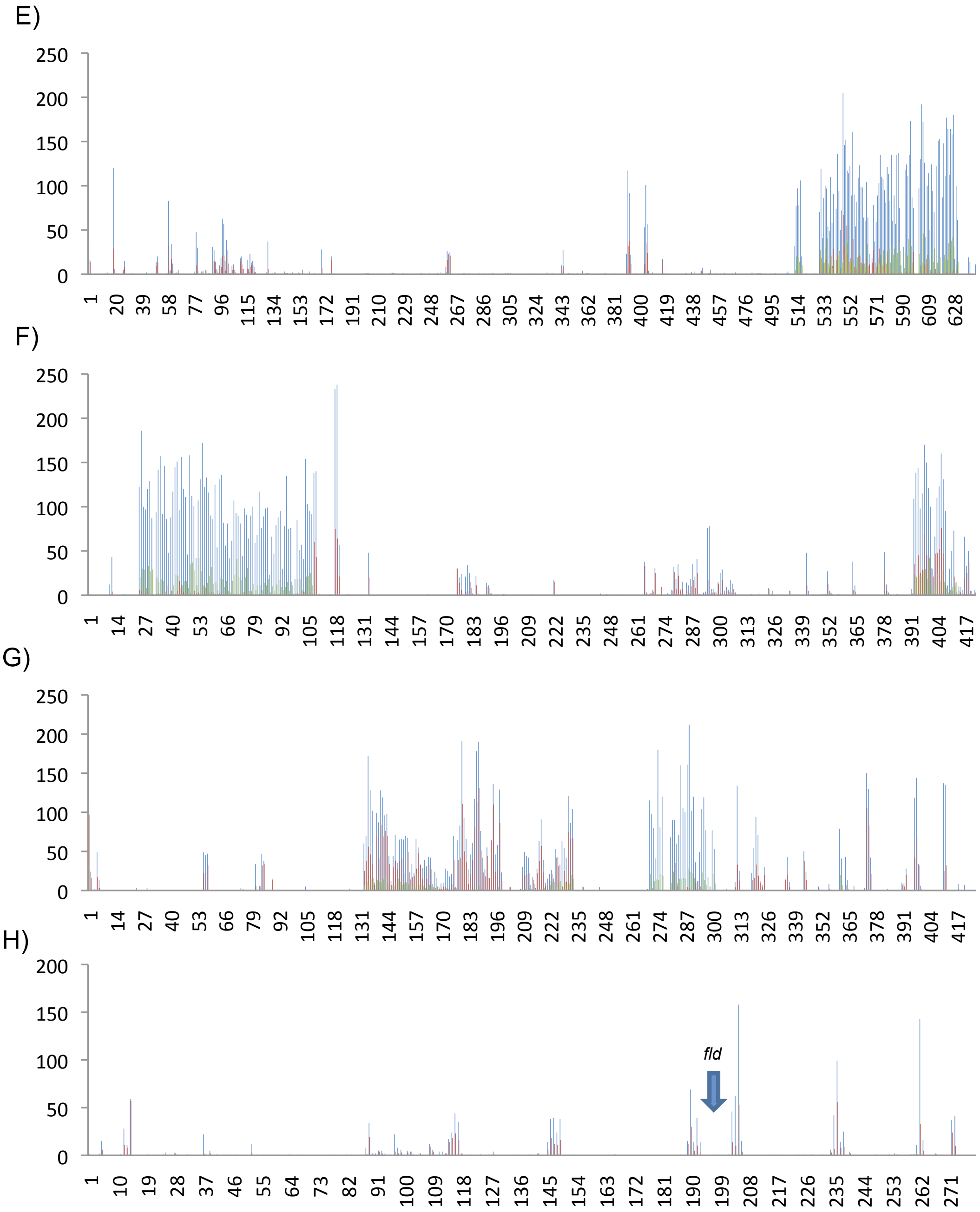

Figure S2 Distribution of polymorphisms in strain 7022. A) Supercontig 1, B) Supercontig 2, C) Supercontig 3, D) Supercontig 4. The location of *fld* is indicated by a vertical arrow, E) Supercontig 5, F) Supercontig 6, G) Supercontig 7, H) The region to the left of *his-5* on Supercontig 4. The location of *fld* is shown by a vertical arrow. Total SNPs are plotted in blue. SNPs that are unique to strain 7022 are plotted in red. Indels are plotted in green. Polymorphisms were sorted by Supercontig and position and the total number in a 10 kb moving window is plotted on the Y axis. The X axis corresponds to the position along the Supercontig (X 10 kb).
