# Supplementary material for: Rediscovery by Whole Genome Sequencing: Classical Mutations and Genome Polymorphisms in Neurospora crassa
Source: G3 (Bethesda). 2011 Sep 1;1(4):303–16. doi: 10.1534/g3.111.000307 (PMC3276140; doi:10.1534/g3.111.000307)
Supplement: Supporting Information [file supp_1.4.303_FigureS6.pdf]

A)

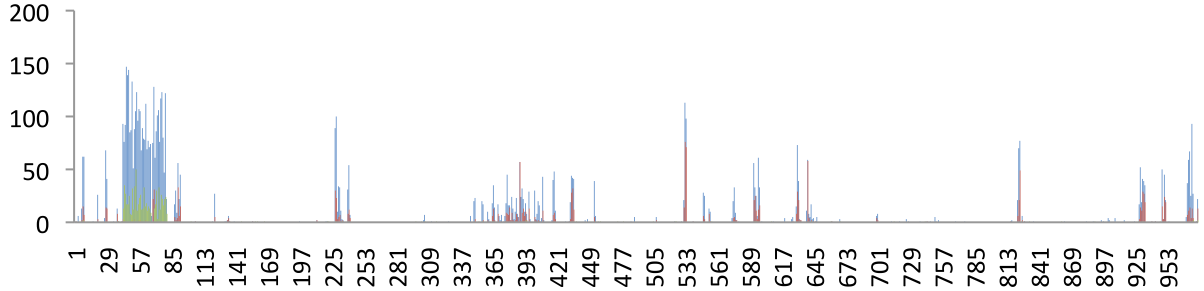
200
150
100
50
0
1 29 57 85 113 141 169 197 225 253 281 309 337 365 393 421 449 477 505 533 561 589 617 645 673 701 729 757 785 813 841 869 897 925 953

B)

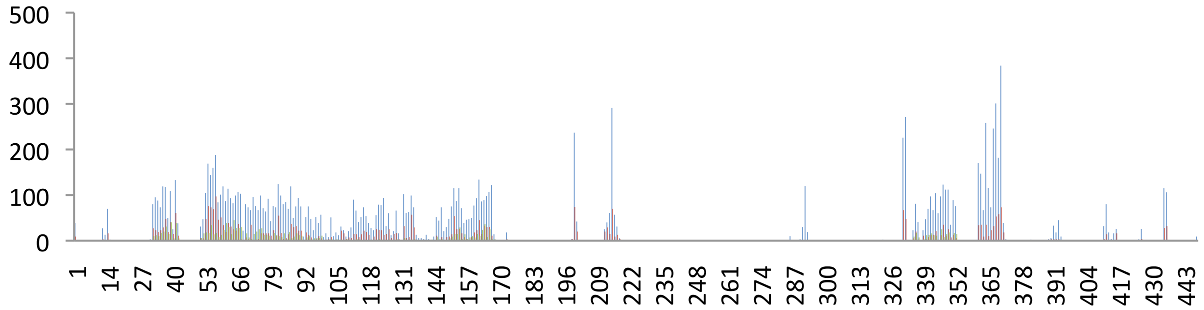
500
400
300
200
100
0
1 14 27 40 53 66 79 92 105 118 131 144 157 170 183 196 209 222 235 248 261 274 287 300 313 326 339 352 365 378 391 404 417 430 443

C)

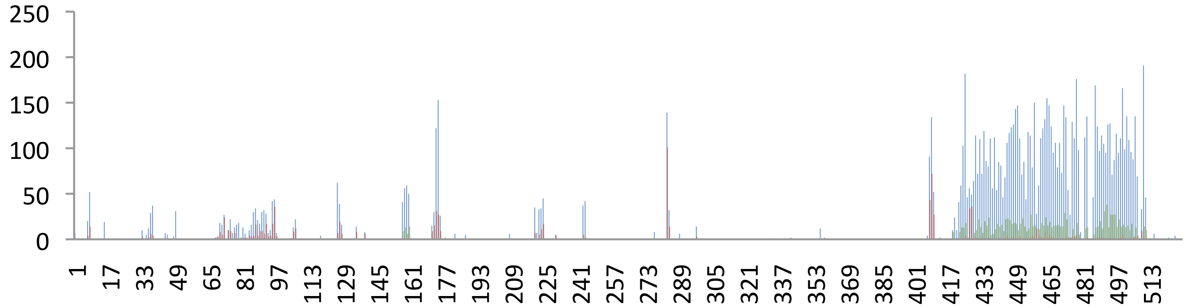
250
200
150
100
50
0
1 17 33 49 65 81 97 113 129 145 161 177 193 209 225 241 257 273 289 305 321 337 353 369 385 401 417 433 449 465 481 497 513

D)

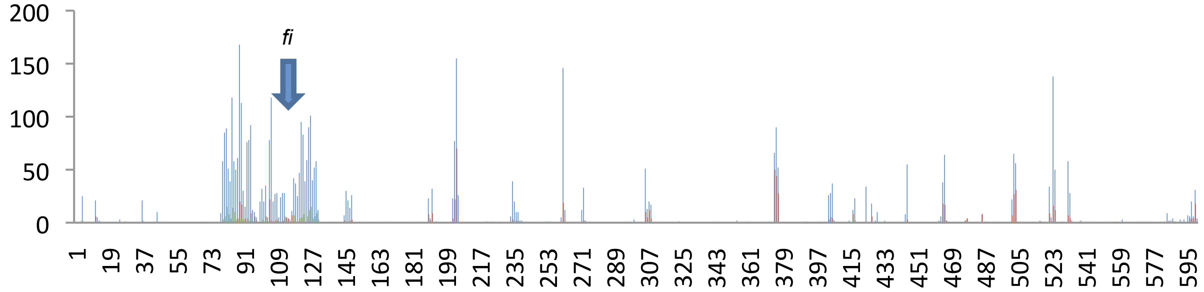
200
150
100
50
0
fi
1 19 37 55 73 91 109 127 145 163 181 199 217 235 253 271 289 307 325 343 361 379 397 415 433 451 469 487 505 523 541 559 577 595

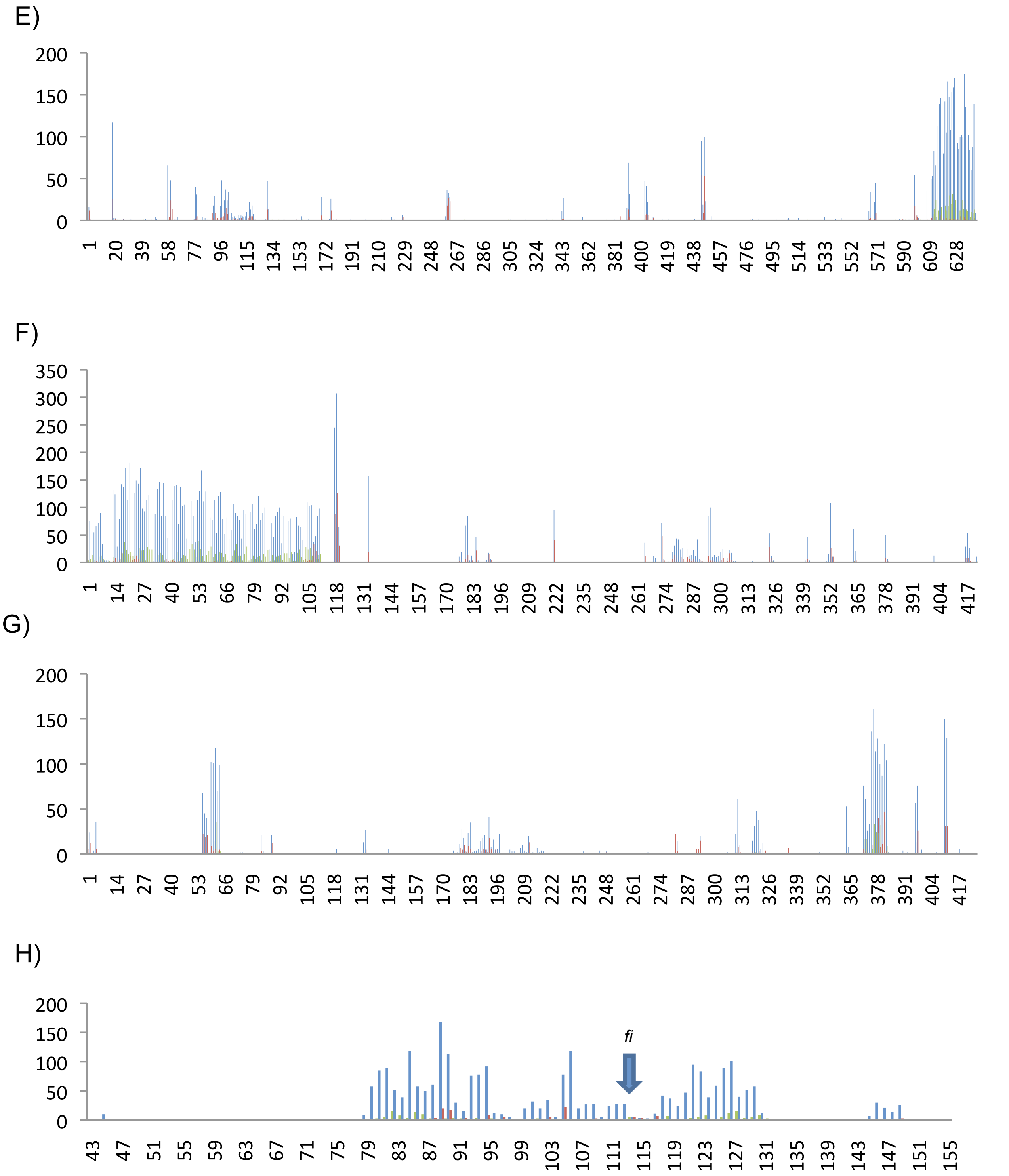

Figure S6 Distribution of polymorphisms in strain 1303. A) Supercontig 1, B) Supercontig 2, C) Supercontig 3, D) Supercontig 4. The location of *fi* is indicated by a vertical arrow, E) Supercontig 5, F) Supercontig 6, G) Supercontig 7, H) The region from 430000 to 1550000 on Supercontig 4. The location of *fi* is indicated by a vertical arrow. Total SNPs are plotted in blue. SNPs that are unique to strain 1303 are plotted in red. Indels are plotted in green. Polymorphisms were sorted by Supercontig and position and the total number in a 10 kb moving window is plotted on the Y axis. The X axis corresponds to the position along the Supercontig. (X 10 kb)
