# Supplementary material for: Rediscovery by Whole Genome Sequencing: Classical Mutations and Genome Polymorphisms in Neurospora crassa
Source: G3 (Bethesda). 2011 Sep 1;1(4):303–16. doi: 10.1534/g3.111.000307 (PMC3276140; doi:10.1534/g3.111.000307)
Supplement: Supporting Information [file supp_1.4.303_FigureS10.pdf]

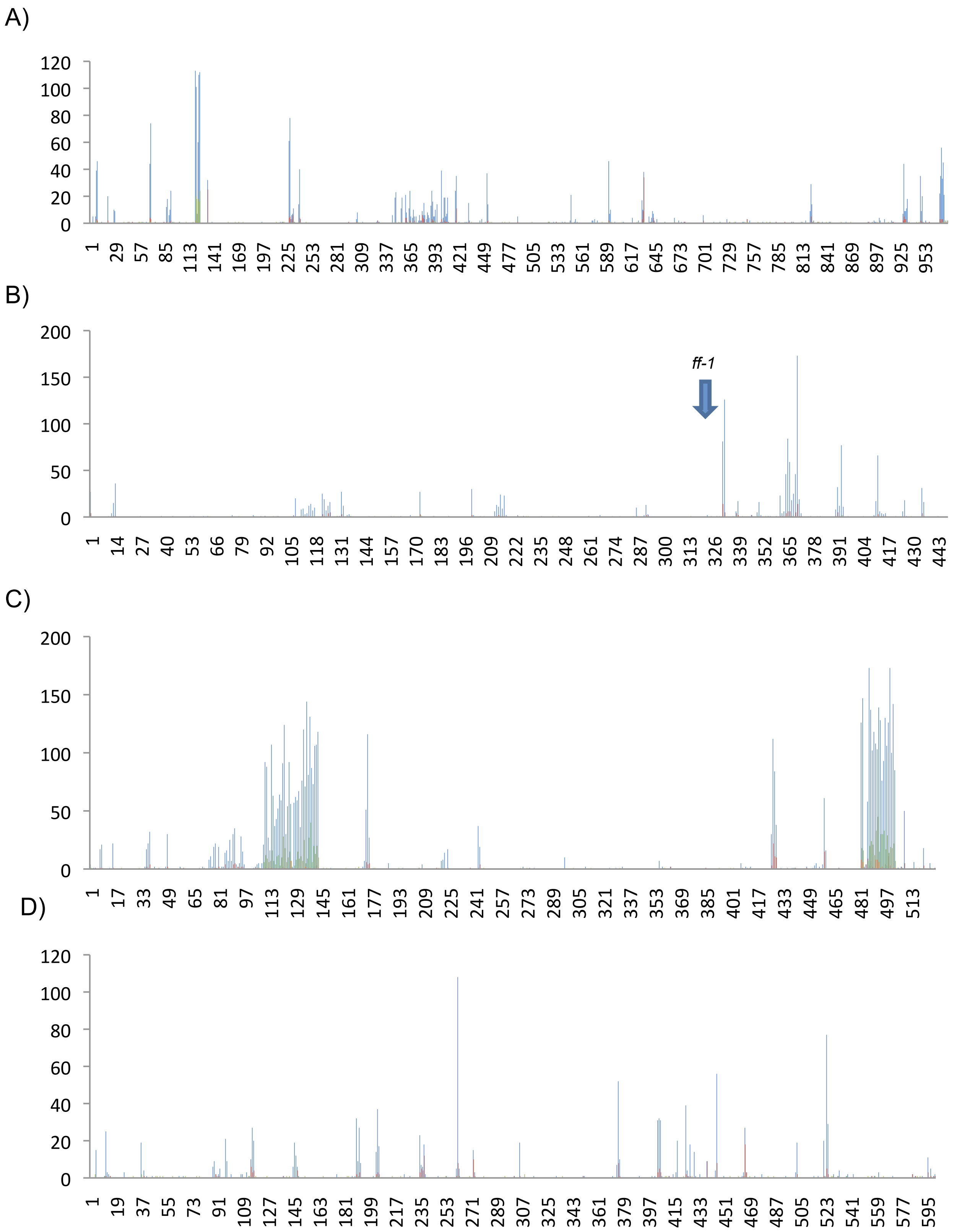
A)
120
100
80
60
40
20
0
B)
ff-1
200
150
100
50
0
C)
200
150
100
50
0
D)
120
100
80
60
40
20
0

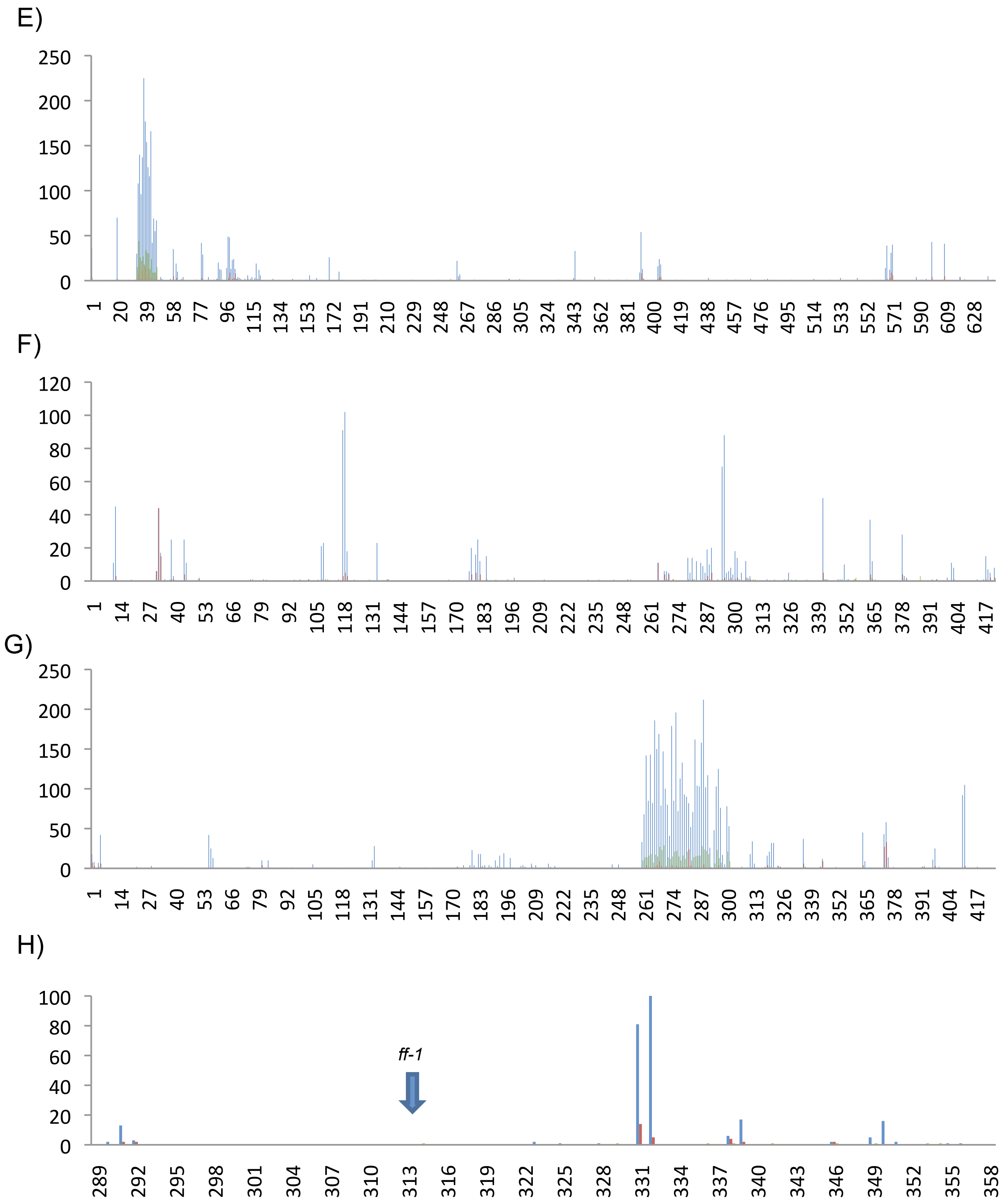

Figure S10 Distribution of polymorphisms in strain 3831. A) Supercontig 1, B) Supercontig 2. The location of *ff-1*is indicated by a vertical arrow, C) Supercontig 3, D) Supercontig 4, E) Supercontig 5, F) Supercontig 6, G) Supercontig 7, H) The region from 2897659 to 3571946 on Supercontig 2. The location of *ff-1* is indicated by a vertical arrow. Total SNPs are plotted in blue. SNPs that are unique to strain 3831 are plotted in red. Indels are plotted in green. Polymorphisms were sorted by Supercontig and position and the total number in a 10 kb moving window is plotted on the Y axis. The X axis corresponds to the position along the Supercontig. (X 10 kb)
