# Supplementary material for: Rediscovery by Whole Genome Sequencing: Classical Mutations and Genome Polymorphisms in Neurospora crassa
Source: G3 (Bethesda). 2011 Sep 1;1(4):303–16. doi: 10.1534/g3.111.000307 (PMC3276140; doi:10.1534/g3.111.000307)
Supplement: Supporting Information [file supp_1.4.303_FigureS12.pdf]

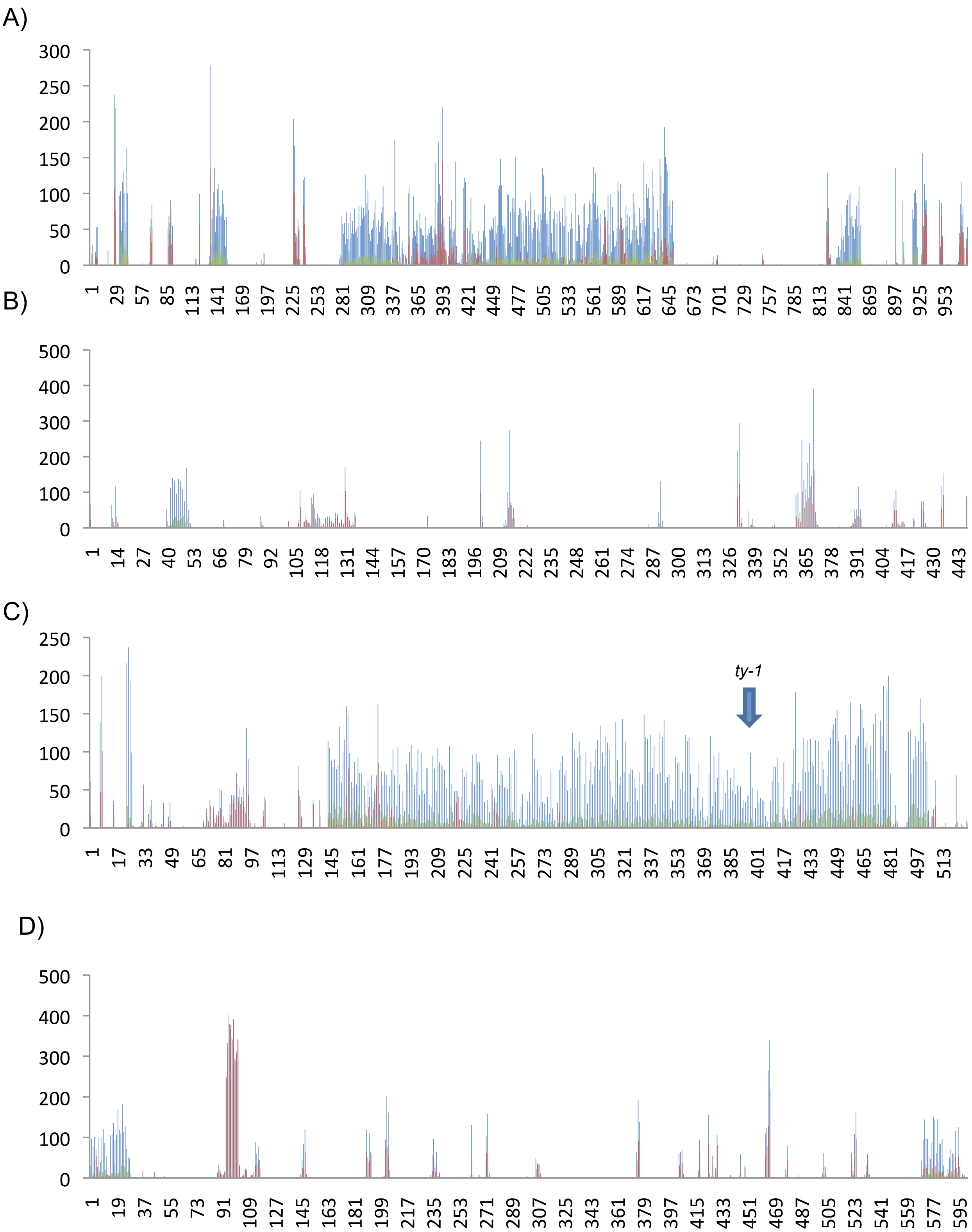
A)
B)
C)
ty-1
D)

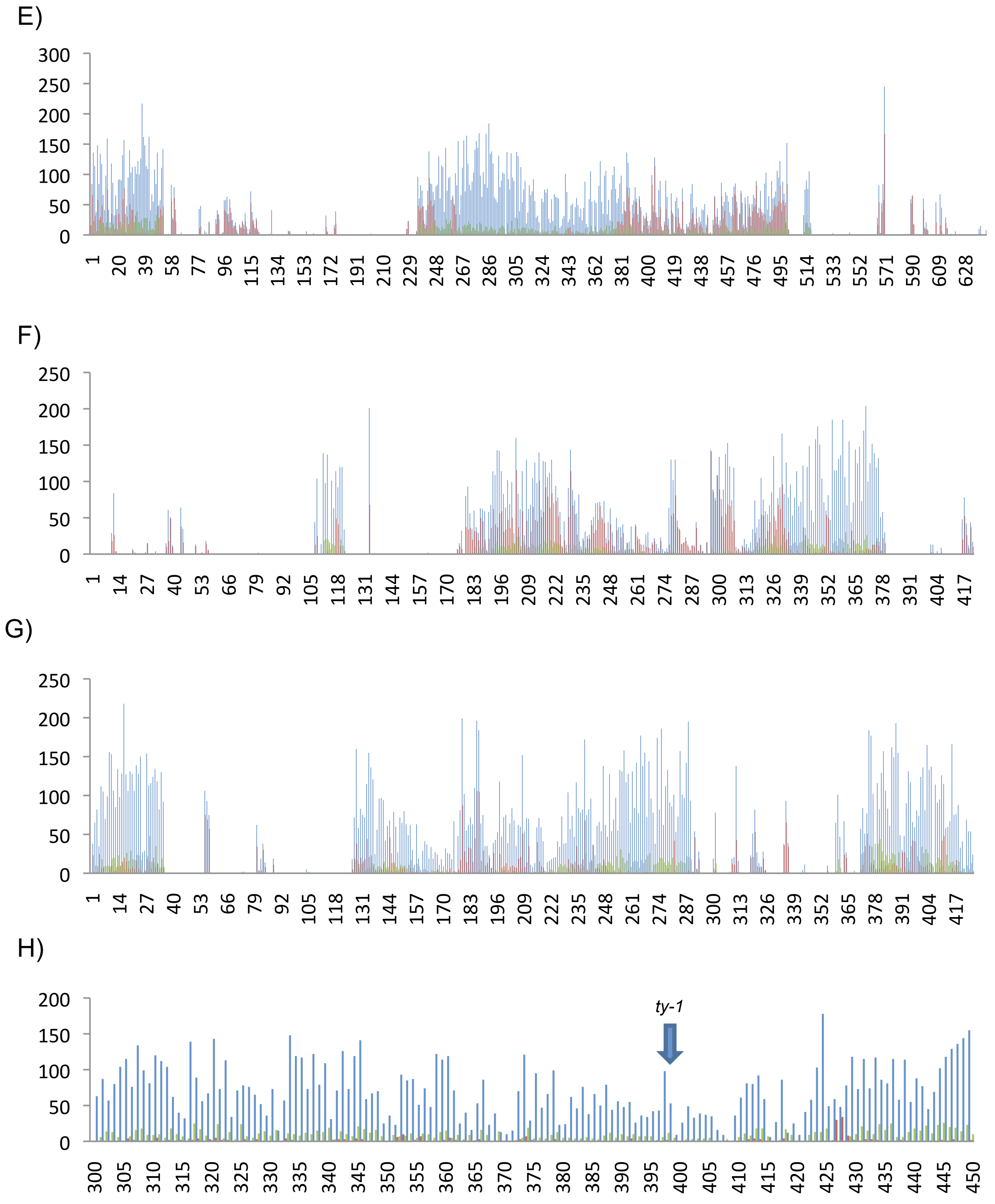

Figure S12 Distribution of polymorphisms in strain 322. A) Supercontig 1, B) Supercontig 2, C) Supercontig 3. The location of *ty-1* is indicated by a vertical arrow. , D) Supercontig 4, E) Supercontig 5, F) Supercontig 6, G) Supercontig 7, H) The region from 3000000 to 4500000 on Supercontig 3. The location of *ty-1* is indicated by a vertical arrow. Total SNPs are plotted in blue. SNPs that are unique to strain 322 are plotted in red. Indels are plotted in green. Polymorphisms were sorted by Supercontig and position and the total number in a 10 kb moving window is plotted on the Y axis. The X axis corresponds to the position along the Supercontig.
