# Supplementary material for: Rediscovery by Whole Genome Sequencing: Classical Mutations and Genome Polymorphisms in Neurospora crassa
Source: G3 (Bethesda). 2011 Sep 1;1(4):303–16. doi: 10.1534/g3.111.000307 (PMC3276140; doi:10.1534/g3.111.000307)
Supplement: Supporting Information [file supp_1.4.303_FigureS13.pdf]

A)

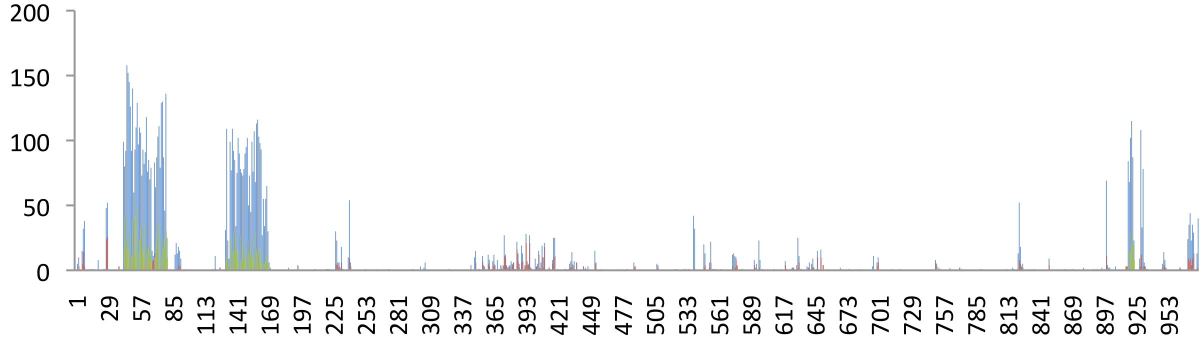
200
150
100
50
0
1
29
57
85
113
141
169
197
225
253
281
309
337
365
393
421
449
477
505
533
561
589
617
645
673
701
729
757
785
813
841
869
897
925
953

B)

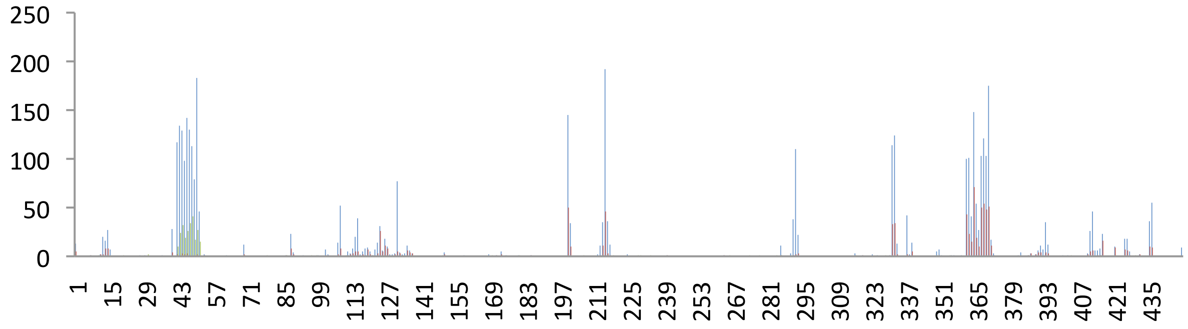
250
200
150
100
50
0
1
15
29
43
57
71
85
99
113
127
141
155
169
183
197
211
225
239
253
267
281
295
309
323
337
351
365
379
393
407
421
435

C)

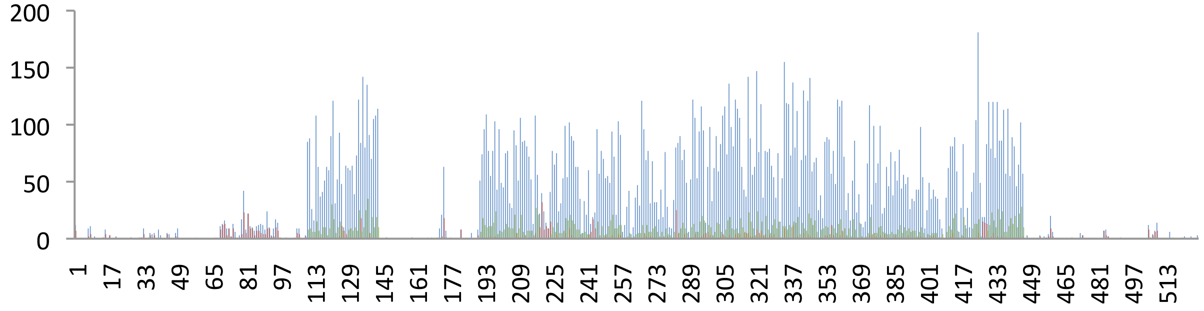
200
150
100
50
0
1
17
33
49
65
81
97
113
129
145
161
177
193
209
225
241
257
273
289
305
321
337
353
369
385
401
417
433
449
465
481
497
513

D)

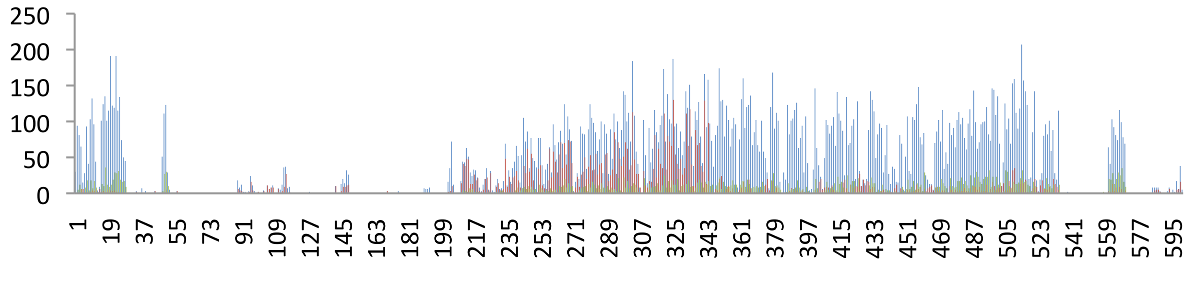
250
200
150
100
50
0
1
19
37
55
73
91
109
127
145
163
181
199
217
235
253
271
289
307
325
343
361
379
397
415
433
451
469
487
505
523
541
559
577
595

E)

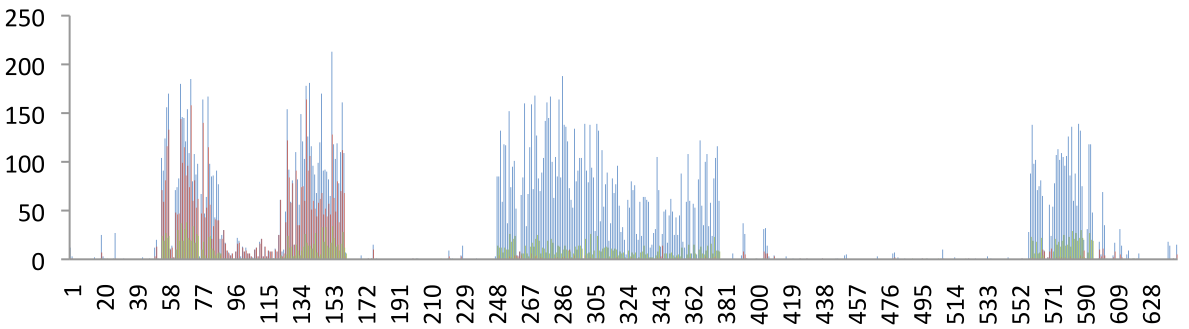

F)

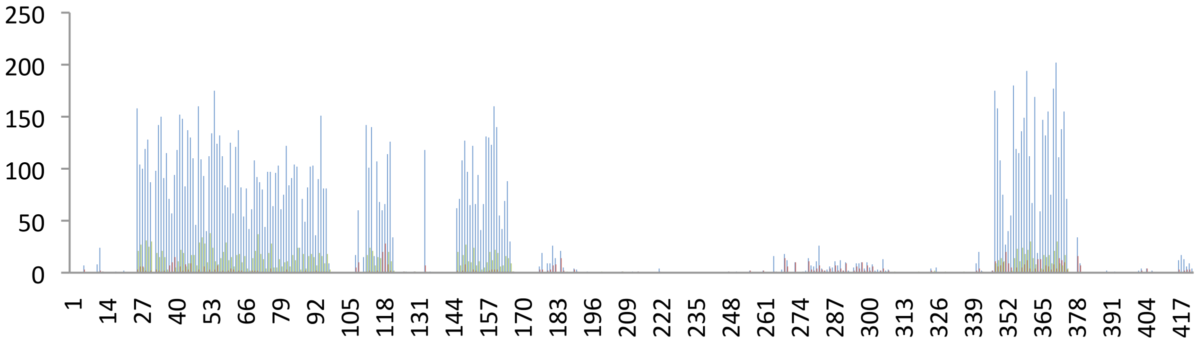

G)

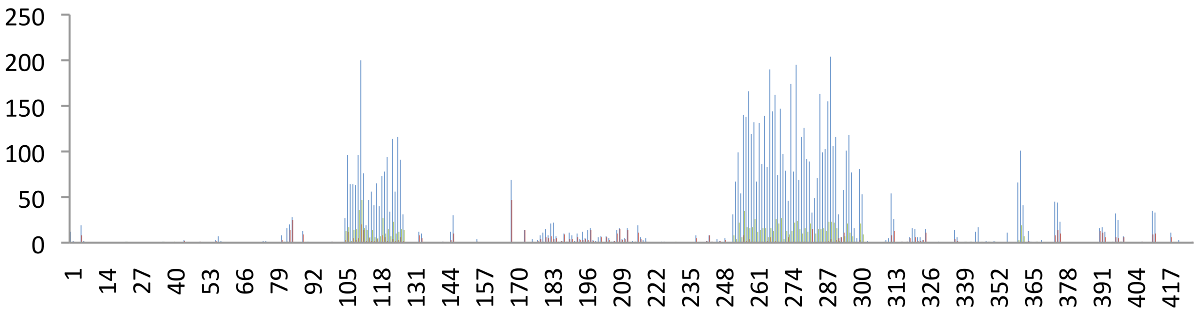

Figure S13 Distribution of polymorphisms in strain 3562. A) Supercontig 1, B) Supercontig 2, C) Supercontig 3, D) Supercontig 4, E) Supercontig 5, F) Supercontig 6, G) Supercontig 7. Total SNPs are plotted in blue. SNPs that are unique to strain 3562 are plotted in red. Indels are plotted in green. Polymorphisms were sorted by Supercontig and position and the total number in a 10 kb moving window is plotted on the Y axis. The X axis corresponds to the position along the Supercontig. Values are X 10 kb.
