# Supplementary material for: Rediscovery by Whole Genome Sequencing: Classical Mutations and Genome Polymorphisms in Neurospora crassa
Source: G3 (Bethesda). 2011 Sep 1;1(4):303–16. doi: 10.1534/g3.111.000307 (PMC3276140; doi:10.1534/g3.111.000307)
Supplement: Supporting Information [file supp_1.4.303_FigureS14.pdf]

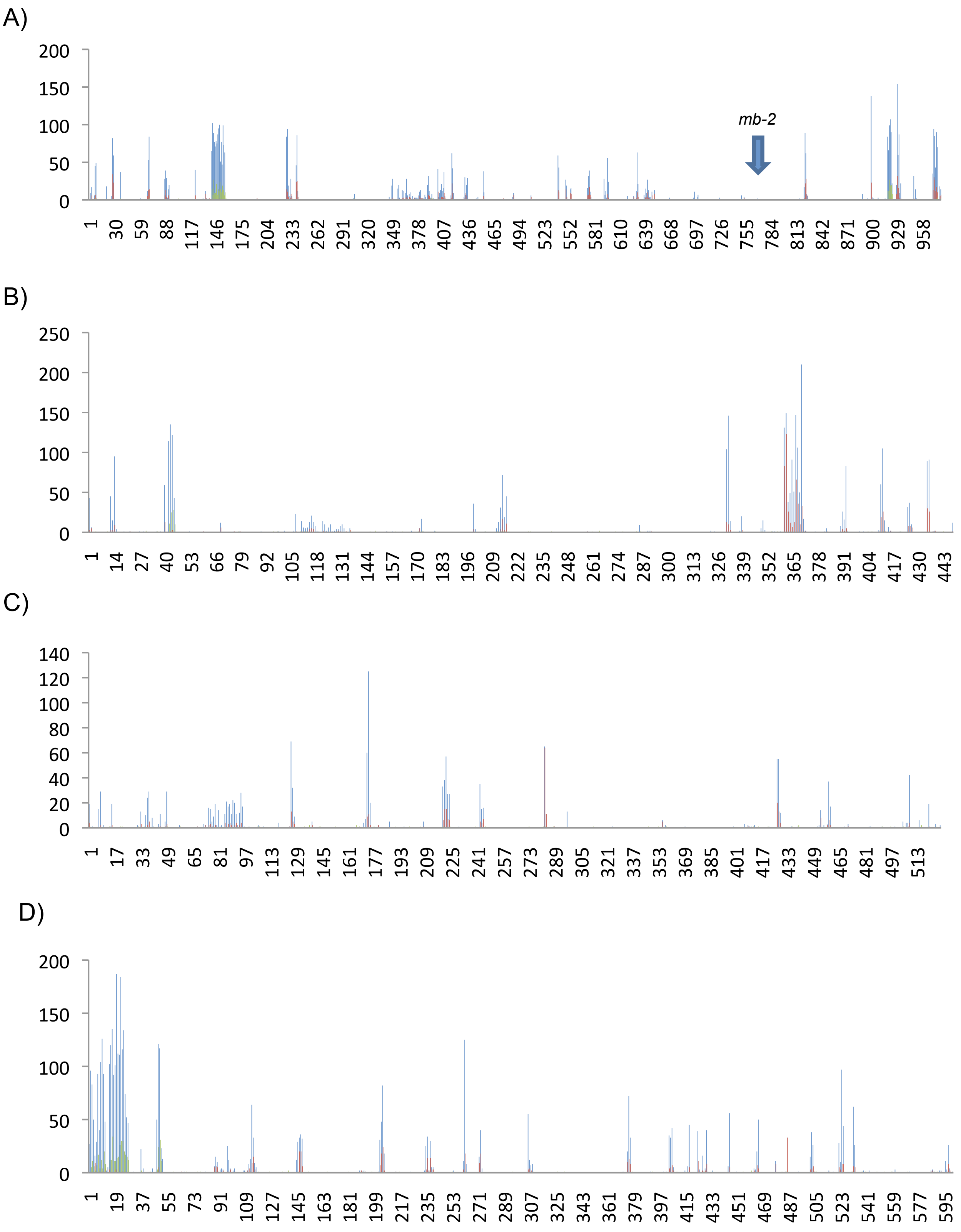
A)
mb-2
B)
C)
D)

E)

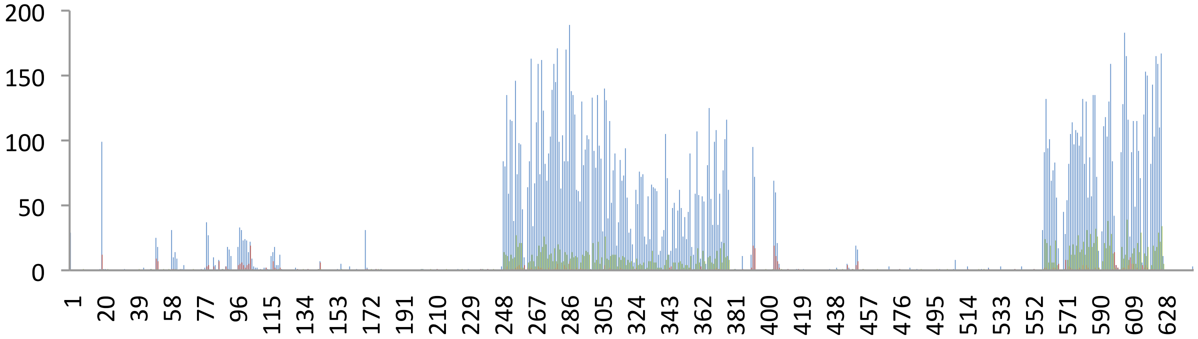


F)

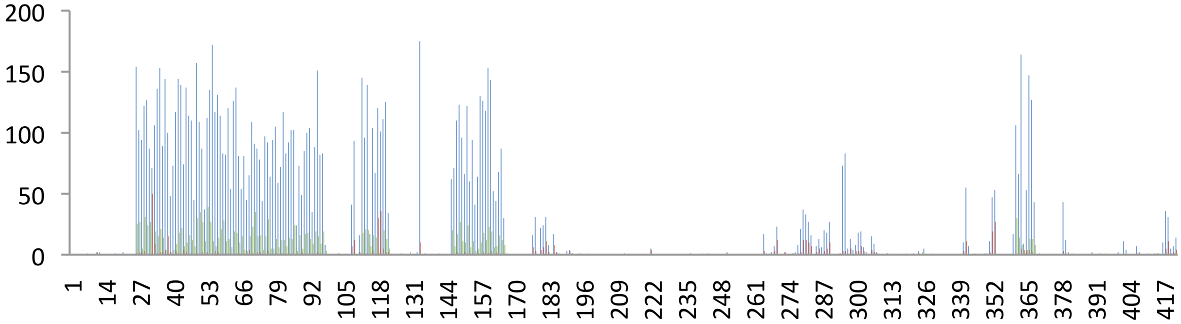


G)

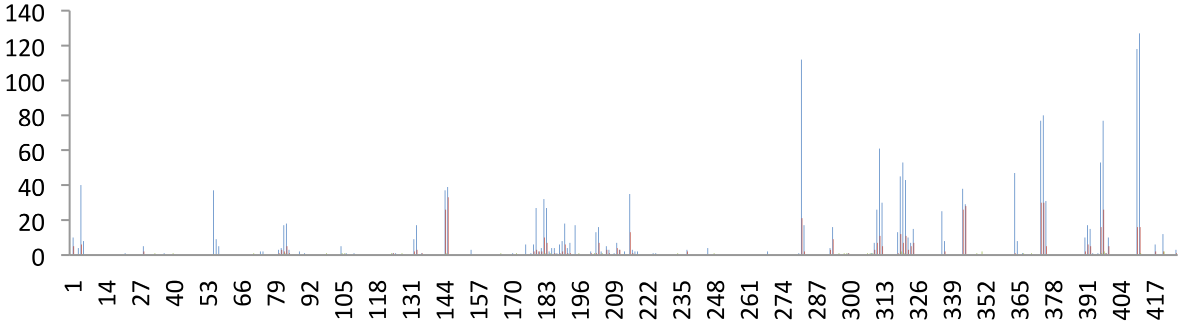


**Figure S14** Distribution of polymorphisms in strain 3564. A) Supercontig 1. The location of *mb-2* is indicated by a vertical arrow. There are only eight SNPs in the region bounded by the flanking markers in this strain, B) Supercontig 2, C) Supercontig 3, D) Supercontig 4, E) Supercontig 5, F) Supercontig 6, G) Supercontig 7. Total SNPs are plotted in blue. SNPs that are unique to strain 3564 are plotted in red. Indels are plotted in green. Polymorphisms were sorted by Supercontig and position and the total number in a 10 kb moving window is plotted on the Y axis. The X axis corresponds to the position along the Supercontig (X 10 kb).
