# Supplementary material for: Rediscovery by Whole Genome Sequencing: Classical Mutations and Genome Polymorphisms in Neurospora crassa
Source: G3 (Bethesda). 2011 Sep 1;1(4):303–16. doi: 10.1534/g3.111.000307 (PMC3276140; doi:10.1534/g3.111.000307)
Supplement: Supporting Information [file supp_1.4.303_FigureS15.pdf]

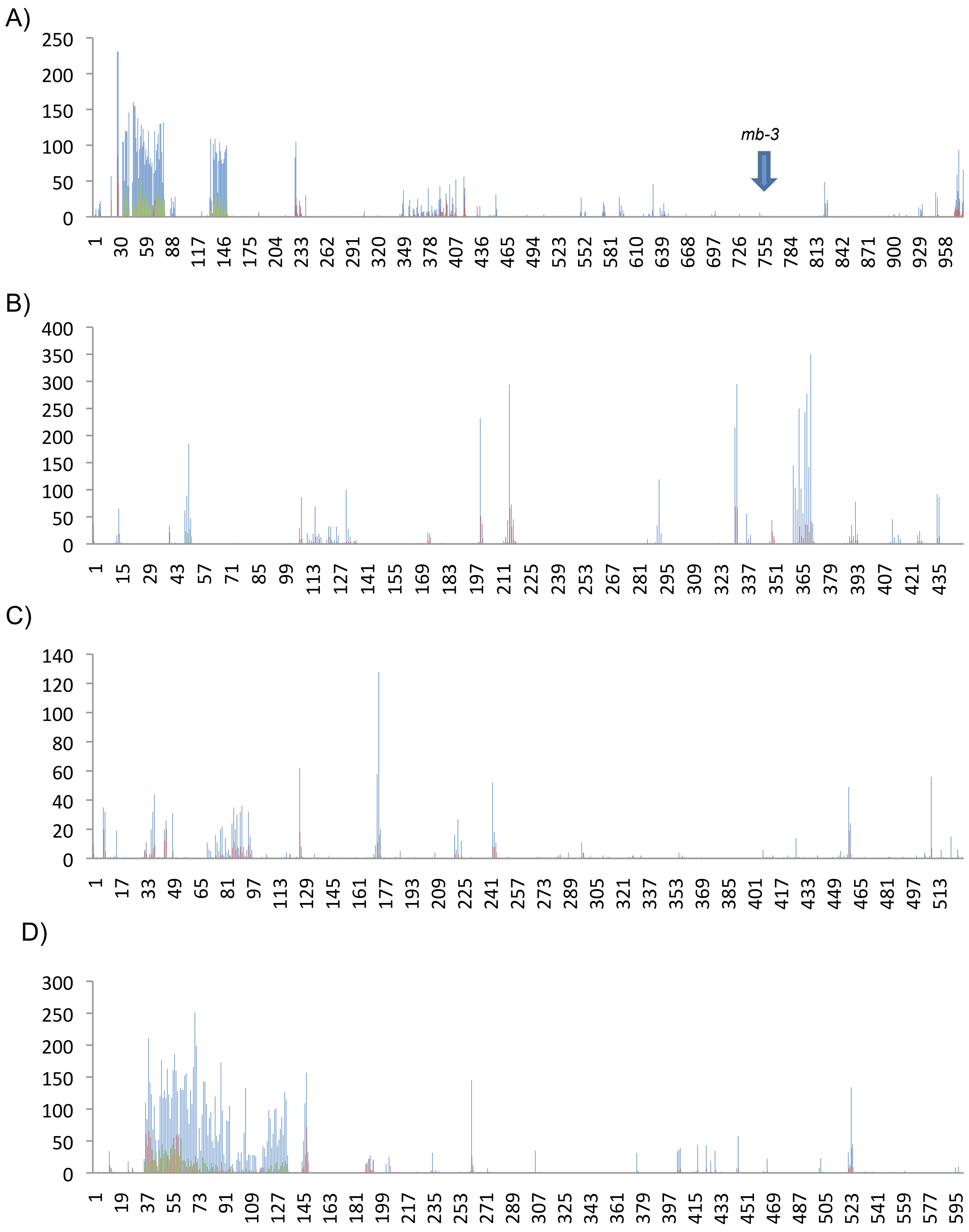

A)
250
200
150
100
50
0
1 30 59 88 117 146 175 204 233 262 291 320 349 378 407 436 465 494 523 552 581 610 639 668 697 726 755 784 813 842 871 900 929 958
mb-3
B)
400
350
300
250
200
150
100
50
0
1 15 29 43 57 71 85 99 113 127 141 155 169 183 197 211 225 239 253 267 281 295 309 323 337 351 365 379 393 407 421 435
C)
140
120
100
80
60
40
20
0
1 17 33 49 65 81 97 113 129 145 161 177 193 209 225 241 257 273 289 305 321 337 353 369 385 401 417 433 449 465 481 497 513
D)
300
250
200
150
100
50
0
1 19 37 55 73 91 109 127 145 163 181 199 217 235 253 271 289 307 325 343 361 379 397 415 433 451 469 487 505 523 541 559 577 595

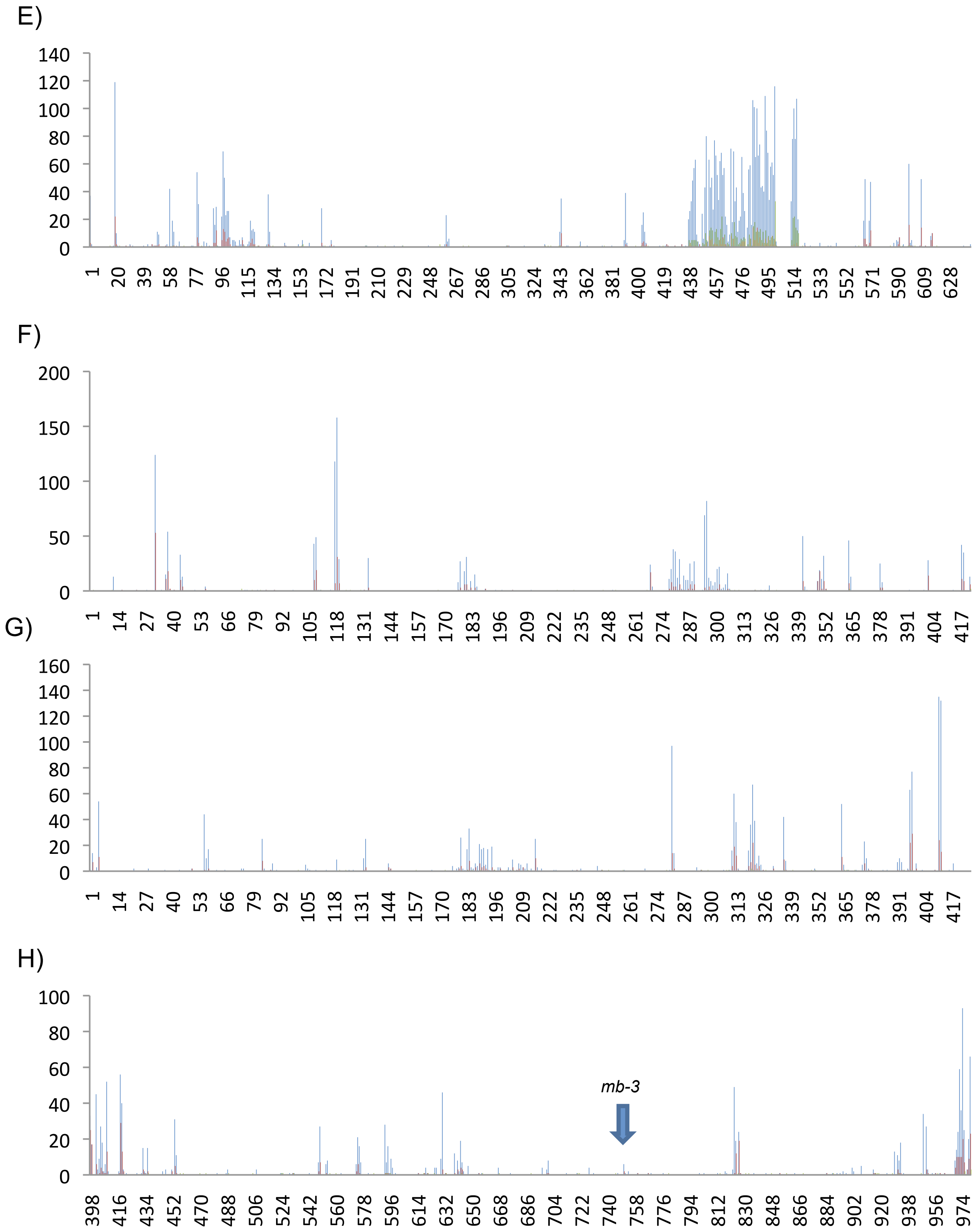

Figure S15 Distribution of polymorphisms in strain 3566. A) Supercontig 1. The location of *mb-3* is indicated by a vertical arrow. B) Supercontig 2, C) Supercontig 3, D) Supercontig 4, E) Supercontig 5, F) Supercontig 6, G) Supercontig 7, H) The region to the right of *cen-1* on Supercontig 1. The location of *mb-3* is indicated by a vertical arrow. Total SNPs are plotted in blue. SNPs that are unique to strain 3566 are plotted in red. Indels are plotted in green. Polymorphisms were sorted by Supercontig and position and the total number in a 10 kb moving window is plotted on the Y axis. The X axis corresponds to the position along the Supercontig. (X 10 kb)
