# Supplementary material for: Rediscovery by Whole Genome Sequencing: Classical Mutations and Genome Polymorphisms in Neurospora crassa
Source: G3 (Bethesda). 2011 Sep 1;1(4):303–16. doi: 10.1534/g3.111.000307 (PMC3276140; doi:10.1534/g3.111.000307)
Supplement: Supporting Information [file supp_1.4.303_FigureS18.pdf]

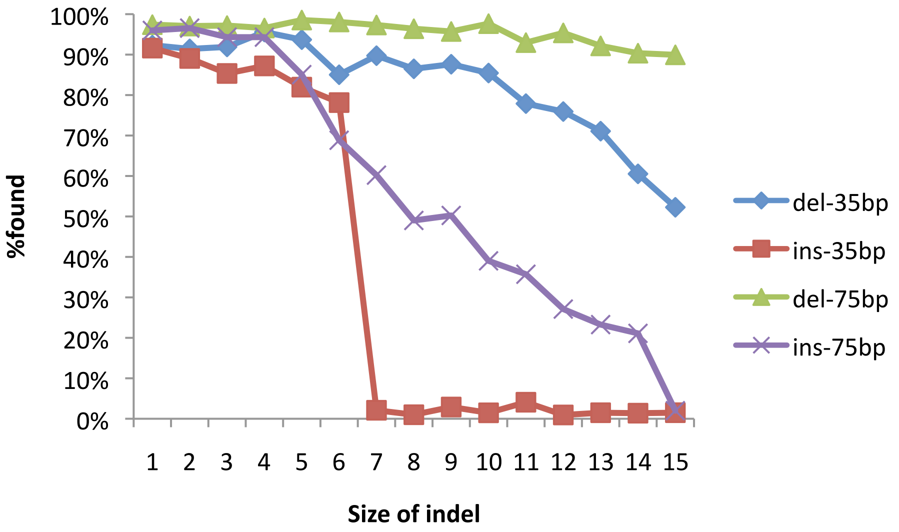

Figure S18 Detection of simulated insertions and deletions in whole genome sequence of Neurospora.
To determine the effect of read length on the ability to identify small indels, we altered the reference of supercont10.1 at evenly spaced intervals to simulate indels, aligned un-altered data to the altered reference, and then determined if maq was able to correctly identify the simulated indels. In general insertions are harder to identify than deletions, and longer reads allow more indels to be identified.
