# Supplementary material for: Rediscovery by Whole Genome Sequencing: Classical Mutations and Genome Polymorphisms in Neurospora crassa
Source: G3 (Bethesda). 2011 Sep 1;1(4):303–16. doi: 10.1534/g3.111.000307 (PMC3276140; doi:10.1534/g3.111.000307)
Supplement: Corrigendum [file supp_1_4_303_v2_index.html]

Corrigendum 

# Rediscovery by Whole Genome Sequencing: Classical Mutations and Genome Polymorphisms in *Neurospora crassa*

## Corrigendum for McCluskey et al. G3 1 (4) 303-316.

**Files in this Data Supplement:**

- Corrigendum for McCluskey et al. G3 1 (4) 303-316.
