# Supplementary material for: Rediscovery by Whole Genome Sequencing: Classical Mutations and Genome Polymorphisms in Neurospora crassa
Source: G3 (Bethesda). 2011 Sep 1;1(4):303–16. doi: 10.1534/g3.111.000307 (PMC3276140; doi:10.1534/g3.111.000307)
Supplement: Corrigendum [file supp_1.4.303_Corrigendum_McCluskey.pdf]

Corrigendum for McCluskey *et al.* *G3* 1 (4) 303-316.

*G3*, Vol. 1, 303-316, September 2011, Copyright © 2011

CORRIGENDUM

In the article by K. MCCLUSKEY, A. E. WIEST, I. V. GRIGORIEV, A. LIPZEN, J. MARTIN, W. SCHACKWITZ, AND S. E. BAKER (*G3* 1: 303-316) entitled "Rediscovery by Whole Genome Sequencing: Classical Mutations and Genome Polymorphisms in Neurospora crassa", a gene is incorrectly labeled.

The ORF NCU02794 is incorrectly referred to as *ham-2* in Table 2 and on page 307. NCU02794 is *ham-1* and *ham-2* is NCU03727. The reference to *ham-1* as being NCU02794 on page 311 is correct. *ham-2* is not relevant to this manuscript.

This error does not impact the interpretation or discussion of the data.
